# Safety and potential efficacy of DM199, a tissue kallikrein-1 analogue, for treating pre-eclampsia and fetal growth restriction: study protocol for a South African, hospital-based phase I/II open-label trial

**DOI:** 10.1136/bmjopen-2025-104035

**Published:** 2025-12-17

**Authors:** Catherine Anne Cluver, Jacquline Thake, Tasleem Hassim, Anriette van Greunen, Samantha Budhram, Karusha Knipe, Eric Decloedt, Lucy Brink, Eduard Langenegger, Lina Bergman, Henrik Imberg, Adrie Bekker, Susan P Walker, Stephen Tong

**Affiliations:** 1 Department of Obstetrics and Gynaecology, Stellenbosch University, Tygerberg Hospital, Cape Town, South Africa; 2 Translational Obstetrics Group, University of Melbourne, Heidelberg, Victoria, Australia; 3 Mercy Perinatal, Mercy Hospital, Heidelberg, Victoria, Australia; 4 Department of Medicine, Stellenbosch University, Cape Town, Western Cape, South Africa; 5 Stellenbosch University, Stellenbosch, Western Cape, South Africa; 6 University of Gothenburg, Gotëborg, Sweden; 7 Statistiska Konsultgruppen, Göteborg, Sweden; 8 Department of Obstetrics, Gynaecology and Newborn Health, University of Melbourne, Heidelberg, Victoria, Australia

**Keywords:** Pregnancy, Clinical Trial, Fetal medicine, Maternal medicine

## Abstract

**Introduction:**

Pre-eclampsia and fetal growth restriction are leading causes of perinatal morbidity and mortality. A therapy that enhances maternal vascular function and promotes vasodilation to increase placental perfusion could treat both conditions.

Tissue kallikrein-1 is an endogenous enzyme that releases bradykinin to activate the bradykinin 2 receptor on endothelial cells. This induces potent vasodilation and pro-angiogenic, anti-oxidant and anti-inflammatory effects.

DM199 is a recombinant form of tissue kallikrein which can be administered intravenously or subcutaneously. Clinical trials in non-pregnant populations have demonstrated its safety. Being a protein, it is unlikely to cross the placenta. This protocol describes an early-phase trial for DM199 for pre-eclampsia and fetal growth restriction.

**Methods and analysis:**

This phase IB/IIA open-label trial at Tygerberg Hospital, Western Cape Province, South Africa, will determine the safety and effective dose of DM199 for pre-eclampsia and/or fetal growth restriction. The trial consists of two parts. Part 1 will be an ascending dose finding study, treating women with pre-eclampsia and severe hypertension who are for planned birth within 72 hours. This will search for doses that safely lower blood pressure (n=3/dose, recruiting up to 42 participants). Part 2 is a safety and efficacy study of three cohorts of pregnant women (n=30/cohort): (1) with pre-eclampsia and severe hypertension requiring delivery within 72 hours, (2) with preterm pre-eclampsia (<33 weeks’ gestation) undergoing expectant management and (3) with preterm fetal growth restriction. Primary outcomes will include adverse events and umbilical cord concentrations of DM199. DM199 pharmacokinetics will be described and exploratory outcomes assessed to evaluate preliminary evidence of efficacy.

**Ethics and dissemination:**

The trial has ethical approval (Health Research Ethics Committee, Stellenbosch University, Protocol number M24/04/009) and is registered (Pan African Clinical Trial Registry, PACTR202404895013782) and approved by the South African Health Products Regulatory Authority (20240801). Data will be presented at international conferences and published in peer-reviewed journals.

**STRENGTHS AND LIMITATIONS OF THIS STUDY**

- This is a protocol of a phase I/II clinical trial to assess the safety and efficacy of a new therapeutic to treat pre-eclampsia and fetal growth restriction.
- We will evaluate pharmacokinetics and obtain multiple safety and efficacy endpoints.
- The sample size is small, so uncommon adverse effects may be missed.
- There is no placebo arm.

## Introduction

Pre-eclampsia is a serious complication affecting 5–7% pregnancies.^1^ For every maternal death related to pre-eclampsia, another 50 to 100 women suffer severe health injuries. It is estimated that pre-eclampsia is associated with more than 25% of all severe maternal outcomes and that it is the direct cause of 20% of reported maternal deaths.^2^ Fetal growth restriction commonly arises from placental insufficiency and is a major risk factor for stillbirth and serious adverse perinatal outcomes.^3^ Once diagnosed, there are no disease-modifying treatments that slow or reverse disease progression of either condition, apart from delivery. A therapy that directly targets the disease pathogenesis of either could be lifesaving.

Both pre-eclampsia and fetal growth restriction arise from poor placental function due to reduced placental perfusion, histopathologically evident as maternal vascular malperfusion injuries.^4^ Pre-eclampsia is further characterised by endothelial dysfunction^1 5^ and maternal vascular injury. This leads to hypertension and vasoconstriction of vessels, which damages many end organs supplied by these vessels.^1^ Pre-eclampsia is associated with placental and systemic inflammation, oxidative stress and an anti-angiogenic state.^6^ Hence, a drug that dilates blood vessels to improve organ and placenta perfusion and promotes vascular health (via pro-angiogenesis and reductions in inflammation and oxidative stress) may be a treatment for both pre-eclampsia and fetal growth restriction.^6^

Tissue kallikrein (KLK1) is an endogenous enzyme produced and secreted from endothelial cells and potentially other tissues, such as the kidneys. DM199 is a pharmaceutical formulation comprised of recombinant KLK1. It is a protein drug identical to KLK1, except for two inert amino acid substitutions to increase stability. It can be administered as a short-acting intravenous infusion (approximately a 4-hour half-life) or as a subcutaneous injection with an extended half-life of approximately 50–60 hours.

KLK-1 cleaves kininogen to release the peptide bradykinin. Bradykinin then activates the bradykinin 2 receptor on the blood vessel endothelium^7^ to reduce blood pressure (BP)^8^ by upregulating the production of three potent vasodilators: nitric oxide,^9^ prostacyclin and endothelium-derived hyperpolarising factor. These signalling molecules relax the underlying smooth muscle in blood vessels to promote vasodilation.

Soluble fms-like tyrosine kinase-1 (sFlt-1), a central disease driver of pre-eclampsia, blocks the activation of vascular endothelial growth factor receptor 2 (VEGFR2) on endothelial cells, leading to endothelial and blood vessel dysfunction. Bradykinin signalling may potentially overcome the sFlt-1 block which is an important part of pre-eclampsia pathogenesis.^5^ Molecular studies have shown that activation of the bradykinin 2 receptor facilitates VEGFR2 signalling by increasing the production of both VEGFR2 protein and its signalling molecule, VEGF.^10^ Furthermore, bradykinin 2 receptor activation relays signals within endothelial cells that circle back to VEGFR2 on the cell surface and activates it, a process called transactivation.^11^ These actions could have the effects of bypassing sFlt-1 antagonism present in pre-eclampsia. Supporting these molecular findings is a body of literature showing KLK1 is centrally involved in facilitating angiogenesis.^10 12 13^ This includes preclinical models that suggest KLK-1 mediated angiogenesis is not impaired by the presence of sFlt1.^14^

The downstream intracellular molecules switched on by the bradykinin receptor 2 may also reduce inflammation,^15^ upregulate antioxidant defences^16 17^ and increase insulin sensitivity, glucose uptake and glycogen synthesis (reducing levels of glucose in the bloodstream, a contributor to endothelial dysfunction).^18^ All these actions may further reduce endothelial dysfunction, reducing disease severity over and above simply lowering BP.

Furthermore, the vasodilatory properties as a result of DM199 administration might enhance perfusion to the placenta. If so, DM199 could have merit in treating not only pre-eclampsia, but also fetal growth restriction.

DM199 is a protein, making it unlikely to cross the placenta and reach the fetus. This is because the syncytiotrophoblast, the surface layer of the placenta, acts as a diffusional barrier. While small molecules such as glucose can passively diffuse across the syncytiotrophoblast, macromolecules such as proteins can only cross if there are specific carrier transport proteins.^19^ A further barrier to proteins reaching the fetal circulation is a non-fenestrated endothelium lining fetal vessels^19 20^: large molecules also require active (vesicular) transport to cross through into the fetal circulation.^20^ However, the premise that DM199 cannot cross the placental barrier and reach the fetal circulation needs to be properly evaluated.

These properties make DM199 an appealing candidate for treating obstetric conditions, as it may exert multiple beneficial effects without crossing into the placenta. If this is true, this may reduce safety concerns related to direct fetal exposure. However, the premise that DM199 cannot cross the placental barrier and reach the fetal circulation needs to be properly evaluated.

Small early phase published clinical trials^21 22^ and other completed trials (NCT01845064, NCT03290560, NCT04123613, online supplemental table 3 lists all DM199 trials) in non-pregnant populations report that DM199 can be safely administered to humans and seems well tolerated. Clinical trials from China suggest the urinary form of KLK1 may be beneficial in treating stroke (summarised in Kasner *et al*).^23^ Indeed, KLK-1 extracted from the urine has been approved in China to treat acute ischaemic stroke, where it has been estimated (on publicly available sales figures) that 500 000 are being treated annually.^24^

Hence, DM199 possesses several properties that make it a strong candidate to treat pre-eclampsia and fetal growth restriction. We propose conducting a phase I/II open-label, single-arm clinical trial to determine an effective BP-lowering dose of DM199 in women diagnosed with pre-eclampsia, followed by assessments of safety and tolerability. Additionally, we will perform pharmacokinetic profiling as this is its first use in pregnant women. Finally, we explore various outputs to gather preliminary evidence of efficacy.

## Objectives

The primary objectives are (1) to identify doses of DM199 that effectively reduce blood pressure and can be safely administered to pregnant women with pre-eclampsia and (2) to evaluate safety and tolerability and obtain exploratory evidence of efficacy of DM199 in pre-eclampsia and/or fetal growth restriction.

## Methods

### Overview of study design and trial population

This trial will be an open-label, phase IB/IIA single-centre study conducted at Tygerberg Hospital, a major tertiary referral centre in Cape Town, South Africa. The study population consists of pregnant women diagnosed with pre-eclampsia and/or fetal growth restriction who have been admitted to inpatient care.

The trial will be divided into two parts. Part 1 will be a dose-finding study enrolling up to 42 women with pre-eclampsia. Part 2 will comprise three cohorts, each enrolling 30 participants: Cohort 2.1 will be women with acute hypertension for planned birth within 72 hours; Cohort 2.2 will be women with preterm pre-eclampsia undergoing expectant management, and Cohort 2.3 will be women with preterm fetal growth restriction undergoing expectant management.

General inclusion criteria across all cohorts are shown in box 1. As the cohorts differ, each will have specific inclusion criteria and distinct study outcomes, which are detailed in online supplemental table 4. The clinical trial schedule and grading of adverse events are shown in online supplemental tables 1 and 2. The complete study protocol is available in the online supplemental information.

Box 1 **General inclusion and exclusion criteria**

**Inclusion criteria**

- Viable singleton pregnancy.
- Diagnosis of pre-eclampsia and/or fetal growth restriction.
- Admitted for inpatient management until delivery.

**Exclusion criteria**

- Unable, or unwilling to give consent, or is under the age of 18.
- Hypotension, defined as a systolic blood pressure (BP) <90 mm Hg and/or a diastolic BP <60 mm Hg and/or a mean BP <65 mm Hg.
- Severe complications of pre-eclampsia which include eclampsia, stroke, pulmonary oedema, HELLP syndrome (haemolysis, elevated liver enzymes, low platelets), severe renal involvement and liver rupture or haematoma.
- Clinical infection, for example, chorioamnionitis.
- Underlying maternal cardiac disease including a significant arrhythmia, a conduction abnormality or severe valvular disease or congenital or acquired heart disease.
- Significant maternal vascular disease, for example, renal artery stenosis.
- No adequate maternal venous access for sampling.
- Patient is unable, or unwilling to give consent, or aged <18 years.
- Suspicion of a major fetal anomaly or malformation or chromosomal abnormality. A major fetal anomaly is defined as anomalies or malformations that create significant medical problems for the patient or that require specific surgical or medical management.
- Fetal compromise that necessitates urgent delivery.
- Cerebrovascular event, defined as an ischaemic or haemorrhagic stroke associated with clinical symptoms and definitive signs on imaging.
- Liver haematoma or rupture.
- Placental abruption.
- Placenta accreta spectrum disorder.
- History of clinically significant allergic reactions such as angioedema or anaphylaxis requiring hospitalisation, or familial angioedema.
- Participant is currently participating in or has participated in a study using an investigational device or drug or received an investigational drug or investigational use of a licensed drug within 30 days prior to screening.
- Women with an active malignancy.

### Sample size

For the ascending dose finding study (part 1), sequential cohorts of three subjects at each dose will be enrolled as is standard for dose finding studies.^25^ Up to 10 doses will be tested. If a therapeutic dose is discovered, a confirmation cohort of up to 12 women will be included.

For part 2 of the study (safety, tolerability and pharmacokinetic assessment), 30 women will be included in each of the three sub-cohorts. The sample size of investigational phase 2 drug trials tends not to be large, and 10–40 cases per group is usually the standard.^26^

### Part 1: an open label phase 1B ascending dose finding study

We will recruit up to 42 women diagnosed with pre-eclampsia and acute hypertension, defined as systolic BP≥150 mm Hg and/or a diastolic BP≥110 mm Hg, and are scheduled for birth within 72 hours. The decision for delivery is a clinical decision based on international guidelines.^27^

DM199 will be administered as an initial intravenous loading dose, followed by a subcutaneous injection designed to provide an equivalent dose with sustained effect over 3 days. However, for the first dose escalation step, participants will receive only an intravenous dose, without subsequent subcutaneous administration.

Dose escalation will proceed in sequential groups of three participants (up to 10 dose levels, see table 1) until an optimal dose is found that safely reduces BP to a therapeutic target of <140 mm Hg systolic and <100 mm Hg diastolic. As this is a first in pregnancy study, we will start with an extremely low dose of DM199. Based on previous studies in non-pregnant individuals, significant BP decreases are expected to occur when the dose is 1.5 µg/kg or higher. To be cautious, we will be starting with a dose that is 15 times lower than this (0.1 µg/kg). The Food and Drug Administration has approved a maximum subcutaneous dose of 15 µg/kg for human administration, and we will not exceed this limit. The subcutaneous dose that is equivalent to the intravenous dose is six times higher. The subcutaneous dose is being added as part of the protocol as the half-life of the intravenous dose is short. If tolerated, we will then sequentially increase the dose until a clinical response is seen (a blood pressure reduction) or a maximum dose is reached.

**Table 1:** DM 199 dosages for part 1 dose finding study

Cohort (each cohort will include three participants)	Intravenous DM199 dose (µg/kg)*	2-hour SC DM199 dose (µg/kg)†
1	0.1	None
2	0.1	0.6
3	0.2	1.2
4	0.5	3
5	0.75	4.5
6	1.0	6
7	1.25	7.5
8	1.5	9
9	2	12
10	2.5	15

* Maximum intravenous dose will not be higher than 2.5 µg/kg.

† Maximum subcutaneous dose will not be higher than 15 µg/kg.

Dose escalation will be halted if the maximum planned dose is reached or if dose-limiting toxicity occurs.

Once a well-tolerated dose that effectively lowers blood pressures to the therapeutic target is identified, we will re-evaluate this dose in an additional 12 participants—six participants with preterm pre-eclampsia (<34 weeks gestation) and six with pre-eclampsia at or beyond 34 weeks gestation. This will assess whether the selected dose consistently lower blood pressures to the therapeutic target (<140/100 mm Hg) across gestational age subgroups, supporting further investigation in part 2. Recruiting these extra 12 participants will provide greater confidence in identifying the appropriate dose for sub-cohorts in part 2. Hence, the maximum number of participants we may recruit for part 1 is 42.

The primary outcome will be safety and tolerability, including the incidence of acute hypo- or hypertension, early onset of uterine contractions or acute allergic reactions. Continuous fetal heart rate monitoring will be performed until delivery. Additionally, intensive pharmacokinetic sampling will be conducted in the mother to evaluate DM199 exposure. Transplacental transfer will be assessed by measuring DM199 levels in the umbilical cord blood at birth, and in breastmilk samples collected within 24 hours postpartum. Finally, for part 1, we will collect data for preliminary evidence of efficacy, including Doppler ultrasound of the uterine arteries (as reduced vascular resistance may reflect increased placental perfusion) and fetal vessels, and whether there are any changes in clinical biomarkers indicating disease severity.

### Part 2: an open label phase II study with three sub-cohorts

Part 2 will recruit and enrol a total of 90 participants, divided into three sub-cohorts of 30 participants each. Each sub-cohort will assess specific outputs related to safety, tolerability and efficacy in the sub-cohorts that are relevant to their condition:

- Sub cohort 2.1: 30 women with pre-eclampsia with acute hypertension, defined as a systolic blood pressure ≥150 mm Hg and/or a diastolic blood pressure ≥110 mm Hg, that require delivery within 72 hours.
- Sub-cohort 2.2: 30 women with preterm pre-eclampsia (<33 weeks’ gestation) and assessed as suitable for expectant management.
- Sub-cohort 2.3: 30 women with preterm fetal growth restriction and suitable for expectant management.

In addition to ongoing safety and tolerability assessments, we will collect preliminary efficacy data on blood pressure reductions and other clinical outcomes. We will also perform further pharmacokinetic studies. Specific inclusion, exclusion criteria and outputs for the sub-cohorts are shown in online supplemental table 4.

The selected doses may vary across sub-cohorts. For instance, we anticipate that acutely hypertensive participants in cohort 2.1 will need a higher dose than cohort 2.3, who have a preterm fetal growth restriction where the mother may be normotensive (in cohort 2.3, the aim is to identify a DM199 dose that vasodilates vessels supplying the uteroplacental unit without dropping the maternal blood pressure to hypotensive levels). Doses for the sub-cohorts will be selected a priori in consultation with an independent data and safety monitoring committee. Cohorts 2.1 and 2.2, hypertensive cohorts, will receive an initial intravenous dose of DM199 to achieve rapid blood pressure control, followed by subcutaneous dosing. For cohorts 2.2 and 2.3 (expectant management groups), serial subcutaneous doses of DM199 will be administered as long as the pregnancy continues.

For the three populations, common primary outcomes will include safety and tolerability, and the quantification of DM199 concentrations in the umbilical cord at birth.

Preliminary evidence of efficacy will be evaluated through specific outcomes for each sub-cohort. For cohort 2.1, efficacy outcomes will be similar to those in part 1. For cohort 2.2 (preterm pre-eclampsia), efficacy outcomes will include prolongation of pregnancy, changes in the spot urinary protein:creatinine ratio a week after enrolment compared with baseline and the need to increase or decrease other anti-hypertensive agents. For cohort 2.3 (preterm fetal growth restriction), efficacy outcomes include changes in fetal Doppler parameters and birth weight centile at birth.

Secondary and exploratory outcomes for the three cohorts vary and are described in online supplemental table 4 and in the full protocol (supplementary materials). In brief, they range from sparse pharmacokinetic sampling, changes in clinical markers associated with disease severity, blood biomarkers associated with pre-eclampsia or fetal growth restriction, adverse perinatal outcomes and physiological changes in the maternal vasculature, such as changes in flow mediated blood vessel dilatation and changes in cerebral autoregulation.

### Inclusion, exclusion criteria and definitions

Inclusion and exclusion criteria are presented in box 1 and table 1. All participants will already be admitted for inpatient hospital management at Tygerberg Hospital. At our centre, pregnancies are only considered viable (and therefore eligible for inclusion) if the gestational age is ≥27 weeks 0 days and the estimated fetal weight is above 750 g. Gestational age will be determined by an early or mid-trimester pregnancy ultrasound. If the gestational age is uncertain (eg, women presenting late for antenatal care), we will estimate the gestational age using ultrasound according to the International Society of Ultrasound and Gynaecology Guidelines.^28^

Pre-eclampsia will be defined according to the International Society for the Study of Hypertension In Pregnancy (ISSHP),^27^ but will also require the presence of significant proteinuria (≥300 mg in a 24-hour urine collection or a spot protein:creatinine ratio ≥30 mg/mmol or ≥1+ proteinuria on dipsticks). Fetal growth restriction will be classified according to a recent Delphi consensus,^29^ but will also require the estimated fetal weight to be less than the third centile on fetal growth charts. Specific inclusion and exclusion criteria for each part of the study are presented in box 1.

### Trial drug

DM199 is a pharmaceutical formulation composed of highly purified recombinant human KLK1 (US patent no 9,364, 521 B1). DM199 is the same sequence as KLK1 (a protein with 238 amino acids) except for amino acid substitutions at positions 145 and 188 to increase stability. The DM199 sequence has an E to Q change at the position 145 and an A to V at position 188 of the pro-protein, both of which are away from the active site.

The manufacturer commissioned animal toxicology and reproductive toxicology studies as part of the path to obtaining regulatory drug approval. The toxicology studies indicate that DM199 is safe and well tolerated at doses 20-fold higher than expected human therapeutic doses. Reproductive toxicology studies in two animal species have been completed using DM199 doses that are significantly higher than the proportionate dosing planned for humans. These studies showed no adverse effects on fetal development, offspring (followed for two generations) or future fertility. In rats, DM199 concentrations were detected in the maternal blood when administered at day 17, but remained undetectable in fetal blood, suggesting no placental transfer.

DM199 has been administered to human participants across seven clinical trials (see online supplemental table 3). Six of these trials are completed, of which two are published.^21 22^ There is one ongoing trial running, REMEDY2, which is a large, multi-international adaptive platform trial assessing the efficacy of DM199 to treat acute ischaemic stroke in candidates that are unsuitable for mechanical thrombectomy. As noted, KLK1 extracted from the urine (the natural version of DM199) is approved to be used to treat acute ischaemic stroke in China.

DM199 appears to be well tolerated. The most common adverse event following subcutaneous administration is headache. Other reported events include nausea, postural dizziness and injection orthostatic hypotension and injection site reactions. However, this will be the first investigation of DM199 in pregnant women.

Regarding contraindications, DM199 may increase bradykinin activity resulting in systemic vasodilation. Therefore, it should not be administered to those who are hypotensive. Notably, DM199 at therapeutic doses in clinical trials done to date suggests that the drug does not decrease BP in normotensive individuals. DM199 should not be initiated in participants who received an angiotensin-converting enzyme inhibitor within 24 hours as angiotensin-converting enzyme degrades bradykinin (hence, its inhibition overly potentiates bradykinin signalling, and could cause significant hypotension). However, angiotensin-converting enzyme inhibitors are contraindicated in pregnancy, meaning this is unlikely to be a concern.

### Study procedures

Potential participants will be identified after they have been admitted to Tygerberg Hospital with a diagnosis of pre-eclampsia or fetal growth restriction. Research midwives working in the labour ward will identify possible participants. Screening will be done according to inclusion and exclusion criteria. Written, informed consent will be taken by a research nurse or study team member. The clinical staff providing clinical care will not be involved in recruiting participants. Information sheets and study details will be provided and any questions about the study will be answered. A translator will be used so that potential participants will have the opportunity to go through the information in her own language (The information leaflet and consent forms have been translated into local languages).

The appropriate dose of DM199 will be calculated using the body weight (kg) and then drawn up in a syringe as per the instructions in the study Pharmacy Manual. Drug preparation will be performed using sterile aseptic techniques. The intravenous dose will be diluted in a PVC Baxter VIAFLEX 50 mL infusion bag and administered via PVC tubing. The infusion will be started at 35 mL/hour for the first 15 min. The intravenous study drug infusion will be increased to 75 mL/hour to complete the 50 mL intravenous infusion in approximately 50 min if the blood pressure remains above the optimal target. Subcutaneous injections will be on the lateral side of the thighs or arms, with injection sites rotated. If urgent delivery is required for maternal or fetal indications or if any serious side effects develop, the medication infusion will be stopped immediately.

Participants will be seen daily by study nurses until they deliver. Blood pressure, heart rate and uterine activity will be measured and documented, as well as fetal ultrasound and maternal Doppler studies, endothelial function and cerebral autoregulation, as detailed in online supplemental table 1. Ultrasound examinations (measuring maternal and fetal Dopplers) will be performed twice a week or as clinically indicated. Endothelial function testing and cerebral autoregulation measurements will be repeated once a week until delivery and then once postpartum (online supplemental table 1).

Participants will remain under the care of the Obstetrics Department at Tygerberg Hospital, who will make clinical decisions. The study will not interfere with routine care, including the decision to deliver the pregnancy. Standard antihypertensive medications, magnesium sulphate, corticosteroids and all other medications will be administered according to local protocols except that other blood pressure medications will be withheld or decreased if the blood pressure is within target range (titrated to ongoing blood pressure levels in participants). Most participants will be on antihypertensive treatment, and the dose and number of antihypertensives will be recorded.

### Data management

Data will be collected prospectively using a REDCap database hosted at Stellenbosch University.^30^ Participant personal information will be pseudonymised to protect confidentiality. All data entries will undergo rigorous quality control, including double-checking for inaccuracies. Copies of the source documents, including the antenatal card and hospital notes, will be securely uploaded to REDCap for comprehensive record-keeping and verification.

### Duration of the trial

The trial is expected to be completed within 2 years, spanning 2025–2026.

### Statistical methodology

This open-label investigation of DM199 is primarily intended to assess safety, with an exploratory component to identify possible early signs of efficacy. Statistical analyses will be primarily descriptive in nature.

Continuous variables will be summarised using number of values (N), mean, SD, median, minimum and maximum values. Categorical variables will be presented using frequency counts and percentages per category. Missing data will not be imputed but will be analysed as missing. CIs will be two-sided and with 95% confidence levels unless otherwise specified.

Baseline values will be defined as the last observation recorded for each participant prior to DM199 administration. Changes from baseline in vital signs at each timepoint will be summarised by study cohort and dose using descriptive statistics.

### Pharmacokinetic analysis

For each participant, the following pharmacokinetic parameters will be calculated from the concentration time-course data: AUC_0-t_ (area under the curve from time 0, the time of dosing, to the last measurable time point), AUC_0-∞_ (AUC extrapolated to infinity), C_max_ (maximum observed concentration), AUC_0-24_ (AUC from time 0 to 24 hour post-dose), T_max_ (time at which Cmax is first observed), T_1/2_ (apparent elimination half-life), CL/F (apparent clearance) and Vz/f (apparent oral volume of distribution). Both arithmetic and geometric mean values will be used to describe the combined results from all participants within each dose group. Pharmacokinetic data will be analysed alongside efficacy endpoints to explore potential exposure-response relationships.

### Trial governance

This is an investigator-initiated trial, sponsored by University of Stellenbosch and funded by Diamedica Therapeutics, which is also providing the investigational drug. All data will be securely stored at the University of Stellenbosch. An independent Data and Safety Monitoring Committee will oversee the trial, with the authority to suspend or cease the trial if safety concerns arise. An independent trial monitor will be appointed.

Some investigators involved in this study have provided consulting services for Diamedica Therapeutics. The investigators designed the protocol with input from Diamedica Therapeutics. The investigators will not require Diamedica Therapeutics permission to publish the final manuscript.

### Adverse events

An adverse event is defined as any untoward medical occurrence associated with the use of a drug in humans, whether or not it is considered drug related. A serious adverse event is defined as a maternal death, a fetal loss or neonatal death, an event that results in a longer postnatal hospital stay, an event that results in a persistent or significant disability in the mother or baby and/or a congenital or birth defect in the baby that is detected in the post-natal period and was not detected on ultrasound. All adverse events will be documented and reported on data capture sheets and transcribed to electronic case report forms. All adverse reactions will be reported to the principal investigator within 24 hours. The principal investigator will be responsible for grading adverse events in online supplemental table 2. If an adverse event occurs that is not listed, it will be graded using the latest version of the Common Terminology Criteria for Adverse Events (https://ctep.cancer.gov/protocoldevelopment/electronic_applications/docs/ctcae_v5_quick_reference_5x7.pdf). Maternal and fetal adverse events will be graded using the Maternal and Fetal Adverse Event Terminology V.1.1, 2022 or later versions.^31^ Neonatal adverse events will be graded using the Neonatal Adverse Event Severity Scale.^32^ Serious adverse events will be reported to the ethics committee, the data monitoring and safety committee, the drug manufacturer and the South African Health Products Regulatory Authority within 24 hours of the principal investigator being notified.

Monitoring for adverse events will occur from the time of signing informed consent until the end of the follow-up period.

### Patient and public involvement

We have run a study assessing women’s perspectives on types of therapeutics and what they view as important outcomes. In small group interactions, women have expressed that the most important outcome is the health of their baby (unpublished data). Women have expressed that they would be willing to test a new drug if it could potentially improve the health of their unborn baby.

### Ethics and dissemination

This trial of DM199 for pregnancy complications has ethical approval (Health Research Ethics Committee, Stellenbosch University (Protocol number M24/04/009). It is registered with the Pan African Clinical Trial Registry (29416) and the South African Health Products Regulatory Authority (20240801). Findings from this study will be presented at international conferences and published in peer-reviewed journals.

## Discussion

We describe a protocol for an early phase trial to study DM199 as a novel treatment for pre-eclampsia and potentially fetal growth restriction. This trial will be the first time DM199 is administered to a pregnant population.

DM199 is a pharmaceutical analogue of KLK1, a natural enzyme that releases the peptide bradykinin from its pro-form.^12^ Bradykinin signals via its cognate receptor on endothelial cells to induce potent blood pressure lowering effects and vasodilate vessels^8^ which could increase end organ perfusion which is compromised in pre-eclampsia. It may also increase placental perfusion. If so, it may mean DM199 can ameliorate both pre-eclampsia and fetal growth restriction at their source.

The advantage of a drug that is an analogue of a natural protein is that there are already decades of pre-existing basic science research. Numerous studies suggest administering KLK1 to release bradykinin may have other actions favouring vascular health beyond simple vasodilation and blood pressure lowering. These include pro-angiogenesis, reducing oxidative stress^16 17 33^ and inflammation^34^ and even reducing hyperglycaemia. These actions could be favourable in arresting the disease progression of pre-eclampsia because endothelial and vascular dysfunction is an important disease driver.

Interestingly, KLK1 isolated from urine has been approved by the Chinese State Food and Drug Administration to be used to treat acute ischaemic stroke^23^. It has been used clinically for this indication for over 15 years. Chinese researchers have reported KLK1 may be reduced in pre-eclampsia and of exogenously administered KLK1 derived from porcine pancreas suggests tissue kallikrein-1 could beneficially treat pre-eclampsia. However, these trials have potential methodological limitations and merit validation.

We propose an early phase trial of DM199, a pharmaceutical analogue of KLK1. Our trial has sequential steps. The first part is dose-finding studies where we will monitor safety with dose increases but also search for a dose that can reduce blood pressure to therapeutic levels. Blood pressure lowering will be our first early indication of efficacy. We anticipate finding blood pressure reductions before reaching the maximum planned dose escalation step because prior trials of DM199 in non-pregnant populations have shown the drug reduces blood pressure at subcutaneous doses from around 2.0 μg/kg. For this part 1 dose escalation step, we chose to investigate a population with severe hypertension and for planned birth within 72 hours, after blood pressure stabilisation. We believe this represents an ideal cohort for a first in pregnant population study of an investigation drug because should there be unanticipated side effects mandating emergent birth, adverse consequences are minimised. Also, it is a suitable population to initially establish whether DM199 enters the fetal circulation because birth will occur shortly after administration. Given we are targeting a population for planned birth, we will recruit participants at all gestational ages for part 1.

Part 2 expands the investigation to three patient populations that may potentially benefit from DM199. For part 2, we will obtain a range of outputs related to safety and potential efficacy, both outputs that are common to all cohorts (box 1), and others that are specific to sub-cohorts (online supplemental table 4).

Strengths of our study are the sequential design which we believe maximises participant safety, should there be unanticipated adverse effects. A further strength is that we will perform embedded pharmacokinetic studies, an element often lacking in trials of drugs for obstetric conditions; and obtain many exploratory outcomes to search for early signals of efficacy. The main limitation is that it is a single arm trial and will not be powered to prove efficacy other than blood pressure reduction within participants. Furthermore, with a limited number of participants, this trial will miss uncommon adverse effects. As such, further large, randomised trials will be needed.

A theoretical advantage of DM199 is that being a protein, it is unlikely to cross the placenta as it is too big to breach the cell surface of the placenta. Proteins that cross the placenta, notably IgG antibodies, only do so because there is an active transport mechanism.^35^ Even single amino acids require a transport mechanism to cross the placenta.^36^ Rat studies commissioned by Diamedica Therapeutics demonstrated a rise of DM199 in the maternal circulation, but concentrations in the umbilical cord were undetectable, suggesting no transfer into the fetal circulation. Nevertheless, we will confirm this by measuring DM199 concentrations in the maternal circulation and fetal blood via a sample from the umbilical cord taken at birth.

Another potential advantage of DM199 is that being an analogue of a naturally occurring protein, it may be highly acceptable to clinicians and consumers should efficacy be proven. This is because nearly all drugs used in obstetrics are also versions of naturally occurring molecules, notably progesterone to prevent preterm birth,^37^ prostaglandins to prime the cervix in preparation for labour,^38^ oxytocin to switch on active labour, corticosteroids to accelerate fetal maturity in preparation for birth and magnesium sulphate for fetal neuroprotection and to prevent eclampsia.

Limitations of the study include the small sample size and uncommon side effects of the drug that may therefore be missed. As this is a dose-finding study to assess safety and efficacy, there is no placebo arm. Potential risks of the use of DM199 include a possible release of prostaglandin E2. If prostaglandin E2 were secreted into the blood in biologically relevant amounts, it may result in cervical ripening and uterine contractions. For this reason, for part 1, we will include only women where the decision for delivery has been made. DM199 increases nitric oxide production. This may have a pro-inflammatory response, so we will monitor for an inflammatory response. There is also a chance that DM199 may cause hypotension resulting in fetal distress. We will therefore closely monitor both maternal and fetal condition.

Drugs to treat pre-eclampsia and fetal growth restriction are urgently needed as they remain two of the most serious diseases of pregnancy. While aspirin has been shown to prevent preterm pre-eclampsia,^39^ no drugs exist that arrest or reverse disease pathogenesis once pre-eclampsia^1^ or fetal growth restriction^40^ is diagnosed.

Furthermore, there has been a dearth of treatment trials of investigational agents aimed at the disease pathogenesis for pre-eclampsia and fetal growth restriction. Sildenafil, which augments nitric oxide signalling, was not found to be beneficial to treat fetal growth restriction^41 42^ and potentially caused fetal harm.^43^ An earlier trial of oral sildenafil suggested potential benefit for pre-eclampsia,^44^ but in light of its possible neonatal risks, it is unlikely to be pursued further as a pre-eclampsia treatment. For pre-eclampsia, randomised trials of anti-thrombin III^45^ and oral esomeprazole^46^ have reported negative outcomes. A phase II randomised trial suggests metformin may prolong pregnancy for preterm pre-eclampsia,^47^ and this finding is being validated in concurrent randomised trials in South Africa (PACTR202104532026017), Sweden (EU trial no. 2022-502707-2-00) and the Netherlands (EU trial no 2023-510382-10-00). While many studies have reassuringly shown that metformin does not impact on long-term childhood outcomes,^48^ other studies have raised safety concerns.^49 50^ The concerns likely stem from the fact there is known transplacental passage of metformin.^51^ It highlights the strong potential advantage of DM199 in terms of perceived risk profile and acceptability.

In summary, there are no treatments to slow disease progression of pre-eclampsia or fetal growth restriction. Furthermore, there are currently very few active trials of novel investigational agents for these conditions. Our proposed trial is the first step to seeing whether DM199 might be such a new treatment for pre-eclampsia and/or fetal growth restriction.

## Supplementary material

10.1136/bmjopen-2025-104035 online supplemental file 1

10.1136/bmjopen-2025-104035 online supplemental file 2

10.1136/bmjopen-2025-104035 online supplemental file 3
