# Supplementary material for: Safety and potential efficacy of DM199, a tissue kallikrein-1 analogue, for treating pre-eclampsia and fetal growth restriction: study protocol for a South African, hospital-based phase I/II open-label trial
Source: BMJ Open. 2025 Dec 17;15(12):e104035. doi: 10.1136/bmjopen-2025-104035 (PMC12716557; doi:10.1136/bmjopen-2025-104035)
Supplement: online supplemental file 1 [file bmjopen-15-12-s001.pdf]

DM199 for pregnancy complications

Phase I/II safety and efficacy trial of DM199 for treating preeclampsia and fetal growth restriction

DM199 for Pregnancy Complications

Phase I/II study

CLINICAL TRIAL PROTOCOL Version 1.1

**Full title of trial**	DM199 for Pregnancy Complications<br>Phase II A proof of concept trial assessing DM199 as a therapeutic for preeclampsia and fetal growth restriction
**Short title**	DM199 for Pregnancy Complications
**Version and date**	1 March 2025, Version 1.1
**Ethics approval**	Date: 26 June 2024<br>Protocol number: Project Id: 30213<br>Ethics Reference No: M24/04/009<br>Federal Wide Assurance Number: 00001372<br>Institutional Review Board (IRB) Number: IRB0005239
**Pan African Clinical Trial Registry (PACTR) ID**	29416
**South African National Clinical Trial Registry (SANCTR)**	Trial ID 9535
**South African Health Products Regulatory Authority**	20240801<br>Approval date: 8 October 2024
**National Health (NHRD) Registration**	https://nhrd.health.gov.za/Proposal/PHRCWizard/126910
**Trial medication**	DM 199 (Recombinant human tissue kallikrein)
**Phase of trial**	Phase I/II
**Principal investigator**	Prof Catherine Anne Cluver
**Trial statistician**	Dr Henrik Imberg
**Co-investigators**	Prof Stephen Tong<br>Prof Susan Walker<br>Dr Samantha Budhram<br>Prof Adrie Bekker<br>A/Prof Lina Bergman<br>Eric Decloedt<br>Dr Henrik Imberg
**Funders**	Investigator-sponsored grant from DiaMedica Therapeutics Inc.<br>301 Carlson Parkway, Suite 210<br>Minnetonka, MN 55305 USA<br>Tel: (763) 312-6064
**Sponsor**	Stellenbosch University

Investigator Contact Details

Principal Investigator for clinical trial
Prof Catherine Cluver
Tygerberg Hospital, University of Stellenbosch
Address: Preeclampsia Research Institute, Department of Obstetrics and Gynaecology, Stellenbosch University, Francie van Zyl Drive, Tygerberg, Cape Town, South Africa 7505
Tel: +27823210298
Email: cathycluver@hotmail.com

Co-Investigators
Prof Stephen Tong
Mercy Hospital for Women
University of Melbourne
Tel: +613 8458 4380
Email: stong@unimelb.edu.au

Prof Susan Walker
Mercy Hospital for Women
University of Melbourne
Tel: +613 84584380
Email: spwalker@unimelb.edu.au

Dr Samantha Budhram
Tygerberg Hospital
University of Stellenbosch
Tel: +27 832327617
Email: samant@sun.ac.za

Dr Tasleem Hassim
Tygerberg Hospital
University of Stellenbosch
Tel: +27 835577292
Email: tasleemhassim1@gmail.com

Dr Karusha Knipe
Tygerberg Hospital
University of Stellenbosch
Tel: +27 825518283
Email: kknipe@sun.ac.za

Prof Adrie Bekker
Tygerberg Hospital
University of Stellenbosch

Tel: +27 21 938 9198
Email: adrie@sun.ac.za

A/Prof Lina Bergman
Tygerberg Hospital
Stellenbosch University
University of Gothenburg
Email: lina.bergman@obgyn.gu.se

Clinical Pharmacologist

A/Prof Eric Decloedt
Stellenbosch University
Email: ericdecloedt@sun.ac.za

Trial Design and Statistics

Dr Henrik Imberg
Email: henrik.imberg@stat-grp.se
Statistiska Konsultgruppen, Gothenburg, Sweden
University of Gothenburg

Laboratory Coordination

South Africa:

Prof Catherine Cluver
Tygerberg Hospital
University of Stellenbosch
Tel: +27823210298
Email: cathycluver@hotmail.com

Australia:

Prof Stephen Tong
Mercy Hospital for Women
University of Melbourne
Tel: +613 8458 4380
Email: stong@unimelb.edu.au

Trial Committees

Trial Steering Committee

Chair
Prof Catherine Cluver
Tygerberg Hospital
University of Stellenbosch
Tel: +27823210298
Email: cathycluver@hotmail.com

Members
Prof Stephen Tong
Mercy Hospital for Women
University of Melbourne
Tel: +613 8458 4380
Email: stong@unimelb.edu.au

Prof Susan Walker
Mercy Hospital for Women
University of Melbourne
Tel: +613 8458 4380
Email: spwalker@unimelb.edu.au

Data Safety and Monitoring Committee

Chair
Prof Anna David
Academic Obstetrician/ Maternal Fetal Medicine Specialist, University College, London
Email: a.david@ucl.ac.uk

Members
Prof Michael Stark
Academic neonatologist, Robinson Research Institute, University of Adelaide
Email: michael.stark@adelaide.edu.au

Prof Helmuth Reuter
Physician/ Pharmacologist, Stellenbosch University
Email: helmuthreuter@sun.ac.za

Declaration

This study will be conducted in compliance with the clinical study protocol (and amendments), International Council for Harmonisation (ICH) guidelines for current Good Clinical Practice (GCP), World Medical Association Declaration of Helsinki, and applicable regulatory requirements.

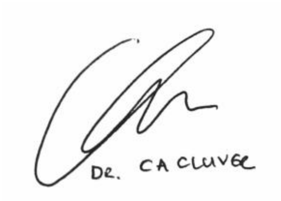

Signatory: Prof Catherine Cluver
Date: 6 June 2024

Contents

Investigator Contact Details 2

Trial Committees 4

Trial Steering Committee 4

Data Safety and Monitoring Committee 4

Summary 10

Abbreviations 13

Definitions 14

Protocol amendments 16

1. Background 19

1.1 Introduction 19

1.2 Pathogenesis of Preeclampsia 20

1.3 Treatment of Preeclampsia 22

Clinical trials investigating treatments for preeclampsia 23

1.4 Potential of DM199 to treat preeclampsia 24

Summary 28

2. DM199 drug information 29

2.1 Drug information 29

2.2 Preclinical data 29

Nonclinical studies 29

Animal models 30

2.3 Clinical trial data in humans 31

Summary of completed clinical studies 33

2.4 Contraindications 34

2.5 Adverse events arising in specific clinical trial cohorts 34

2.6 Drug interactions 35

3. Study Design 36

3.1 Overview 36

3.2 Part 1- Dose finding 36

Type of study 36

Site 36
Aim 36
Inclusion and exclusion criteria 36
Design 37
Dose rationale 38
Sample size 39
Outcomes 39
Pharmacokinetics 40
3.3 Part 2- safety, tolerability and pharmacokinetic study 41
Type of study 41
Site 41
Aim 41
Cohorts 41
Sample size 41
Inclusion and exclusion criteria for the three sub cohorts 42
Outcomes for the three sub cohorts 43
Dose 45
Pharmacokinetics 45
3.4 Study duration 46
4. Trial conduct 47
4.1 Identification and enrolment of participants 47
Identification 47
Consent and recruitment 47
Enrolment 47
4.2 Study procedures 47
Pre-dosing examinations 47
Administering medication 48
Special Investigations 48
Stopping the trial medication 50
4.3 Control of drug supply 50
4.4 Routine pregnancy care 50
4.5 Data collection 50

4.6 Sample collection and storage 50
4.7 Withdrawal from the study 51
5. Safety Monitoring and Procedures 52
5.1 Definitions 52
5.2 Evaluation of Adverse Events 53
Assessment of Severity 53
Assessment of Causality 56
5.3 Reporting of Adverse Events 56
5.4 Adverse Event Follow-up 56
5.5 Reasons to stop the trial 57
6. Data management 58
6.1 Data management 58
6.2 Case Report Forms 58
6.3 Source Documentation 58
6.4 Data processing 58
7. Statistical analysis and considerations 59
7.1 Study design 59
7.2 Sample Size Determination 59
7.3 Statistical Methods 59
7.4 Outcomes 59
Safety 59
Vital Signs 59
Clinical Laboratory 59
Pharmacokinetic and Pharmacodynamic parameters 60
8. Study Monitoring and Auditing 61
8.1 Monitoring and Source Document Verification 61
8.2 On-Site Audits/Inspections 61
9. Study Administration 62
9.1 Regulatory and Ethical Considerations 62
Protocol and Amendment Approvals 62
Protocol Deviation Management 62
9.2 Final Report 62

9.3 Timelines 62
9.4 Post trial access to medication 62
9.5 Publication of results 63
9.6 Role of study funders 63
10. Ethical aspects 64
10.1 Confidentiality 64
10.2 Record Retention 64
10.3 Compensation for participation 64
10.4 Risks and Benefits 64
Potential risks 64
Potential Benefits 65
Risk Minimization 66
11. Resources and strengths of the study 67
11.1 Strengths 67
11.2 Limitations of this study 67
References 68

Summary

Introduction

Preeclampsia and fetal growth restriction are leading causes of maternal and fetal morbidity and mortality in South Africa. Both result from poor placental function due to malperfusion. Preeclampsia is further characterised by inflammation and oxidative stress, leading to maternal endothelial dysfunction and hypertension. Hence, a drug that improves maternal vascular function including vasodilatation (and blood pressure reduction) may be a treatment for both conditions.

Tissue kallikrein (KLK1) is an endogenous enzyme that cleaves kininogen to produce active kinins, mainly bradykinin. Bradykinin is a potent natural vasodilator with pro-angiogenic and possible anti-oxidant and potentially anti-inflammatory effects. Specifically, bradykinin binds and activates the bradykinin 2 receptor located on blood vessel endothelium. Activation of these receptors increases nitric oxide and prostacyclin production, resulting in relaxation of the smooth muscle of blood vessels and consequently vasodilation. Bradykinin 2 receptor activation may also have other beneficial actions such as upregulating antioxidant defenses.

DM199 is a pharmaceutical formulation comprised of recombinant tissue kallikrein. It is a protein that is identical to KLK1, except for two amnio acids. Given the beneficial actions of KLK1, DM199 could be a therapeutic to treat preeclampsia. Furthermore, its vasodilatory properties might enhance blood perfusion to the placenta. If so, it could have merit in treating fetal growth restriction.

Preclinical studies (including animal toxicology studies) and clinical trials (non-pregnant population) have shown DM199 to be safe and well tolerated. Additionally, it is a protein meaning it is unlikely to cross the placenta and reach the fetus. These properties make DM199 an ideal candidate to evaluate as a possible treatment of preeclampsia and possibly fetal growth restriction.

This protocol describes an unblinded study to determine an effective dose of DM199 when given to women diagnosed with preeclampsia and/or fetal growth restriction and to evaluate safety

Objectives

- To determine an effective dose of DM199 in pregnant women diagnosed with preeclampsia
- To evaluate safety and efficacy of DM199 in preeclampsia and/or fetal growth restriction

Study design

**Study type**	Open-label, phase IB/IIA single centre study	
**Sample size**	Total of up to 132 pregnant women	
	Part 1	Ascending dose finding study including up to 42 women with preeclampsia complicated by severe hypertension who need delivery within 72 hours. Three women will be recruited in each sequential cohort
	Part 2	Safety and efficacy study with 30 women in each cohort using a dose from Part 1. The dose will be determined by Part 1<br>*2.1*: Women with preeclampsia with severe hypertension needing delivery with 72 hours<br>*2.2*: Women with preterm preeclampsia undergoing expectant management<br>*2.3*: Women with fetal growth restriction admitted for inpatient monitoring
**Inclusion criteria**	• Singleton pregnancy with a gestational age between 27+0 weeks and 42+0 weeks<br>• Diagnosis of preeclampsia and/or fetal growth restriction<br>• Pregnant women over the age of 18 who are able to give informed consent<br>(There are specific inclusion criteria for Part 1 and the three sub-cohorts in part 2)	
**Exclusion criteria**	• Hypotension -systolic blood pressure (BP) <90mmHg and/or diastolic BP <60mmHg and/or a mean BP <65mmHg<br>• Clinical infection like chorioamnionitis<br>• Cardiovascular disease e.g. mitral valve stenosis, significant arrythmia<br>• Vascular disease e.g. renal artery stenosis	
**Outcomes**	**Part 1: Dose finding study**	
	Primary	• Incidence of treatment emergent adverse events

		• Umbilical cord blood levels of DM199 after birth<br>• Acute change in maternal blood pressure from baseline assessed immediately after the completion of the infusion, at 30 minutes post-infusion and at 24 hours after the intravenous dose
	Secondary	• Maternal pharmacokinetic profile of DM199 in preeclampsia<br>• Change in maternal blood pressure from baseline to delivery<br>• Uterine contractions
	**Part 2.1: Preeclampsia requiring delivery within 72 hours**	
	Primary	• Change in maternal blood pressure from baseline assessed immediately after the completion of the infusion, at 30 minutes post-infusion and 24 hours after the initial dose<br>• Incidence of treatment emergent events<br>• Umbilical cord blood levels of DM199 after birth
	Secondary	• Uterine contractions<br>• Episodes of severe hypertension or hypotension after administration of DM199<br>• Use of other antihypertensive agents<br>• Changes in uterine and ophthalmic artery Doppler parameters
	**Part 2.2: Preterm preeclampsia undergoing expectant management**	
	Primary	• Prolongation of pregnancy (in days and hours)<br>• Change in 24-hour protein creatinine ratio one week after enrolment, compared to baseline values<br>• Need to increase or decrease other antihypertensive agents<br>• Incidence of treatment emergent adverse events<br>• Umbilical cord blood levels of DM199 after birth
	Secondary	• Change in maternal blood pressure from baseline<br>• Number of women reaching 34 weeks gestation<br>• Severe hypertension or hypotension<br>• Uterine contractions<br>• Changes in uterine artery, ophthalmic artery and fetal Doppler flow indices<br>• Neonatal length of stay at Tygerberg hospital and overall, in any hospital
	Part 2.3: Fetal growth restriction	
	Primary	• Changes in uterine artery and ophthalmic artery Doppler flow indices<br>• Changes in fetal Doppler parameters<br>• Birthweight centile<br>• Incidence of treatment emergent adverse events<br>• Umbilical cord blood levels of DM199 after birth
	Secondary	• Prolongation of gestation (measured from time of first dose to delivery)<br>• Fetal growth trajectory (if two ultrasounds measuring fetal growth are done during the pregnancy)<br>• Changes in maternal blood pressure<br>• Use of antihypertensive medication (if unmedicated at enrolment) or the need to increase or decrease other antihypertensive agents
**Recruitment**	Participants will be identified in the Obstetrics Department at Tygerberg Hospital. A member of the research team will discuss the trial with potential participants.	
**Informed consent**:	Potential participants will be given the information sheet, and all questions will be answered. If they would like to be included in the trial they will be asked to sign the informed consent document.	
**Study processes**	After consenting, all eligibility criteria will be checked again. The participant will then be assigned to a study arm. Her vital signs will be monitored, and a blood sample will be drawn. Once an initial examination has been performed, DM199 will be administered. A member of the research team will monitor the participant closely during the study until delivery when the treatment will cease. We will collect clinical information, including maternal, fetal and neonatal outcomes. Blood samples will be collected and investigations including cerebral autoregulation, Doppler ultrasound	

	studies and flow mediated blood vessel dilatation will be performed. Women will be followed up until 6 weeks after the delivery date.
**Post-trial access**	DM199 will be tested to treat pregnancy related complications. Women included in the trials will be treated until delivery. There will be no need for post-trial access as they would not be pregnant
**Timeline**	2 years from receiving ethical, hospital and SAHPRA approval
**Ethical considerations**	
For many years, pregnant women have been excluded from trials due to safety concerns. This has resulted in no new drugs being developed for pregnancy complications. Research studying new treatments are urgently needed. DM199 is an ideal candidate for preeclampsia and fetal growth restriction. It is a naturally occurring protein that decreases severe hypertension, improves endothelial dysfunction and is very likely too large to cross the placenta (proteins cannot diffuse across the lipid bilayer of cellular surfaces). Hence, not only is it ethical to evaluate the potential of DM199 to treat these life-threatening conditions, safety and efficacy trials are urgently needed.	
**Anticipated overall outcome**	
We aim to assess DM199's safety and tolerability in pregnancy. If safe and tolerable, with changes in maternal blood pressure, the early phase trials described in this protocol will justify further large, randomised trials assessing DM199 as a treatment for preeclampsia and fetal growth restriction.	
**Local social value**	
Preeclampsia and fetal growth restriction are leading causes of both maternal and fetal morbidity and mortality. Southern Africa in particular, carries one of the highest burdens of preeclampsia related morbidity and mortality. Unfortunately, the majority of preeclampsia research, particularly in the field of therapeutics, has been performed in high-income countries, who carry the lowest burden of preeclampsia morbidity. This does not benefit the majority of women who carry the largest burden of complications. It is very important that research is done in the women who would benefit the most from new therapies. Running this trial at Tygerberg Hospital is important to assess if it would be safe and efficient for women who suffer the most morbidity.	

Abbreviations

AE	Adverse event
BMI	Body mass index
DLT	Dose limiting toxicity includes all drug related ≥ Grade 3 AEs which may include but are not limited to allergic/hypersensitivity reactions, dermatological reactions, severe hypotension (BP≤ 90/60mmHg), angioedema and or bradycardia.
DM199	Pharmaceutical formulation of recombinant human tissue kallikrein-1
DMP	Data monitoring plan
DSMC	Data safety monitoring committee
FDA	United States Food and Drug Administration
GCP	Good clinical practice
GLP	Good laboratory practice
HELLP syndrome	Haemolysis, elevated liver enzymes and low platelet syndrome
Hrs	Hours
HREC	Health research ethics committee
ICH	International Council for Harmonisation of Technical Requirements for Pharmaceutical for Human Use
IV	Intravenous
KLK1	Tissue kallikrein
NOAEL	No observed adverse effect level' (NOAEL)
NO	Nitric oxide
SAE	Serious adverse event
SAHPRA	South African Health Products and Regulatory Authority
SC	Subcutaneous
PIGF	Placental growth factor
sFlt-1	Soluble fms-like tyrosine kinase-1
VEGF	Vascular endothelial Growth Factor

Definitions

The following definitions will be used in this trial

**Hypertension**	Hypertension in pregnancy is diagnosed when the systolic blood pressure is greater than or equal to 140 mmHg and/or a diastolic blood pressure greater than or equal to 90 mmHg on the average of at least two measurements, taken at least 15 minutes apart.
**Severe Hypertension**	Systolic blood pressure greater than or equal to 160 mmHg or a diastolic blood pressure greater than or equal to 110 mmHg.
**Hypotension**	Mean arterial blood pressure less than or equal to 65 mmHg
**Pre-existing (chronic) hypertension**	Hypertension that pre-dates the pregnancy or appears before 20 weeks gestation.
**Gestational Hypertension or pregnancy induced hypertension**	Hypertension that appears at or after 20 weeks of gestation.
**Significant proteinuria**	Proteinuria greater than or equal to 0.3 g/d in a complete 24-hour urine collection or a spot (random) urine sample with greater than or equal to 30 mg/mmol urinary protein creatinine ratio or 2+ or more proteinuria on urine dipstick testing.
**Preeclampsia**	New onset hypertension *plus* proteinuria and/or evidence of end organ dysfunction after 20 weeks' gestation.(1) Preeclampsia is a progressive disease of pregnancy involving multiple organ systems. The clinical definition has evolved over time from simply hypertension and proteinuria to a broader classification that recognises the complex multi-organ system involvement caused by the disease. For this trial we will still require hypertension with significant new onset proteinuria or an increased degree of proteinuria in those with underlying kidney disease as part of the diagnosis.
**Super-imposed preeclampsia**	Loss of blood pressure control in a women known to have hypertension on medication with new onset or worsening proteinuria or maternal end-organ dysfunction.
**Unclassified proteinuric hypertension**	Hypertension and proteinuria diagnosed in a patient who is seen for the first time after 20 weeks of gestation.
**Adverse event**	Any untoward medical occurrence associated with the use of a drug in humans, whether or not considered drug related
**Severe renal involvement**	Creatinine level greater or equal to 90µmol/L
**HELLP syndrome**	Haemolysis, elevated liver enzymes and low platelets (HELLP) syndrome defined as a platelet count less than 100 × 109/L, aspartate aminotransferase greater than 70 µ/L, and haemolysis as demonstrated by

	lactate dehydrogenase > 600 µ/L or haemolysis on a peripheral blood smear
**Eclampsia**	New onset of grand mal seizure activity and/or an unexplained coma during pregnancy with signs or symptoms of preeclampsia
**Pulmonary edema**	Oxygen saturation less than 92% with bibasal crepitations on auscultation and signs of pulmonary edema on chest X-ray
**Raised liver enzymes**	Liver transaminases (aspartate aminotransferase and/or alanine transferase) greater than or equal to 70IU/L

Protocol amendments

Date and version	Page	Description	Details
1 March 2025<br>Version 1.1	1	Date and version number updated	Version 1.1<br>Date: 1 March 2025
	4	Affiliation corrected	Changed to University College
	6	Contents page updated	
	10	Summary updated	Number of particpants included in part 1 increased from up to 30 to 42.<br>Total number of participants included increased from 120 to up to 132<br>Following text added to exclusion criteria<br><br>and/or a mean BP <65mmHg
	27	Correction of an editing error.	The following was deleted:<br><br>An active transport mechanism.
	37	A link to the protocol for managing hypertensive pregnancies at Tygerberg Hospital has been added.	The following text has been added:<br><br>The decision for the need for delivery will be made by the treating clinical team and is based on the Protocol for the management of Hypertensive Disorders in Pregnancy at Tygerberg Hospital (https://www.obstyger.co.za/Downloads/TBH_2019%20Hypertension%20in%20pregnancy.pdf).
	37-38	Definitions of dose-limiting toxicity have been clarified.	The following text has been added:<br>A DLT includes the following<br>• maternal hypotension (defined as grade 3 or higher adverse event requiring medical treatment or mean arterial blood pressure less than 65mmHg)<br>• allergic or hypersensitivity reaction (defined as grade 3 or higher adverse event requiring medical treatment)<br>• dermatological reaction (defined as grade 3 or higher adverse event requiring medical treatment)<br>• any other reaction that the investigators feel meets the criteria for a DLT (which includes adverse events graded as 3 or higher)

	39	Added 2 cohorts (<34 weeks and 34 weeks and over) of 6 women each to confirm. These cohorts will final dose identified in part 1 provides a therapeutic response, before moving to part 2	The following text has been added:<br>If a therapeutic dose of DM199 is discovered, we will then confirm that the dose is effective in 6 women with preeclampsia diagnosed before 34 (+0) weeks and 6 women diagnosed with preeclampsia from 34 (+0) weeks, and over.
	42	The inclusion and exclusion criteria have been refined.	The following text has been added under the specific inclusion criteria for cohort 2.3:<br><br>• No evidence of a fetal infection including syphilis, parvovirus or cytomegalovirus.<br>• The mother must not have a medical disorder preventing her from having normal food intake (e.g. hyperemesis gravidarum, Crohn's disease, ulcerative colitis, anorexia or bulimia).<br><br>We have added the following under the exclusion criteria for hypotenion<br><br>and or a mean arterial BP <65mmHg
	48	Details on fetal monitoring during medication administration have been added.	The following text has been added under the section administering:<br><br>The fetal condition will be continuously monitored while receiving the IV infusion and for at least an hour after the IV infusion with a cardiotocograph machine. An abnormal cardiotocograph will be diagnosed using the National Institute for Health and Care Excellence. Guideline on fetal monitoring in labour.
	48-49	Details on the ophthalmic artery Doppler examination have been added	The following text has been added:<br><br>The ophthalmic artery Doppler will be measured non-invasively with the patient in the supine position after resting for 10 minutes in the left lateral decubitus position. To perform the Doppler, a linear transducer (5–10MHz) will be positioned laterally across the upper eyelid of the pregnant woman's closed eyes. After identifying the maternal ophthalmic artery using color Doppler, the region medial to the optic nerve, approximately 15mm from the optic disc, will be examined. The following parameters will be standardized: insonation angle< 20°, pulse repetition frequency 125 Hz, and sample volume 2 mm. The Doppler parameters will be analyzed after obtaining at least three consecutive uniform waves. The following parameters will be obtained automatically: PI, RI, peak systolic velocity, second peak velocity, and end-diastolic velocity. The P2 peak systolic velocity (RPV) ratio will be calculated as P2/PSV.

	49	Breastmilk sampling has been added for pharmacokinetics	The following text has been added:<br>A sample of colostrum/breastmilk (up to 2ml) will be collected within 24 hours of birth for pharmacokinetic studies.
	49-50 Table 5	Table has been updated and timing for special investigations has been clarified	Opthalmic artery Doppler and colostrum/breastmilk sampling has been added to the table.<br><br>The following text has been added as footnotes to table 5 which summarises the trial schedule:<br><br>Endothelial dysfunction testing, cerebral autoregulation and opthalmic artery Doppler will only be performed in Part 1 once a therapeutic dose has been found.<br><br>We have added that a colostrum/breastmilk samples will be collected within 24 hours of delivery.
	50	Added stopping criteria	The following text has been added<br><br>**Stopping the trial medication**<br>If urgent delivery is required for maternal or fetal indications, the trial medication infusion will immediately be stopped.
	55-56	Grading classifications for adverse events have been added	The following text has been added under adverse events:<br><br>Adverse events not listed above in the tables will be graded using the latest available version of Common Terminology Criteria for Adverse Events (CTAE) (https://ctep.cancer.gov/protocoldevelopment/electronic_applications/docs/ctcae_v5_quick_reference_5x7.pdf).<br><br>Maternal and fetal adverse events will be graded using the Maternal and Fetal Adverse Event Terminology (MFAET) v1.1, 2022 or later versions (https://www.ucl.ac.uk/womens-health/sites/womens_health/files/mfaet_version_1.1_with_instructions_for_use.pdf).<br><br>Neonatal adverse events will be graded using the Neonatal Adverse Event Severity Scale (NAESS)

1. Background

1.1 Introduction

Preeclampsia is a life-endangering pregnancy complication

Preeclampsia is an unwelcome complication affecting 5-7% of all pregnancies.(2) It is one of the two leading causes of maternal death during pregnancy. For every maternal death related to preeclampsia, another 50 to 100 women suffer severe health injuries.(3) Around the world, there are estimated to be around 1.6 million cases of preeclampsia with severe features every year.

Preeclampsia is a pregnancy specific disorder that presents with hypertension and multi-organ injury in the second half of pregnancy. A hallmark of preeclampsia is severe maternal vascular dysfunction, where damage to the mother's blood vessels leads to hypertension and injury to many vital organs. The mother is at risk of developing seizures (eclampsia), cerebral injury like infarctions or intracranial haemorrhage, renal injury or failure, hepatic rupture and pulmonary edema. She may also develop haematological complications which include haemolysis, elevated liver enzymes and low platelet (HELLP) syndrome and disseminated intravascular coagulation. This puts her at a high risk of haemorrhage. Preeclampsia can also cause a placental abruption, where the placenta prematurely separates from the uterine wall due to bleeding resulting in catastrophic consequences for both the mother and unborn child.

Preeclampsia is both a placental and maternal disease. In early pregnancy the placenta fails to properly implant in the inner lining of the uterus. This may result in co-existing fetal growth restriction where the fetus fails to reach its genetically pre-determined growth potential. Fetuses with fetal growth restriction are at increased risk of adverse perinatal outcomes including stillbirth.

Preeclampsia is common

Preeclampsia complicates about 5% of all pregnancies and is estimated to cause at least 42,000 maternal deaths every year.(4,5) Worldwide, it is estimated that over 90% of deaths caused by preeclampsia occur in low and middle-income countries. Globally, preeclampsia disproportionately affects minority populations and those living in low and low middle-income countries. In South Africa, hypertensive disorders of pregnancy are responsible for 14% of all maternal deaths.(6)

Preeclampsia has no treatment apart from delivery

There are certain medications given to women with preeclampsia: drugs to reduce hypertension or an infusion of magnesium sulphate to reduce the risk of eclampsia. However, these medications only control the late end-organ consequences of preeclampsia. Critically, there are no drugs that slow the underlying disease progression (such as improve placental health or quench the blood vessel injury).

Because there are no drugs to treat preeclampsia, delivery is the only definitive treatment. Birth of the fetus arrests the disease because the placenta is removed – the source of the anti-angiogenic factors responsible for the vascular and end-organ damage. Even after delivery, it may take weeks for a woman to recover from the acute end-organ complications of preeclampsia.

Preeclampsia has lifelong implications

Having had preeclampsia leaves a lifelong legacy of an increased risk of chronic illnesses for the mother, especially cardiovascular health risk. Over the remainder of her life she has an increased risk of developing chronic hypertension, a 2-4 fold increased risk of stroke, heart and renal failure, and death from cardiovascular disease.(2,7) Frequently the baby is born growth restricted, premature, or both: these synergise to incur lifelong adverse health effects for the newborn.(2)

1.2 Pathogenesis of Preeclampsia

Preeclampsia arises in two stages: the first is placental disease which is followed by maternal vascular injury.

Stage 1: Placental disease

In normal early pregnancy, the placenta actively remodels the maternal vasculature inside the uterus. Columns of placental cells enter spiral arterioles in the uterus (maternal vessels) and strip them of their muscular walls, rendering them unable to contract. The result of spiral arteriole remodelling is a low-pressure, high-capacity system which is ideal for the transfer of nutrients and oxygen to the growing fetus.(8–11)

In preterm preeclampsia and fetal growth restriction, uterine vascular remodelling goes awry.(12,13) The maternal spiral arterioles retain some ability to contract, resulting in a high pressure, turbulent blood flow..(13–15) This injures the placental cells and causes cellular stress of the surface cellular layer of the placenta, the syncytiotrophoblast.

As pregnancy continues into the second trimester, the diseased preeclamptic placenta secretes elevated amounts of anti-angiogenic factors into the maternal circulation that cause the maternal vascular injury seen in stage two of preeclampsia. There are many candidate factors secreted in excess by the preeclamptic placenta that may contribute to blood vessel dysfunction.(16) A likely central driver is an anti-angiogenic protein called soluble fms-like tyrosine kinase-1, or sFlt-1.(10,16,17) sFlt-1 binds to the pro-angiogenic factor VEGF-1 (vascular endothelial growth factor-1) and renders it inactive. VEGF signalling on blood vessels is necessary to maintain a pro-angiogenic state. Neutralized by sFlt1, VEGF-1 can no longer bind to its specific receptors studded along blood vessels to maintain healthy blood vessel homeostasis.(17)

In addition to sFlt-1 are many other factors secreted in excess by the diseased placenta into the maternal circulation to exacerbate the maternal vascular damage. These include pro-inflammatory cytokines, exosomes, and other anti-angiogenic molecules.(8,18–20)

Stage 2: Endothelial dysfunction and maternal vascular disease

In the second stage of the pathogenesis of preeclampsia, the placenta releases these anti-angiogenic factors into the maternal circulation.(17) This results in vasoconstriction leading to hypertension and damage to maternal end-organs.

There are two main layers of arterial blood vessels. The inner lining is a single layer of endothelial cells that are linked together to form a watertight junction. Below the endothelial layer are vascular smooth muscle cells made up of multiple layers.

Figure 1: Pathogenesis of preeclampsia

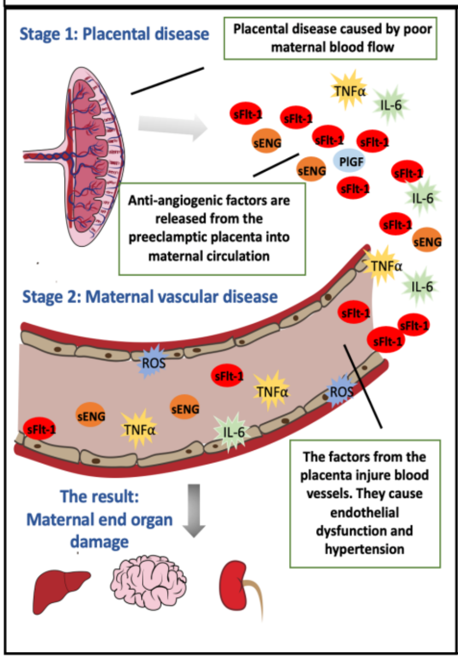

Stage 1: placental disease. Poor remodelling of the local maternal vascular injures the placenta and causes 'syncytiotrophoblast stress' (see text). The unhealthy syncytiotrophoblast releases a raft of anti-blood vessel (anti-angiogenic) factors into the maternal circulation. These include **anti-angiogenic proteins** sFlt1 and sEng, **oxidative stress factors** (ROS) and **proinflammatory cytokines** (such as TNF-a and IL-6)

Stage 2: maternal vessel disease and endothelial dysfunction. There is a maternal vascular injury caused by the anti-angiogenic factors released from the preeclamptic placenta. This vascular injury causes **endothelial dysfunction, hypertension** and **injury to many of the mother's organs.**

Brown structures in the blood vessels depict endothelial cells

sFlt1 – Soluble fms like tyrosine kinase
sEng – soluble endoglin
ROS – reactive oxygen species
TNF a – Tumour necrosis factor alpha
IL 6 – Interleukin 6

The role of the vascular smooth muscle layer is to contract (narrowing the diameter of the blood vessel) and increase the blood pressure or relax (widens the diameter of the blood vessel), reducing blood pressures allowing a greater volume of blood to flow through. Whether vascular smooth muscle cells contract is dictated by signals received from the adjacent endothelial cells.

Endothelial cells are the master regulators of blood vessels and take the lead in coordinating blood pressure and general vascular health. They receive messages from proteins and molecules in the bloodstream and act on them. They can send messages into the bloodstream to be relayed to distant blood vessels. They may also relay local information in the other direction, to the vascular smooth muscle cells lying just beneath.

Specifically, endothelial cells:

1. **Receive signals from molecules present in the circulation**. These molecules will usually lock onto its specific receptor on the cell surface of endothelial cells. The receptors will relay signals inside the cell to then issue further commands.

Example of signals include vascular endothelial Growth Factor (VEGF) or placental growth factor (PIGF) (both promote vessel relaxation), or bradykinin (promotes vascular relaxation and a fall in blood pressure) (See figure 2, Endothelial cell signalling). Angiotensin II binds to the angiotensin receptor to promote vascular constriction (increasing blood pressure).

2. **Send signals locally to the underlying vascular smooth muscle:** Endothelial cells can release molecules that instruct vascular smooth muscles to either dilate or constrict. Three important molecules released by the endothelium are nitric oxide (NO), prostacyclin and endothelium derived hyperpolarizing factor (see figure labelled Endothelial cell signalling). They are the main players promoting local vascular relaxation and reducing blood pressure. Endothelin-1 is another vasoactive molecule, a peptide that is one of the most potent, naturally occurring vasoconstrictors (causing hypertension). Some of these molecules, such as endothelin-1 and nitric oxide may also be released into the bloodstream where they presumably travel to more distant blood vessels to exert their effects.

3. **Send signals into the bloodstream:** Endothelial cells can release specific molecules into the blood that travel distantly to signal and target other endothelial cells. Examples are sFlt1 and cytokines which cause a pro-inflammatory state leading to endothelial dysfunction.

Figure 2: Endothelial cell signalling

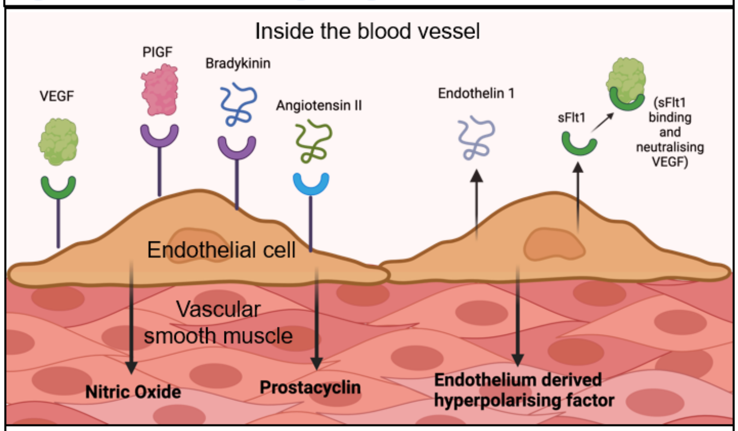

Endothelial cells are master controllers of blood vessels. Endothelial cells can:

1) **Receive signals from molecules in the blood:** Such as VEGF, PIGF, Bradykinin and angiotensin II. These molecules latch onto specific receptors on the surface of endothelial cells
2) **Send signals into the blood:** Such as endothelin 1 and sFlt1 as well as others.
3) **Send signals locally to the underlying smooth muscle:** Such as nitric oxide, prostacyclin and endothelium derived hyperpolarising factor.

VEGF – vascular endothelial Growth Factor PIGF – Placental growth factor
sFlt-1 – soluble fms like tyrosine kinase

In preeclampsia, the placental factors released into the maternal circulation (stage 1) cause endothelial dysfunction (stage 2). They switch on molecular cascades causing endothelial cells to issue signals for blood vessels to contract. This leads to hypertension. In addition, vasoconstriction reduces blood flow to many vital organs.

The elevated levels of sFlt1 from the placenta reduces VEGF signalling. The preeclamptic placenta also secretes less PIGF (VEGF and PIGF signalling is desirable as they cause vasodilation).(16)

The endothelial dysfunction also reduces production and release of nitric oxide, and prostacyclin into the vascular smooth muscle, resulting in vasoconstriction.

The endothelial dysfunction is driven by a pro-inflammatory state (where cytokine molecules signal to the endothelial cells, contributing to the dysfunction) and oxidative stress in the cells. These processes cause the endothelial cells to become dysfunctional, and this contributes to the release of signalling molecules to the vascular system resulting in a pro-hypertensive state.

Thus, endothelial dysfunction causes widespread hypertension and vessel injury. This reduces blood supply to many of the mothers' vital organs and increased passage of fluid out of blood vessels. The net result is hypertension, edema, maternal vascular disease and maternal organ injury.

The severity of preeclampsia varies greatly. It can be mild, where laboratory tests reveal maternal organs to be mildly injured and are expected to promptly recover after the placenta is removed. However, preeclampsia can be very dangerous - where the organ injury is so severe it threatens the life of the mother and unborn baby.

1.3 Treatment of Preeclampsia

A drug that can slow disease progression for preeclampsia would be a major breakthrough. It could save many lives and reduce healthcare costs. Unfortunately, no such drug exists.

Without a specific treatment, the approach to clinical management when preeclampsia is diagnosed is to 'watch and wait' and to 'time birth'. We closely monitor the degree of maternal organ damage and if severe injury is apparent, we deliver the baby.

A drug that could quench the underlying pathology of preeclampsia could ameliorate the severe injury to maternal organs. Such a drug could prove useful for all types of preeclampsia:

1. **Early preterm preeclampsia:** It would be particularly useful in cases of preterm preeclampsia**:** if the drug can reduce the disease impact, the pregnancy could be allowed to safely continue. Instead of immediately delivering the baby preterm, it could be safely left in the uterus to further develop and

reach a more mature gestational age. This could reduce immediate health care costs in the way of avoiding neonatal intensive care admission and present the baby with better prospects of lifelong health.

2. **Preeclampsia at late preterm/ term gestation:** For preeclampsia occurring at late preterm gestation (diagnosed when the pregnancy has reached at least 34 weeks of pregnancy) we would usually proceed with planning birth soon. Even for these cases, a safe drug that tackles the underlying disease pathology would be useful. This is because by the time preeclampsia is diagnosed, there is often already evidence of severe organ injury(21) (preeclampsia with severe features, using criteria published by the American College of Obstetricians and Gynecologists).(22) Hence, the drug could quell the disease long enough to buy time – around 24-72 hours - for clinicians to safely birth the baby. It could offer safer outcomes for the mother.
3. **Postpartum preeclampsia:** The damage caused by preeclampsia takes time to resolve. After birth, mothers may still suffer severe complications of preeclampsia. These complications include very high blood pressures, eclampsia, pulmonary edema and heart failure. Current treatments can improve the symptoms, but better treatments are needed that ameliorate preeclampsia.
4. **Fetal growth restriction:** Preeclampsia is often accompanied by fetal growth restriction which is a result of uterine artery vasoconstriction and placental dysfunction. If a treatment could reverse the vasoconstriction, it may improve fetal growth and development.

Clinical trials investigating treatments for preeclampsia

There have been very few randomised clinical trials of potential therapies to treat preeclampsia. Nearly all have reported negative findings, suggesting the investigational drug was not effective.

The general approach to randomised trials testing investigational agents to treat preeclampsia is to administer the drug to women with preterm preeclampsia. The primary outcome for most trials has been to assess how long preeclamptic women were able to remain pregnant before birth was required, because maternal organ injury became too severe (or the fetal condition deteriorated). Hence, a longer period between randomisation and birth would suggest the drug was able to quench the disease process and allow the pregnancy to safely progress for longer (and for the baby to stay longer in the uterus to develop and mature). At preterm gestations (such as 24-32 weeks gestation), even gains of around 5-7 days are likely to translate into significant health improvements for the newborn and reduce the risk of death, or permanent chronic illnesses.

A number of drugs have been investigated.

Sildenafil: A trial of oral sildenafil (50mg, three times a day) given to women with preterm preeclampsia significantly lengthened the pregnancy by 4 days. Unfortunately, since then, a large trial evaluating sildenafil to treat fetal growth restriction was abruptly stopped. It raised concerns that sildenafil was crossing the placenta into the fetal circulation, causing fetal lung injury (neonatal pulmonary hypertension) and neonatal loss.(23,24) Hence, sildenafil cannot be considered further.

Metformin: We published a randomized trial in *British Medical Journal* showing oral metformin may prolong gestation by a week in women admitted for expectant management.(25) Metformin is a drug used to treat diabetes, including diabetes in pregnancy. We embarked on this trial in light of our prior laboratory experiments which demonstrated metformin may have actions that improve endothelial function and

dilate blood vessels.(15) We are now undertaking randomized validation trials at Tygerberg Hospital, Cape Town and a multicentre randomized validation study in Sweden.

Other agents that did not work in randomised trials: Randomised trials of investigational agents that have **reported a negative outcome** include intravenous **antithrombin** III (meant to dampen the immune system but a US multi-centre trial failed to find benefit,(26) an unpublished Japanese trial also did not find benefit [drug name KW-3357])(27); **pravastatin**(28) (a cholesterol lowering drug postulated to have actions that counter preeclampsia(29,30)), and a randomised trial we did that tested 40 mg of **oral esomeprazole**(31) (drug used to treat gastric reflux, also postulated to have molecular actions that could counter preeclampsia(32)).

Comanche Biopharma is a US company that is examining a molecular therapy (comanchebiopharma.com). Based on preclinical studies,(33) they are testing whether administration of a drug made of short interfering RNA) – (**siRNAs)** – can enter the placenta and reduce the production and release of **sFlt1**, one of the main drivers causing the endothelial dysfunction. This concept is early stage: to date, they have not published results of early phase trials.

1.4 Potential of DM199 to treat preeclampsia

The ideal drug to treat preeclampsia may be one that **1)** is highly efficacious at reducing blood pressure, **2)** increases blood flow to the placenta to reduce placental disease, **3)** reduces endothelial injury, 4**)** does not cross through into the placenta and **5)** has already been shown to be safe when administered to humans in a non-pregnant population.

DM 199: a promising candidate treatment for preeclampsia

DM 199 is a manufactured version of Kallikrein-1 (or KLK1) but with small modifications that increases its stability. It has **potent blood pressure lowering effects** and does this by switching on existing natural molecular machinery within cells. It can be given as an intravenous dose (short acting), or as a subcutaneous injection (longer acting).

DM 199 is protein drug version of a natural enzyme Kallikrein-1: a key player in blood pressure reduction.

Kallikrein-1 is continually made and secreted from endothelial cells, but also likely released from other tissues, such as the kidneys, pancreas and lungs. **DM199 is a recombinant (synthetic) form of Kallikrein-1.** The main role of kallikrein-1 is to cleave inactive kininogens to make active bradykinin or lys-bradykinin. Bradykinin or Lys-bradykinin are cell signalling molecules that powerfully reduce blood pressure (See figure 3, Kallikrein 1 and DM 199 signalling).

Lys-bradykinin is a short 10 amino acid peptide that binds to and activates bradykinin 2 receptors that are studded on the surface of endothelial cells. Once activated, bradykinin 2 receptor triggers molecular circuitry within the endothelial cells that upregulates the production and release of two potent molecules that relax blood vessels and reduce blood pressure: **nitric oxide** and **prostacyclin**.

Nitric oxide and prostacyclin diffuse into the underlying vascular smooth muscle and causes them to relax. This reduced vascular contraction dilates the blood vessel.(34) Nitric oxide and prostacyclin are co-released from endothelial cells and believed to work in synergy. In addition, there is evidence that activation of the bradykinin 2 receptor also promotes release of a third vasodilating molecule, **endothelium derived hyperpolarizing factor**.(35) This also signals to the underlying smooth muscle and causes it to relax, further dilating blood vessels.

Hence, DM199 switches on existing molecular circuits to promote significant relaxation of blood vessels and a reduction in blood pressure. It is an excellent drug candidate to reduce the hypertension that is a consistent feature in preeclampsia.

When giving DM 199 to humans, there is a risk of causing hypotension if it is also taken with an ACE inhibitor. This is because ACE degrades bradykinin/lys-bradykinin and inactivates it. The inactivation of the ACE enzyme means prolonged bradykinin 2 receptor activation. Fortunately, ACE inhibitors are contra-indicated during pregnancy.

In addition to lowering blood pressure in the mother, DM199 has potential actions to counter preeclampsia: increase blood supply to the uterus to rescue the placental disease and reduce the endothelial dysfunction (reduce endothelial cellular dysfunction).

Figure 3: Kallikrein 1 and DM199 signalling

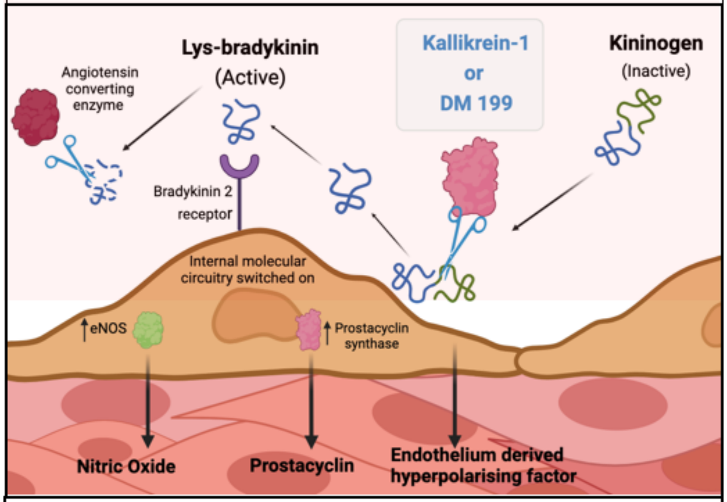

Kallikrein-1 and DM 199 make lys-bradykinin, a potent blood pressure lowering molecule

Kallikrein-1 or **DM 199** (drug version of Kallikrein-1) cleave inactive kininogens to make active **lys-bradykinin.** This happens in the bloodstream.

Lys-bradykinin then activates the **bradykinin 2 receptor** on the surface of endothelial cells. This switches on internal molecular circuitry which results in the production and secretion of the three main molecules that relax vascular smooth muscle cells and reduce blood pressure.

Activation of the bradykinin 2 receptor increases **endothelial nitric oxide synthase (eNOS)** which makes **nitric oxide**, and it increases the enzyme **prostacyclin synthase** which makes **prostacyclin.**

In addition, activation of the bradykinin 2 receptor may reduce inflammation (not shown in this diagram)

DM 199 may mitigate the placental disease - stage one of preeclampsia

It is plausible that DM 199 may not only act to protect the maternal blood vessels but could also reduce placental dysfunction – the origin of the anti-angiogenic factors causing widespread vascular damage.

The uterine arteries are the two main blood vessels supplying maternal blood to the uterus, providing oxygen and nutrients to the placenta and baby. The **uterine arteries narrow when there is preeclampsia** (this narrowing is readily detected using ultrasound where resistance to blood flow increases, measured on Doppler ultrasound as an increase in the 'pulsatility index'). This likely occurs because of the inadequate uterine vascular (spiral arteriole) remodelling during early pregnancy (discussed above) – increasing blood flow resistance.

Nitric oxide in the uterine arteries that results from DM 199 administration could result in uterine artery dilation. Hence, if DM199 can dilate uterine arteries (and also open constricted vessels immediately downstream such as the arcuate arteries and the spiral arterioles in the uterus), it could increase oxygenation to the placenta and fetus, reducing the placental dysfunction. This might reduce the placental injury and reduce the release of anti-angiogenic factors from the placenta, opening a second front to combat preeclampsia and fetal growth restriction.

DM 199 may have potential to treat fetal growth restriction

If placental rescue happens via dilation of the uterine arteries, there may be direct fetal benefits. Placental disease and low oxygenation restrict fetal growth (causing fetal growth restriction). Indeed, due to their shared origins as placental diseases, fetal growth restriction and preeclampsia often co-exist.

If we show in our planned preeclampsia trials that using DM 199 promotes uterine artery dilatation on ultrasound, it may mean placental and fetal rescue may be occurring. If so, DM 199 may have merit as a **treatment for fetal growth restriction**, in the absence of preeclampsia. This a potentially important breakthrough because fetal growth restriction is the leading cause of stillbirth (worldwide, 3 million babies are lost to stillbirth).

DM 199 may reduce endothelial cell dysfunction

It was noted previously that with preeclampsia, the endothelial cells – master controllers of blood vessels - are stressed (there is endothelial dysfunction). Activation of the bradykinin 2 receptor caused by DM199 may have beneficial actions that mitigate the endothelial dysfunction and quench vascular injury beyond just blood pressure control. For instance:

1. Activation of the bradykinin 2 receptor facilitates **signalling of VEGF molecule**. VEGF is the important **pro-angiogenic molecule** contributing to blood vessel health and the creation of new blood vessels. The activation of the bradykinin 2 receptor facilitates VEGF signalling two ways. First, it relays a signal to increase activation of the VEGF 2 receptor itself that sits on the cell membrane.(36) Secondly, signals are relayed into the nucleus to produce more VEGF protein as well as its main receptor (VEGF receptor 2).(37) There is a large body of literature showing tissue kallikrein (the natural version of DM199) is centrally involved in angiogenesis where VEGF is likely to play an important role in this new vessel creation.(37,38)
2. The downstream intracellular molecules switched on by the bradykinin receptor 2 may **reduce oxidative stress**.(39,40) Reducing oxidative stress would be advantageous as preeclampsia is associated with increased placental and systemic oxidative stress.
3. The downstream intracellular molecules switched on by the bradykinin receptor 2 may **reduce inflammation.** For example, nitric oxide does not just dilate blood vessels but also has inflammatory dampening actions.(41)
4. Stimulation of the bradykinin 2 receptor also increases insulin sensitivity, glucose uptake(42) and glycogen synthesis, reducing levels of glucose in the bloodstream. High blood glucose can injure endothelial cells. Reducing blood glucose levels may further reduce endothelial dysfunction (the preeclampsia risk is higher among women with diabetes during pregnancy).

All these actions to reduce endothelial dysfunction, a hallmark of preeclampsia, may help reduce disease severity (over and above simply lowering the blood pressure).

DM 199 has been shown to be safe in humans and decreases blood pressure.

Early phase first in human trials of DM 199 have shown it to be safe (see section 5). DM199 has and is being tested for chronic renal disease and stroke (see section below on DM199). Furthermore, that DM199 causes consistent falls in blood pressure in humans is reassuring. This is important 'proof of principle' data that DM199 is indeed active in humans and likely to be switching on the natural molecular circuitry, as hypothesised. It seems extremely likely it can drop blood pressure for women with preeclampsia.

Most drugs currently used to treat pregnancy conditions are also drug versions of natural molecules.

While DM 199 is a very novel approach to treat preeclampsia, it is notable most drugs used to treat obstetric conditions are drug versions of natural molecules: these drugs commandeer the body's natural molecular machinery to exert beneficial effects. For instance, natural progesterone is given in a vaginal

pessary to reduce the risk of preterm birth,(43) various types of prostaglandins (not prostacyclin) are given to prime the cervix in preparation for labour(44) and synthetic oxytocin (a short peptide released from the pituitary gland) is given to switch on active labour.

Hence, the concept of giving a drug that is an analogue of a compound that naturally occurs is not radical.

Circulating levels of Kallikrein-1, the natural form of DM199, may be reduced in preeclampsia.

A Chinese research group reported that blood levels of kallikrein-1 in 51 women with preeclampsia were around half of levels seen in 45 pregnant women without preeclampsia. The reduction was even more acute in women with severe preeclampsia.(45) Furthermore, an older study that studied 198 women reported kallikrein-1 levels in urine among women with preeclampsia were half levels seen in those free of the disease.(46)

DM 199 is likely to be safe in pregnancy as it is too big to cross the placenta

The majority of current drugs cross the placenta. Most drugs are small molecule drugs and readily diffuse across the cell membrane of the placenta to enter the fetal circulation. While most won't cause harm to the fetus (the fetus has a liver and kidneys capable of metabolising drugs to protect itself), some might.

Drugs that are large proteins do *not* cross the placenta as they are too big to diffuse through the cell surface. This makes protein drugs ideal for treating maternal conditions. Currently, there are very few protein drugs on the market.

DM 199 is a protein drug. Thus, using DM199 to treat preeclampsia is attractive because, as a protein of around 268 amino acids (the size of kallikrein-1), it is too big to passively diffuse across the placenta into the fetal circulation. This means it may be less likely to cause untoward effects on the fetus.

Outline of how preeclampsia evolves and how DM199 may work

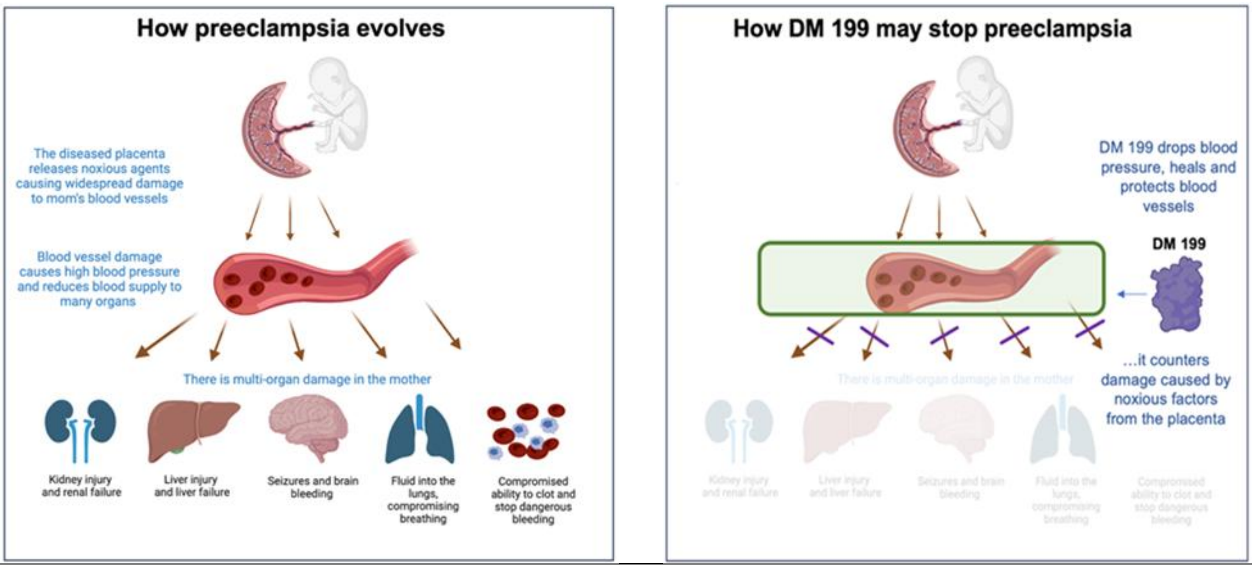

Summary

DM199 is a promising new treatment for preeclampsia for the following reasons

1. Reduces blood pressure, a hallmark of preeclampsia
2. May reduce endothelial dysfunction and maternal blood vessel injury
3. May rescue the placental disease
4. Safe in animal and human studies
5. DM199 is too big to cross the placenta
6. Animal reproduction toxicities studies in pregnant animals suggesting there is no harm to the offspring (see section 2).
7. In a pregnant population, patients with preeclampsia may derive most benefit from DM199, justifying the cautious dose-finding of DM199

DM199 may also be a treatment for fetal growth restriction

8. The placental rescue (if the uterine arteries dilate) may mean DM199 can increase placental perfusion which will increase oxygen and nutrient delivery

For these reasons, we will propose undertaking the phase I/II clinical trial outlined in this protocol.

2. DM199 drug information

2.1 Drug information

Drug substance

DM199 is a pharmaceutical formulation comprised of highly purified recombinant human tissue kallikrein-1. DM199 is expressed from a suspension culture of Chinese hamster ovary cells transfected with a gene encoding the full length of pre-pro-protein for human tissue kallikrein-1.

DM199 has a molecular weight of 26.4 kilodalton (excluding post-translational modifications) and a theoretical isoelectric point of 4.7. DM199 is the exact sequence as KLK1 (a protein with 238 amino acids) except it has amino acid substitutions, at positions 145 and 188 to increase stability. These substitutions differentiate it from naturally occurring human tissue kallikrein-1.

DM199 consists of two glycoforms in equal proportions. The first glycoform has two amnio acids which are glycosylated (have saccharides attached). The second form has three amino acid sites which are glycosylated.

Drug product

DM199 is presented as a sterile, clear, colourless, preservative-free solution in 2ml clear sealed sterile glass vials. It is supplied at a concentration of 500 µg/mL in 10 nM Sodium Phosphate, 150 mM sodium chloride, at a pH of 7.2 (Formulation Buffer). The excipients used in DM199 solution are compendial grade and are commonly used in parenteral formulation.

Active substance

DM199

Strength

500 µg/mL in a 2 mL glass vial, 1 mL fill volume

Storage and stability

DM199 storage is between -10°C to -25°C. Stability of the drug has been extensively tested after storage at 2°C to 8°C and it remains pyrogen free, sterile and fully active for more than 30 months at these temperatures.

Administration and dosage

DM199 can be administered intravenously (IV) or subcutaneously (SC). When DM199 is administered IV, a polyvinyl chloride (PVC) Baxter VIAFLEX® 50 mL infusion bag and PVC tubing must be utilized to prevent adhesion of the drug to the walls of the bags or the tubing.

2.2 Preclinical data

Nonclinical studies

The ability of DM199 to cleave low molecular weight kininogen and increase bradykinin has been compared to human urinary-derived KLK1 in laboratory studies. DM199 showed similar levels of enzymatic activity as

human urinary-derived KLK1. Increasing levels of DM199 generated increased levels of bradykinin (See DM199 investigator brochure for more details).

Animal models

Toxicology studies of DM199

DM199 has been shown to be safe in multiple animal models. These include mice, rats and monkeys. Toxicology studies conducted in accordance with Good Laboratory Practice (GLP) regulations demonstrated that DM199 is safe and well tolerated at doses 20-fold higher than expected human therapeutic doses. These studies included once daily subcutaneous administration for 28 days to Sprague-dawley rats and cynomolgus monkeys. In these 28-day studies, the no-observed-adverse-effect level (NOAEL) was 3600 µg/kg/day in rats and 1440 µg/kg/day in cynomolgus monkeys. In addition, a 6-month chronic toxicity and toxicokinetic study in monkeys also with once daily subcutaneous administration was completed. Like the 28-day studies, there was no effect of treatment with DM199 at doses up to 1,000 µg/kg/day (the NOAEL). The doses proposed in this study are well below doses that produced no adverse effects in either rats or monkeys. Further details on the toxicology studies can be found in the investigator brochure.

Reproduction toxicology studies of DM199

A full program of studies evaluating the potential effect of DM199 on reproduction and development in two animal species has been completed (see investigator brochure). These studies are required by the United States Food and Drug Administration (FDA) for investigating therapeutics in pregnant women, and the relative dosages of DM199 used in these animal studies are significantly higher than the proportionate dosing planned for humans in this proposed study. In total, 6 animal studies were completed, including a good laboratory practice (GLP) -compliant fertility study in rats, both non-GLP dose-range-finding and GLP definitive embryofetal development studies in rats and rabbits and a GLP peri-postnatal study in rats. These studies evaluate the potential of the drug to have an effect the 3 phases of reproduction, Segment I - effects on fertility, Segment II - effects on development of the embryo and fetus, and Segment III - effects on the fetus through sexual maturation of the offspring of treated mothers. The evaluations were conducted in accordance with Guidance promulgated in the International Council for Harmonisation of Technical Requirements for Pharmaceutical for Human Use (ICH) S5(R3), S6(R1) and M3(R2) and augment the safety data observed in over 250 non-pregnant human subjects treated with DM199.

Rats

There was no adverse effect of DM199 on male or female reproductive performance in the fertility study (Segment I) and the NOAEL in both males and females was the highest dose tested, 3000 µg/kg/day. Similarly, in the definitive rat embryo-fetal development study (Segment II), there was no maternal effect and no effects on embryo-fetal development at the highest dose evaluated resulting in a NOAEL of 3,690 µg/kg/day. Finally, in the definitive GLP peri-postnatal study in rats (Segment III) no maternal effects, no fetal effects and no effects on developing offspring occurred in rats administered DM199 at doses up to 3,000 µg/kg/day, the NOAEL.

Hence, all reproductive toxicology studies of DM199 in rats did not raise concerns.

Rabbits

In a second species of animal there was a severe immunogenic reaction in both non-pregnant and pregnant rabbits that resulted in mortality. The strong immunogenic response in rabbits occurred within 1 – 3 days of initiating treatment. DM199 is a humanized protein, and in some rabbits their immune systems mounted

a defense against DM199 as a perceived foreign antigen. The immune response was not related to the dose level, for example, similar reactions were seen in animals tested at low doses and at high doses. This, and the fact the reactions were observed in both pregnant and non-pregnant animals, strongly suggests that DM199 was not tolerated well by rabbits due to a cross-species reaction. Significantly, no immune reactions to DM199 have been observed in human subjects, including in a cohort of 82 patients treated continuously for 95 days, underscoring its tolerance in humans as opposed to the cross-species sensitivity seen in rabbits. The studies we plan to conduct have a maximum treatment duration of 56 days, and most patients will be treated for less than 14 days.

The increased sensitivity of rabbits to DM199 is not unique; increased sensitivity of rabbits to other human proteins has previously been reported.(47) Indeed, rabbits have historically been used to generate both monoclonal and polyclonal antibodies specifically because they have a strong immune response to antigens.(48)

Importantly, there was no evidence of teratogenicity associated with DM199, that is, among the fetuses from mothers that survived to the end of the study, there was no evidence of malformations such as birth defects. The significant maternal toxicity and lack of any observed teratogenicity suggests DM199 does not cross the placental barrier and reach the fetus. In general, large molecular weight proteins like DM199 do not (with few exceptions for antibodies) cross the placental barrier.

Despite the absence of a NOAEL for maternal or embryo-fetal development in rabbits, we conclude the embryo-fetal development toxicology studies in rats and rabbits are sufficient to assess the potential for any unexpected adverse effects on fetal development. This conclusion is drawn from the fact that the observed adverse effects were tied to significant maternal toxicity, with no evidence of fetal teratogenicity. In addition, there were no effects on fertility, fetal development or maturation of offspring from treated mothers. This understanding reinforces our confidence in the safety profile of the substance, given the notable physiological differences between rabbits and humans and the absence of any immune reaction in human subjects treated with DM199.

2.3 Clinical trial data in humans

DM199 has been administered to more than 280 human subjects across 7 trials. Six trials are completed, and one is ongoing. Studies include healthy volunteers, subjects with type 2 diabetes, chronic kidney disease and diabetes, and acute ischaemic stroke.

Table 1 summarizes the clinical trial data and provides information on the study site, the design of the study, the aims, the dosing ranges and the participants included in the studies.

Table 1: Clinical Trials of DM199

**COMPLETED STUDIES**				
**Study Number and Location**	**Design**	**Aims**	**Dosing regime**	**Participants**
DMA-Clin-199-2013-001<br>Netherlands	Phase 1/2a, double-blinded, placebo controlled single site study	Evaluate safety and tolerability<br>Pharmacokinetics of subcutaneous DM199	Part A: healthy volunteers<br>DM199: 1.5, 5, 15, 30, 50 µg/kg or placebo SC<br>Each subject received both a dose of DM199 and placebo<br>Single dose of either 3 or 15 µg/kg SC or placebo	DM199: 30<br>Placebo: 32
			Part B: Diabetic subjects<br>DM199 0.3, 1.5, 15 µg/kg or placebo SC as a single-ascending dose	DM199 7<br>Placebo 11

			Part C: healthy volunteers<br>DM199 3 µg/kg or placebo once every 72 hours for a total of 6 doses over 16 days SC<br>DM199 15 µg/kg on Days 1, 4, and 7 and DM199 25 µg/kg on Days 10, 13, and 16 for a total of 6 doses of DM199 SC	DM199 12<br>Placebo 6
			Part D: diabetic subjects<br>DM199 3 or 15 µg/kg or placebo SC once every 72 hours for a total of 10 doses over 28 days	DM199 25<br>Placebo 9
DM199-2016-001<br>Australia<br>Single study site	Open label, Phase 1b, ascending IV dose administration and comparative pharmacokinetic study of IV and SC DM199 administration<br><br>Study duration: 32 days	Evaluate safety and tolerability of single IV dose administration and comparative bioavailability of SC and IV administration	Part A: DM199 0.25, 0.5, 0.75, and 1.0 µg/kg IV single 30-minute infusion	DM199: 12
			Part B: B single 0.75 µg/kg IV dose or a single 3.0 µg/kg dose SC	DM199 24
DM199-2017-001<br>(ReMEDy 1)<br>Australia<br>12 Sites	Phase 2 randomized, double-blind, placebo-controlled, trial<br><br>Study duration: 90 days	Assess the safety and tolerability of single IV dose followed by SC dose administration of DM199 in subjects with acute ischaemic stroke.	DM199 1 µg/kg IV 40-minute infusion (polyolefin bag) followed by 3 µg/kg SC dose 2-12 hours after IV infusion and then 3 ug/kg SC dose every 72 hours over 22 days for a total of eight SC doses<br>OR<br>Identical placebo	DM199: 46<br>Placebo: 45
DM199-2018-001<br>U.S.<br>3 Sites	Phase 1b, multi-center, open-label, dose ranging study in subjects with chronic kidney disease and diabetes<br><br>Study duration: 11 days	Evaluate safety and tolerability Pharmacokinetics of three subcutaneous dose levels in Type 1 or Type 2 diabetes and chronic kidney disease	Type 1 or 2 diabetes and CKD Stage 3: 3, 5, or 8 µg/kg SC<br><br>Type 1 or 2 diabetes and CKD Stage 4: 3 µg/kg SC	DM199: 33
DM199-2019-001<br>U.S.<br>15 sites	Phase 2, multi-center, open label, basket study<br><br>Study duration: 16 weeks	Evaluate safety and efficacy of two SC doses of DM199 in three different stage 2 chronic kidney disease subpopulations	SC dose of DM199: 2.0 or 5.0 µg/kg SC twice weekly for 95 days	1) 24 African American participants with hypertension<br>2) 25 participants with IgA nephropathy<br>3) 35 participants with Type 2 diabetes
DM199-2023-001<br>Australia<br>Single site	Phase 1c, Open Label, Single Ascending Dose Study	Evaluate safety, tolerability, and pharmacokinetics of DM199 Administered IV with PVC Bag in Adult	Part A:<br>3 healthy participants receiving 0.1 µg/kg DM199 in an infusion starting at 35 mL/hr for 15 minutes and if tolerated, then increased to maximum of 75 mL/hr to complete a 50 mL infusion in approximately 50 minutes.<br><br>Sequential planned cohorts of 3 participants receiving escalating dose of DM199 up to 0.5 µg/kg IV given over an approximately 50-minute period (35 mL/hr first 15 minutes and if tolerated, increased to maximum of 75 mL/hr to complete 50 mL infusion), not to exceed a total of 50 µg of DM199<br><br>Part B:<br>Cohort of 3 participants recently started on ACE inhibitor medications with a last dose >24 hours prior to IV start)<br>35 mL/hr first 15 minutes and if tolerated increased to maximum of 75 mL/hr to complete 50 mL infusion	Part A: Healthy participants (9)<br><br>Part B<br>Adults recently taking ACE Inhibitors (3)
**ONGOING STUDIES**				

DM199-2021-001 (ReMEDy 2)<br>U.S.<br>70 Sites	Phase 2/3 adaptive design, randomized, double-blind, placebo-controlled trial<br><br>Study Duration: 90 days	Evaluate safety, tolerability, and efficacy for treatment of acute ischaemic stroke	IV dose of 0.5 µg/kg at 35 mL/hr for the first 15 minutes of the infusion followed by an increase to 75 mL/hr to complete the IV infusion, only if no hypotension occurs during the first 15 minutes and then SC dose of 3 ug/kg twice per week over 21 days for a total of 7 SC doses.<br>OR<br>Matching placebo	364 subjects with acute ischaemic stroke unable to receive thrombolytics or mechanical thrombectomy

Summary of completed clinical studies

In the **Netherlands**, DM199 was administered by subcutaneous injection in a Phase 1/2 placebo-controlled clinical trial including 42 healthy volunteers and 32 participants with type 2 diabetes mellitus. The maximum tolerated dose was determined to be 30 µg/kg. At a dose of 50 µg/kg and higher, the majority of normotensive study participants experienced modest decreases in systolic and diastolic blood pressure and postural orthostatic hypotension. Some participants experienced mild to moderate adverse events at a dose of 25.0 µg/kg. These are presented below and include headache, nausea and hypotension. Chronic 28-day dosing of 3.0 and 15.0 µg/kg given every 72 hours to subjects was generally safe and well-tolerated.

Single subcutaneous doses of DM199 in healthy subjects showed proportional exposure. Total body clearance was low at approximately 7 mL/hr/kg, and volume of distribution was larger than human plasma volume. The mean terminal elimination phase half-life estimates ranged from 50 to 64 hours.

Repeat subcutaneous dosing of DM199 once every three days (at 3.0 and 15.0 µg/kg) resulted in modest accumulation and were dose proportional. Median time to reach maximum plasma concentration was 12 hours for both dose levels. Mean total body clearance was low and the observed volume of distributions was again larger than human plasma volume. The corresponding mean apparent terminal elimination phase half-life estimates were similar to Day 1 values at approximately 50 hours.

DM199 was also tested in a two-part Phase 1B single site bridging study in **Australia**. Thirty-six healthy volunteers were enrolled. The first part investigated the safety, tolerability, and pharmacokinetics in four different doses (0.25, 0.50, 0.75 and 1.0 µg/kg) of intravenous administration. The second part compared the safety, tolerability, and pharmacokinetic profile of a single intravenous (0.75 µg/kg) dose and a single subcutaneous (3.0 µg/kg) dose. DM199 was safe and well tolerated in both parts of the study. The pharmacokinetic profile following a 30-minute intravenous infusion showed peak plasma concentrations around 30-minutes post-dose and declined exponentially after the infusion was finished. The peak concentration following subcutaneous dosing was more gradual and the elimination and absorption was longer.

DM199 was then tested in a Phase IB study in the **United States**: A Multi-Center Open-label Investigation to Assess the Pharmacokinetics, Safety and Tolerability of DM199 in Patients with Diabetes Mellitus and Chronic Kidney Disease. The single dose study assigned participants to one of the following doses 3.0 µg/kg, 5.0 µg/kg or 8.0 µg/kg SC with no safety or tolerability issues, and no serious adverse events were observed.

DM199 was also tested in a randomized, multi-center phase 2 study in **Australia (ReMEDy)**. Participants with acute ischemic stroke were randomized 1:1 to receive placebo or DM199 administered by a single intravenous dose followed by eight subsequent doses. Results demonstrated that DM199 was safe and well tolerated. The pharmacodynamic data correlated with the DM199 mechanism of action activity and differentiated DM199 from placebo.

In summary, DM199 has been used intravenously at doses ranging from 0.25-1ug/kg, typically 0.5ug/kg- and subcutaneously at doses ranging from 0.3-50ug/kg, most commonly 2-5ug/kg SC. Orthostatic hypotension was most commonly reported among those receiving the highest doses (ie 50ug/kg SC).

2.4 Contraindications

Hypotension

DM199 may increase bradykinin activity resulting in systemic vasodilation. It therefore should not be administered to any subject with a mean arterial blood pressure less than 65 mmHg. Of note is that DM199 at therapeutic doses did not decrease blood pressure in normotensive individuals. This is listed as a contraindication to be cautious.

Angiotensin-converting enzyme inhibitors

DM199 should not be initiated in a subject who has received an angiotensin-converting enzyme inhibitor within 24 hours. Angiotensin-converting enzyme in the body usually inactivates bradykinin, Hence, angiotensin-converting enzyme inhibitors prevent the breakdown of bradykinin. The presence of too much bradykinin could exaggerate the biological effects of DM199. Subjects should also not be started on an angiotensin-converting enzyme inhibitor for at least 8 days after the last dose of the study drug.

This contraindication is not an issue if DM199 is used to treat preeclampsia during pregnancy or fetal growth restriction. Angiotensin-converting enzyme inhibitors are contra-indicated in pregnancy.

History of angioedema or hereditary angioedema

DM199 should not be initiated in a subject who has a history of angioedema or hereditary angioedema as this condition may be brought on by or exacerbated by the drug.

2.5 Adverse events arising in specific clinical trial cohorts

Healthy volunteers

The most common adverse events of DM199 in healthy volunteers when given subcutaneous were headache (33.3%). Other reported adverse events include nausea, postural dizziness, orthostatic hypotension and injection site reaction. Orthostatic hypotension was common with very high doses of DM199 (83% with DM199 50µg/kg)**.** The most frequent adverse events in healthy volunteers given intravenous DM199 were headache (41.7%), erythema (25%) and dizziness (16.7%).
No serious adverse events have been reported.

Type 2 diabetes

Injection site related reactions and diarrhoea were the most frequently reported adverse events. One subject had a severe adverse event of convulsions after 8 of the scheduled 10 doses. The event was deemed to be unrelated to DM199.

Chronic kidney disease and diabetes

The most frequently reported adverse events were orthostatic hypotension (27.3%) and injection site erythema (15.2%). There were no serious adverse events reported.

2.6 Drug interactions

DM199 has been used with many concomitant medications. DM199 is a recombinant tissue kallikrein-1 does not undergo CYP450 metabolism and is not known to inhibit or induce CYP450 enzymes or any other transporters. No pharmacokinetic interactions are therefore expected or known. However, DM199 may have a pharmacodynamic interaction with angiotensin-converting enzyme inhibitors as explained in the earlier section.

The following medications that are commonly used in pregnancy do not have drug interactions with DM199: calcium channel blockers (amlodipine and nifedipine), vitamins (folic acid), minerals (calcium), antithrombotic agents (warfarin, enoxaparin sodium and heparin), diabetic drugs (metformin and insulin), beta blocking agents (labetalol), analgesics (opioids, fentanyl), antianemia preparations (ferrous sulphate, folic acid, vitamin B12), antihypertensives (hydralazine, prazosin), reflux and peptic ulcer disease (omeprazole, esomeprazole and lansoprazole), antibiotics (amoxicillin, azithromycin, ceftriaxone, vancomycin, meropenem and nitrofurantoin), psycholeptics (sertraline, venlafaxine) antihistamines, corticosteroids and antiretroviral medications for HIV infection.

3. Study Design

3.1 Overview

We will start with **Part 1, an open label phase 1B ascending dose finding study**. We will recruit women diagnosed with preeclampsia, for planned delivery within 72 hours, who are acutely hypertensive. We will escalate the dose of DM199 in groups of three participants (administering an initial intravenous loading dose, followed by subcutaneous injection). We will embed pharmacokinetics studies.

If DM199 is safe AND an effective dose is determined, we will then proceed to **Part 2: an open label phase II study**. It will further assess safety, tolerability and efficacy in three related populations all affected by preeclampsia and/or fetal growth restriction.

For part 2, there will be 3 sub-cohorts, each recruiting 30 participants. They are:

Sub cohort 1: women with preeclampsia, acutely hypertensive and required delivery within 72 hours.

Sub cohort 2: women with preterm preeclampsia and are suitable for expectant management,

Sub cohort 3: women with preterm fetal growth restriction and are suitable for expectant management.

We will collect a range of outputs that focus on safety, but also blood pressure reduction and other outputs that may provide early evidence of efficacy. Each sub cohort will have inclusion/exclusion criteria and outputs that vary slightly because they represent different patient populations. Hence, they are listed separately.

3.2 Part 1- Dose finding

Type of study

Open label phase 1B ascending dose finding study

Site

Tygerberg Hospital, Western Cape Provence, South Africa

Aim

To determine a safe and effective dose of DM199 in pregnant women diagnosed with preeclampsia who are hypertensive.

Inclusion and exclusion criteria

Inclusion criteria

- A diagnosis of preeclampsia
- Systolic blood pressure ≥150mmHg and/or a diastolic blood pressure ≥100mmHg
- Gestational age between 27 weeks 0 days and 42 weeks 0 days
- Viable singleton pregnancy
- Admitted for inpatient hospital management
- Delivery is planned within the next 72 hours

The decision for the need for delivery will be made by the treating clinical team and is based on the Protocol for the management of Hypertensive Disorders in Pregnancy at Tygerberg Hospital (https://www.obstyger.co.za/Downloads/TBH_2019%20Hypertension%20in%20pregnancy.pdf).

Exclusion criteria

- Severe complications of preeclampsia which include eclampsia, pulmonary edema, HELLP syndrome, severe renal involvement, cerebrovascular event is defined as an ischaemic or haemorrhagic stroke associated with clinical symptoms and definitive signs on imaging and or a liver haematoma or rupture
- Placental abruption
- Clinical infection eg. chorioamnionitis
- Underlying maternal cardiac disease including a significant arrhythmia, a conduction abnormality or severe valvular disease or congenital or acquired heart disease
- Significant maternal vascular disease eg. renal artery stenosis
- Patient is unable, or unwilling to give consent, or is under the age of 18.
- Suspicion or diagnosis of a major fetal anomaly or malformation or chromosomal abnormality. A major fetal anomaly is defined as anomalies or malformations that create significant medical problems for the neonate or that require specific surgical or medical management.
- Established fetal compromise that necessitates urgent delivery
- History of clinically significant allergic reactions such as angioedema or anaphylaxis requiring hospitalization or familial angioedema
- Participant is currently participating in or has participated in a study using an investigational device or drug or received an investigational drug or investigational use of a licensed drug within 30 days prior to screening
- Women with an active malignancy

Design

This is an open label, phase 1, single centre, ascending dose study. The objective is to find a well-tolerated intravenous (IV) dose, followed by an equivalent subcutaneous (SC) dose. The rationale to deem the SC doses as equivalent is based on prior pharmacokinetic studies on DM199 performed on non-pregnant people. Sequential groups of 3 participants will receive planned escalating doses of DM199 (IV followed by SC) up to a maximum subcutaneous dose of 15ug/kg (see table 2 below).

The intravenous dose will be calculated using the participants weight and will be diluted into 50 mL normal saline in a PVC (Baxter VIAFLEX) bag with PVC tubing and given by a controlled IV infusion pump. The infusion will be started at 35mL/hr for the first 15 minutes and then increased to 75ml/hr to complete the infusion. Subcutaneous doses (undiluted) will be given 2 hours after the initiation of the IV dose. The aim of the SC dose is to maintain a stable plasma concentration of DM 199. The SC dose will be calculated using body weight and given as a subcutaneous injection as described in the section of DM199 drug information.

The traditional dose escalation 3 + 3 design will be used with a minimum of 3 participants enrolled per cohort and observed for toxicity at each dose. Enrolment in the next dosing cohort will proceed if the 3 participants initially enrolled in a dose cohort do not experience a dose-limiting toxicity (DLT).

A DLT includes the following

- maternal hypotension (defined as grade 3 or higher adverse event requiring medical treatment or mean arterial blood pressure less than 65mmHg)
- allergic or hypersensitivity reaction (defined as grade 3 or higher adverse event requiring medical treatment)
- dermatological reaction ((defined as grade 3 or higher adverse event requiring medical treatment)
- any other reaction that the investigators feel meets the criteria for a DLT (which includes adverse events graded as 3 or higher)

If 1 of the initial 3 participants experience a DLT, additional participants will be enrolled at that dose for a minimum of 6 DLT-evaluable patients. If ≤ 1 of the 6 evaluable patients experience a DLT, dose escalation will proceed to the next cohort and its predefined dose. If ≥ 2 DLTs are observed in the 6 evaluable participants at a given dose, dose escalation will be stopped with DSMB evaluation.

Dose escalation will continue, as long as there are no DLT events, until the maximum dose is reached, or the participants have reached a therapeutic blood pressure target which is at or below 140mmHg systolic and 100 diastolic, according to usual clinical practice, on 3 consecutive measurements.

Each group of three participants given the same amount of drug will be dosed as follows: 1 sentinel participant will be dosed, and the Principal Investigator will evaluate the sentinel participant after completion of the IV infusion. The PI will then decide whether to approve and commence the subcutaneous dosing (except for the first cohort which will only receive IV dosing). Once the sentinel participant has delivered, the Principal Investigator will then decide whether to approve and commence dosing of the 2 additional participants that will receive the same dose, or whether a discussion is needed with the trial steering committee and/or the data safety monitoring committee.

Whenever the dose is escalated (which occurs after three participants have received the same dose), the same process described above will take place.

If a participant meets any of the infusion stopping criteria, the infusion will immediately be stopped. No additional participants will be dosed at the same dose level until there has been a discussion with the data safety monitoring committee. Stopping criteria of the infusion are defined as: symptoms of mental status changes, diaphoresis, presyncope, significant dizziness or light-headedness.

Dose rationale

The dosing rationale of DM199 in pregnancy is based on previous pharmacokinetic studies (see section DM199 study drug information). As this is a first in pregnancy study, we will start with an extremely low dose of DM199.

Based on previous studies in non-pregnant individuals, significant blood pressure decreases are expected to occur when the dose is 1.5 µg/kg or higher. To be cautious, we will be starting with a dose that is 15 times lower than this (0.1 µg/kg). The dose escalation is presented in table 2 below. The highest dose is within the safety ranges and is within the FDA approved dose range. The FDA has approved a maximum SC dose of 15µg/kg for human administration, and we will not exceed this limit. The subcutaneous dose that is equivalent to the IV dose is six times higher.

The first cohort will only receive an IV dose. If this is tolerated, we will repeat this low dose and add a SC dose. The SC dose is added as the half-life of the IV dose is short.

If tolerated, we will then sequentially increase the dose until a clinical response is seen (blood pressure reduction) or a maximum dose is reached.

Table 2: DM 199 dosages for Part 1

Cohort (each cohort will include 3 participants)	IV DM199 dose (µg/kg)*	2-hour SC DM199 dose (µg/kg)**
1	0.1	None
2	0.1	0.6
3	0.2	1.2
4	0.5	3
5	0.75	4.5
6	1.0	6
7	1.25	7.5
8	1.5	9
9	2	12
10	2.5	15

*Maximum IV dose will not be higher than 2.5µg/kg
**Maximum SC dose will not be higher than 15µg/kg

Sample size

For the ascending dose finding study, sequential cohorts of 3 subjects at each dose will be enrolled. Up to 10 different doses will be tested with a maximum of 30 women enrolled.

Participants who withdraw early or are lost to follow-up without completing the study will be replaced to ensure that 3 women are included in each group.

If a therapeutic dose of DM199 is discovered, we will then confirm that the dose is effective in 6 women with preeclampsia diagnosed before 34 (+0) weeks gestation and 6 women diagnosed with preeclampsia from 34 (+0) weeks, and over.

Outcomes

Primary outcomes

Safety

- Incidence of treatment emergent adverse events
- Umbilical cord blood levels of DM199 after birth

Efficacy

- Change in maternal blood pressure from baseline assessed immediately after the completion of the infusion, 30 minutes post-infusion and 24 hours after the IV dose
  The average of 3 consecutive blood pressure measurements will be used at each timepoint

Secondary outcomes

- Change in maternal blood pressure from baseline to delivery
- Maternal pharmacokinetic profile of DM199 in preeclampsia
- Uterine contractions

Exploratory outcomes

- Maternal feedback on tolerability
- Episodes of severe hypertension and hypotension

- Use of other antihypertensive agents
- Changes in uterine artery, ophthalmic artery and fetal Doppler parameters
- Changes of clinical biomarkers of disease severity including haemoglobin, platelet, urea, creatinine and proteinuria levels
- Adverse maternal and perinatal outcomes as defined by the Delphi consensus on preeclampsia adverse outcomes(49)
- Changes in plasma biomarkers associated with endothelial dysfunction (between maternal plasma samples collected just prior to administration and samples post administration).
- Changes in flow mediated blood vessel dilatation
- Changes in cerebral autoregulation

Pharmacokinetics

Plasma concentrations of DM199 will be collected at baseline, before treatment, and at multiple timepoints on day 1, 2 and 3 (or up until the time of delivery). These will be measured to determine the pharmacokinetics of DM 199. Pharmacokinetic studies will stop at delivery.

Table 3: Pharmacokinetic sampling for Part 1

DAY	TIMING OF SAMPLE
Day 1	Pre- IV dose, as close to the IV start time as possible (Baseline)
	5 minutes ±2 minutes after IV start time
	10 minutes ±2 minutes after IV start time
	15 minutes ±2 minutes after IV start time
	20 minutes ±2 minutes after IV start time
	30 minutes ±2 minutes after IV start time
	40 minutes ±2 minutes after IV start time
	50 minutes ±2 minutes after IV start time
	60 minutes ±5 minutes after IV start time
	90 minutes ±5 minutes after IV start time
	2 hours ±10 minutes after IV start time before SC injection
	3 hours ±10 minutes after IV start time
	4 hours ±10 minutes after IV start time
	8 hours ±10 minutes after IV start time
	12 hours ±10 minutes after IV start time
Day 2	24 hours ±10 minutes after IV start time
Day 3	48 hours ±30 minutes after IV start time
Delivery	Maternal blood sample
	Cord blood sample

The following pharmacokinetic parameters will be determined or calculated from the plasma concentration-time data.

$\%AUC_{extra}$	percentage of estimated part for the calculation of $AUC_{0-\infty}$<br>$((AUC_{0-\infty}-AUC_{0-t})/AUC_{0-\infty})*100\%$
λz	terminal elimination rate
AUC_{0-t}	area under the plasma concentration-time curve up to time t, where t is the last point with concentrations above the lower limit of quantitation

$AUC_{0-\infty}$	total area under the plasma concentration-time curve from time 0 to infinity<br>Calculated as: $AUC_{0-\infty} = AUC_{0-t} + C_{last}/k_{el}$,<br>where C_{last} is the last measurable plasma concentration
CL	apparent clearance
C_{max}	observed maximum plasma concentration
k_{el}	terminal elimination rate constant
$t_{1/2}$	terminal elimination half-life calculated as $0.693/k_{el}$
t_{last}	time of last measurable plasma concentration
t_{max}	time to attain maximum plasma concentration
Vz	apparent volume of distribution at terminal phase

Primary pharmacokinetic parameters will include:

Enzyme-linked immunoassay for DM199 (DM199 plasma concentration), C_{max} , AUC_{0-t} and $AUC_{0-\infty}$

Secondary PK parameters will include:

t_{max}, t_{last}, $t_{1/2}$, λz, CL and V_z

If an effective blood pressure lowering dose of DM199 is established and there are no safety concerns, we will proceed with Part 2 of the study

3.3 Part 2- safety, tolerability and pharmacokinetic study

Type of study

Open label phase II study assessing safety, tolerability and efficacy

Site

Tygerberg Hospital, Tygerberg, Western Cape Provence, South Africa

Aim

Evaluate the safety, tolerability and pharmacokinetics of DM199 in women with preeclampsia and/or fetal growth restriction

Cohorts

Three sub cohorts will be studied (n=30 per cohort). DM199 will be administered to:

1) women with preeclampsia who are hypertensive and require delivery within 72 hours,
2) women with preterm preeclampsia undergoing expectant management until 34 weeks gestation
3) women with pregnancies complicated by preterm fetal growth restriction.

Sample size

A total of 90 women will be included with 30 women in each sub-cohort.

Any early withdrawal or loss to follow-up (i.e. participants who do not complete the study) will be replaced to ensure that 30 women are recruited in each cohort. The number of participants that do not complete the study will be summarised with a breakdown of reasons for withdrawal.

Inclusion and exclusion criteria for the three sub cohorts

General inclusion criteria for the three sub cohorts

- Viable singleton pregnancy
- Diagnosis of preeclampsia and/or fetal growth restriction (sub cohort 3)
- Admitted for inpatient management until delivery

Specific inclusion criteria for each sub cohort

Sub cohort 2.1: Preeclampsia requiring delivery within 72 hours

- Women diagnosed with preeclampsia or superimposed preeclampsia who presently have a systolic blood pressure above 150mmHg and a diastolic blood pressure above 100mmHg
- Gestational age between 27 + 0 weeks and 42 + 0 weeks
- Delivery is planned within the next 72 hours

Sub cohort 2.2: Preterm preeclampsia undergoing expectant management

- Women with preterm preeclampsia and deemed suitable by the clinical team for expectant management of preeclampsia: i.e. hold off delivery to gain gestation and reduce prematurity (as described by Hall)(50)
- Gestational age between 27 + 0 weeks and 32 + 6 weeks

Sub cohort 2.3: Fetal growth restriction

- Diagnosis of fetal growth restriction, defined as less than the 3rd centile on fetal growth charts.
- No evidence of fetal compromise that warrants immediate delivery.
- Gestational age between 27 + 0 weeks and 32 + 6 weeks.
- For this sub cohort, there may be a co-existing diagnosis of preeclampsia, but they do not have to have preeclampsia.
- No evidence of a fetal infection including syphilis, parvovirus or cytomegalovirus.
- The mother must not have a medical disorder preventing her from having normal food intake (e.g. hyperemesis gravidarum, Crohn's disease, ulcerative colitis, anorexia or bulimia).

General exclusion criteria for the three sub cohorts

- Patient is unable, or unwilling to give consent, or is under the age of 18.
- Hypotension, defined as a systolic blood pressure (BP) <90mmHg and/or a diastolic BP <60mmHg and or a mean arterial BP <65mmHg
- Severe complications of preeclampsia which include eclampsia, stroke, pulmonary edema, HELLP syndrome, severe renal involvement and liver rupture or haematoma
- Clinical infection eg. Chorioamnionitis
- Underlying maternal cardiac disease including a significant arrhythmia, a conduction abnormality or severe valvular disease or congenital or acquired heart disease
- Significant maternal vascular disease eg. renal artery stenosis
- No adequate maternal venous access for sampling
- Patient is unable, or unwilling to give consent, or aged <18 years.

- Suspicion of a major fetal anomaly or malformation or chromosomal abnormality. A major fetal anomaly is defined as anomalies or malformations that create significant medical problems for the patient or that require specific surgical or medical management.
- Fetal compromise that necessitates urgent delivery
- Cerebrovascular event, defined as an ischaemic or haemorrhagic stroke associated with clinical symptoms and definitive signs on imaging.
- Liver haematoma or rupture
- Placental abruption
- Placenta accreta spectrum disorder
- History of clinically significant allergic reactions such as angioedema or anaphylaxis requiring hospitalization or familial angioedema
- Participant is currently participating in or has participated in a study using an investigational device or drug or received an investigational drug or investigational use of a licensed drug within 30 days prior to screening
- Women with an active malignancy

Outcomes for the three sub cohorts

Sub cohort 2.1: Preeclampsia requiring delivery within 72 hours

Primary outcome

Efficacy

- Change in maternal blood pressure from baseline assessed immediately after the completion of the infusion, at 30 minutes post-infusion and 24 hours after the initial dose
  The average of 3 consecutive blood pressure measurements will be used at each timepoint

Safety

- Incidence of treatment emergent events
- Umbilical cord blood levels of DM199 after birth

Secondary outcomes

- Uterine contractions
- Episodes of severe hypertension or hypotension after administration of DM199
- Use of other antihypertensive agents
- Changes in uterine and ophthalmic artery Doppler parameters

Exploratory outcomes

- Maternal feedback on tolerability
- Changes in fetal Doppler parameters
- Changes of clinical biomarkers of disease severity including haemoglobin, platelet, urea, creatinine and proteinuria levels
- Adverse maternal and perinatal outcomes as defined by the Delphi consensus on preeclampsia adverse outcomes(49)
- Sparse maternal pharmacokinetic profiling of DM199

- Changes associated with clinical biomarkers of disease severity in blood and urine – e.g. haemoglobin, renal, liver function tests and proteinuria via (between maternal blood and urine samples collected just prior to administration and samples post administration).
- Adverse maternal and perinatal/neonatal outcomes as defined by the Delphi consensus on preeclampsia adverse outcomes(49)
- Changes in flow mediated blood vessel dilatation
- Changes in cerebral autoregulation

Sub-cohort 2.2: Preterm preeclampsia undergoing expectant management

Primary outcome

Efficacy

- Prolongation of pregnancy (measured from time of first dose to delivery)
- Change in 24-hour protein creatinine ratio one week after enrolment, compared to baseline values
- Need to increase or decrease other antihypertensive agents

Safety

- Incidence of treatment emergent adverse events
- Umbilical cord blood levels of DM199 after birth

Secondary outcomes

- Change in maternal blood pressure from baseline
- Number of women reaching 34+0 weeks gestation
- Episodes of severe hypertension or hypotension
- Uterine contractions
- Changes in uterine artery or ophthalmic artery Doppler flow
- Changes in fetal Doppler parameters
- Neonatal length of stay at Tygerberg Hospital and overall in any hospital

Exploratory outcomes

- Maternal feedback on tolerability
- Sparse maternal pharmacokinetic profiling of DM199
- Changes associated with clinical biomarkers of disease severity in blood and urine – e.g. haemoglobin, renal, liver function tests and proteinuria.
- Adverse maternal and perinatal outcomes as defined by the Delphi consensus(49)
- Changes in flow mediated blood vessel dilatation
- Changes in cerebral autoregulation

Cohort 2.3: Fetal growth restriction

Primary outcome

Efficacy

- Changes in uterine artery and ophthalmic artery Doppler flow
- Changes in fetal Doppler parameters
- Birthweight centile

Safety

- Incidence of treatment emergent adverse events
- Umbilical cord blood levels of DM199 after birth

Secondary outcomes

- Prolongation of gestation (measured from time of first dose to delivery)
- Fetal growth trajectory (if two ultrasounds measuring fetal growth are done during the pregnancy)
- Changes in maternal blood pressure
- Use of antihypertensive medication (if unmedicated at enrolment or the need to increase or decrease other antihypertensive agents

Exploratory outcomes

- Maternal feedback on tolerability
- Sparse maternal pharmacokinetic profiling of DM199
- Changes in flow mediated blood vessel dilatation
- Changes in plasma biomarkers associated with fetal growth restriction
- Adverse maternal and perinatal/neonatal outcomes

Dose

The doses for Part 2 will be calculated based on Part 1 and findings from Part 2.

Sub cohort 2.1: Preeclampsia requiring delivery within 72 hours

An IV and SC dose will be given

Sub cohort 2.2: Preterm preeclampsia undergoing expectant management

An IV and SC dose will be given. The SC dose will be repeated every 72 hours until delivery.

The dose will be decided upon based on findings from Part 1 and Part 2.1

Sub cohort 2.3: Fetal growth restriction

A dose will be chosen that is unlikely to significantly drop blood pressure. It will be informed by the experience obtained from the dose finding studies and sub cohorts 2.1 and 2.2. The chosen dose will be first cleared by the data monitoring safety committee before sub cohort 2.3 enrolment commences.

Only SC dosing will be used because there is less necessity to urgently rush the first dose with an IV bolus. The dose will be repeated every 72 hours until delivery.

Pharmacokinetics

Sparse pharmacokinetic sampling will be used for this cohort. Sampling times may include the following time points presented in Table 4.

Table 4: Sparse pharmacokinetic sampling schedule for Part 2

DAY	TIMING OF SAMPLE
Day 1	Pre- dose, as close to the start time as possible (Baseline)
	5 minutes ±2 minutes after start time
	15 minutes ±2 minutes after start time

	30 minutes ±2 minutes after start time
	50 minutes ±2 minutes after start time
	90 minutes ±5 minutes after start time
	4 hours ±10 minutes after start time
	8 hours ±10 minutes after start time
	12 hours ±10 minutes after start time
Day 2	24 hours ±10 minutes after start time
Day 3	48 hours ±30 minutes after start time
Day 4	72 hours ±30 minutes after start time
Delivery	Maternal blood sample
	Cord blood sample

Pharmacokinetics will be analysed as described for Part 1.

3.4 Study duration

We estimate ethics approval, and the South African Health Products Regulatory Approval should take 6 months. We will then obtain provincial hospital approval. Once we have these approvals the medication will be shipped to the trial pharmacy. We will the perform dummy runs before starting recruitment.

The trial will run for 24 to 36 months depending on recruitment pace. The duration of participant involvement in the study will vary according to which sub cohorts they are recruited to.

4. Trial conduct

4.1 Identification and enrolment of participants

Identification

Potential participants will be identified after they have been admitted to Tygerberg Hospital (a major tertiary referral centre in Cape Town, South Africa) with a diagnosis of preeclampsia and/or fetal growth restriction. Research midwives working in the labour ward will identify possible participants. Screening will be done according to inclusion and exclusion criteria.

Consent and recruitment

Consent will be taken by one of the research nurses or study team members. The staff involved in providing routine care for the potential participant will not take informed consent. Information about the study will be given to the patient and a translator (working from a script) if necessary. Information sheets and study details will be given to the patient and any questions about the study will be answered. A translator will be used so that the patient will have the opportunity to go through the information in her own language. If she chooses to participate, she will be asked to provide written informed consent. The consent form will be photocopied so that a signed copy can be given to the participant, while the original copy will be put in the clinical trial notes and a copy kept by the study investigators. The consent forms will be translated into English, Afrikaans and isiXhosa. Only the study investigators and research midwives will be able to take consent. Patients will be given as much time as they need to decide whether they would like to participate. Once the participant has signed consent she will be enrolled into the study.

Enrolment

After obtaining consent, the participants will be assigned to a study drug dosing group. Enrolled participants will be assigned a unique study number that will be used to identify them on source documents, electronic case report forms, and reports. A screening and enrolment log of all participants screened and enrolled and reasons for exclusion will be kept.

A study nurse will monitor the participant from enrolment to delivery under the supervision of a medical doctor supervised by the principal investigator.

4.2 Study procedures

Pre-dosing examinations

Participants will already be having blood pressure and a cardiotocograph (CTG) monitoring as they will be admitted in the antenatal or labour ward. They will have their height and weight measurements checked and the participants body mass index (BMI) will be calculated (BMI [kg/m^2] = body weight [kg]/height^2[m^2]).

Before dosing we will perform an ultrasound examination to assess the fetal condition. We will then insert a separate intravenous catheter for blood sampling if the patient consents. We will then perform a cerebral Doppler and flow mediated blood vessel dilation test (see descriptions below). We will also place a blood pressure cuff and CTG monitoring machine at her bedside which will remain there until she delivers.

Administering medication

The appropriate dose of DM199 will be calculated using the body weight (kg) and then drawn up in a syringe as per the instructions in the study Pharmacy Manual. Drug preparation will be done using sterile aseptic technique.

The IV dose will be diluted in a PVC Baxter VIAFLEX® 50 mL infusion bag. The infusion will be started at 35 mL/hr for the first 15 minutes and will utilize PVC tubing. The IV study drug infusion will be increased to 75 mL/hr to complete the 50 mL IV infusion in approximately 50 minutes as long as the blood pressure remains above the optimal target.

The fetal condition will be continuously monitored while receiving the IV infusion and for at least an hour after the IV infusion with a cardiotocograph machine. An abnormal cardiotocograph will be diagnosed using the National Institute for Health and Care Excellence Guideline on fetal monitoring in labour.

Subcutaneous injection should be on the lateral side of the thighs or arms. Injection sites should be rotated.

Special Investigations

Blood pressure, heart rate and uterine activity will be measured and documented as detailed in table 5. Fetal ultrasound, endothelial function and cerebral autoregulation will also be performed as presented in table 5.

Blood pressure will be measured using an automated Microlife device that is calibrated and validated for use in pregnancy. Trained study nurses will perform all readings. The average of 3 consecutive measurements will be used. An appropriate cuff size will be used. he measurement will be taken at the level of the patients heart.

Cerebral autoregulation will be measured using a non-invasive transcranial Doppler. The ultrasound probe is placed over the maternal temporal window to measure flow in the middle cerebral artery. Doppler measurements include diastolic, systolic and the mean velocity of the middle cerebral artery. These are obtained bilaterally with a transcranial Doppler machine (Delica technologies). A finger probe will be placed on the participants index finger to measure the blood pressure continuously (Finapres technologies) and nasal prongs will be used to measure exhaled carbon dioxide. The end-tidal carbon dioxide values together with the continuous blood pressure readings and Doppler tracing of the middle cerebral artery are used to calculate dynamic cerebral autoregulation through a Fast Fourier transfer algorithm. The autoregulatory index ranges from 0 to 9 where 9 is optimal regulation and 0 translates to no autoregulatory capacity.

Endothelial function will be measured non-invasively using flow mediated dilatation. The diameter of the brachial artery is measured using high-resolution ultrasound while a blood pressure cuff is inflated. The aim is to measure the diameter of the brachial artery before the artery is closed off with the pressure from the blood pressure cuff. From these measurements the flow medicated dilatation value can be calculated.

The ophthalmic artery Doppler will be measured non-invasively with the patient in the supine position after resting for 10 minutes in the left lateral decubitus position. To perform the Doppler, a linear transducer (5–10MHz) will be positioned laterally across the upper eyelid of the pregnant woman's closed eyes. After identifying the maternal ophthalmic artery using color Doppler, the region medial to the optic nerve, approximately 15mm from the optic disc, will be examined. The following parameters will be standardized: insonation angle< 20°, pulse repetition frequency 125 Hz, and sample volume 2 mm. The Doppler parameters will be analyzed after obtaining at least three consecutive uniform waves. The

following parameters will be obtained automatically: PI, RI, peak systolic velocity, second peak velocity, and end-diastolic velocity. The P2 peak systolic velocity (RPV) ratio will be calculated as P2/PSV.

These participants will be seen daily by study nurses until they deliver. Blood pressure, heart rate, and uterine activity will be checked daily. Ultrasound examinations (measuring maternal and fetal Dopplers) will be performed twice a week or as clinically indicated. Endothelial function testing and cerebral autoregulation measurements will be repeated once a week until delivery and then once postpartum. A small volume of extra blood will be collected for later biomarker studies (see 7.6) whenever blood is being drawn for usual clinical purposes.

At delivery a maternal blood sample and a cord blood sample will be collected to measure DM199 levels. The trial treatment will not be given after delivery. Stickers will be placed in the file stating that ACE inhibitors are contraindicated during the trial and for 8 days after the last dose of DM199 (angiotensin receptor blockers are permitted after birth).

A sample of colostrum/breastmilk (up to 2ml) will be collected within 24 hours of birth for pharmacokinetic studies.

The participants and their baby will be seen daily until discharge. They will then be contacted weekly until 6 weeks after the expected due date.

Table 5: Trial schedule

				Collection Time-Point (Collection time is calculated form the start of dosing)																		
	Screening	Pre-Dose	Day 1 Dosing	Day 1 Period After Infusion Start Time														Day 2	Day 3	Day 4* (if still gravid)	Delivery	Discharge	6 weeks postpartum
	Days 1	Day 1 Pre-Dose	Day 1 Dosing	5 (+/- 2) min	10 (+/- 2) min	15 (+/- 2) min	20 (+/- 2 min)	30 (+/- 2 min)	40 (+/- 2 min)	50 (+/- 2 min)	60 (+/- 5 min)	90 (+/- 5 min)	2 hr (+/- 10 min)	3 hr (+/- 10 min)	4 hr (+/- 10 min)	8 hr (+/- 10 min)	12 hr (+/- 10 min)	24 hr (+/- 10 min)	48 hr (+/- 60 min)	72 hrs (+/- 60 min)			
Informed Consent	X																						
Medical History	X																						
Previous and Concomitant Medication	X																X	X	X	X	X	X	X
Physical Exam	X																	X	X	X			
Body Weight & Height (including BMI)	X																						
Urine protein creatinine ratio	X																	X	X	X			
Blood Pressure, heart rate and monitoring uterine activity	X	X		X	X	X	X	X	X	X	X	X	X	X	X	X	X	X	X	X			
Fetal ultrasound		X											X					X	X	X			
Endothelial function testing**		X											X					X	X	X		X	
Cerebral autoregulation**		X											X					X	X	X		X	
Opthalmic artery Doppler		X											X					X	X	X		X	
Study drug administration-DM199 IV			X										X										
Study drug administration-DM199 SC													X							X			
Blood sampling for PK of DM199 (Part 1)		X		X	X	X	X	X	X	X	X	X	X	X	X	X	X	X	X	X	X		
Blood sampling for (Part 2)	X	X		X		X		X		X		X			X		X	X	X		X		
Colostrum/ Breastmilk collection***																		X					
Adverse Event Monitoring	X	X	X	X	X	X	X	X	X	X	X	X	X	X	X	X	X	X	X	X	X	X	X

*For part 2.2 and 2.3 of the study, the participants may continue their pregnancies well beyond four days.

**This will be performed once a therapeutic dose is found

***A colostrum/breastmilk sample will be collected within 24 hours of delivery

Stopping the trial medication

If urgent delivery is required for maternal or fetal indications, the infusion of the trial medication will be stopped immediately.

4.3 Control of drug supply

DM199 will be shipped to the trial pharmacy after all approvals have been obtained. The drug will be shipped with temperature monitoring equipment. An accurate record of the drug supply, shipment, and drug, dispensed with initials and enrolment number with date and time will be kept. Any unused drug supplies will be destroyed at the end of the study.

4.4 Routine pregnancy care

Management for preeclampsia and severe fetal growth restriction involves admission to hospital until delivery, with close maternal and fetal surveillance. Normal clinical care will be maintained throughout the study. Standard antihypertensive medications, betamethasone, magnesium sulphate and all other medications will be administered according to local protocols except that other blood pressure medication will be withheld or decreased if the blood pressure is within target range (titrated to ongoing blood pressure levels in participants).

4.5 Data collection

Baseline clinical information regarding the pregnancy (maternal age, parity, obstetric history, antenatal history, medical history, drug history, allergies, smoking status, alcohol and drug intake). We will also record the degree of proteinuria, any maternal blood tests (including renal function, liver function, blood count) and fetal assessment with ultrasound (includes biometry, estimated fetal weight, Doppler measurements and amniotic fluid measurements).

Ongoing clinical data will be collected. This will include clinical measurements, medications, hematological parameters, biochemical parameters, fetal ultrasound and fetal heart rate assessments. Patient folders will be reviewed after delivery to confirm that the data collected is complete and accurate. Once the patient has delivered the folder will be copied to assure that all the data is recorded.

For medications we will record the drug name (generic name), dose, route, indication and the dates of administration (start and stop).

4.6 Sample collection and storage

Blood samples (maximum of 9 mls) will be collected before the administration of the study dose and repeated using a sparse pharmacokinetic approach.

Blood samples will be processed as soon as they are collected (within 2 hours). Tubes will be labelled with the participant's trial number and the date of collection. The samples will be spun and split into two aliquots. The aliquots will be stored in a -70°C freezer before being transferred to the biorepository at

Stellenbosch University for storage. These samples will be used to measure biomarkers of preeclampsia, fetal growth restriction and endothelial dysfunction

When recruitment is completed the team in Melbourne (led by ST) and Gothenburg (led by LB) will measure biomarkers of preeclampsia and endothelial dysfunction. They include (but not limited to) soluble fms-like tyrosine kinase-1 (sFlt1), placental growth factor (PIGF), soluble endoglin; and circulating biomarkers of endothelial dysfunction being tumour necrosis factor-alpha (TNFa), soluble vascular cell adhesion molecule 1 (sVCAM 1), endothelin 1, pro-inflammatory cytokines, neurofilament light chain, tau (a microtubule-associated protein), glycocalyx degradation products and others. The intention is to see whether any change in a favourable (or unfavourable) direction after the administration of DM199.

4.7 Withdrawal from the study

All participants will be informed that they are free to withdraw from the study at any time, and that this will not affect their clinical care. In the event of a withdrawal, we will ask whether we can still collect further clinical data and include them in the study. Basic clinical data and samples already collected will be included in the analysis in accordance with the consent obtained at trial entry.

The Participant can also be withdrawn from the study by the Investigator for the following reasons:

- Reasons related to the Participant's safety (AE/SAE) or integrity of the study data
- Noncompliance with the protocol
- Difficulty obtaining blood samples
- Termination of the study by the DMSC

5. Safety Monitoring and Procedures

5.1 Definitions

Adverse event

An adverse event is defined as any untoward medical occurrence associated with the use of a drug in humans, whether or not it is considered drug related.

All adverse events will be documented and reported on the data capture sheets.

An adverse event includes

- Any unfavourable clinical signs or symptoms
- Any new illness or disease, or unexpected complications of existing diseases.

The following are not considered adverse effects

- A pre-existing condition (unless it worsens significantly in pregnancy over and above what may be expected where there is a concurrent diagnosis of preeclampsia and/or fetal growth restriction).
- Diagnostic or therapeutic procedures such as surgery.

Treatment-emergent adverse event

A treatment-emergent adverse event (TEAE) is defined as any event that occurs but was not present before exposure to study drug, or any event already present but worsens in severity or frequency after exposure to the study drug.

Suspected Adverse Reaction

A suspected adverse reaction (SAR) is defined as a subset of all AEs for which there is a reasonable possibility that the drug caused the AE. “Reasonable possibility" means there is evidence to suggest a causal relationship between the drug and the adverse event. SAR implies less certainty about causality than adverse reaction, which means any adverse event caused by a drug.

Serious adverse event

A serious adverse event (SAE) will be defined as any of the following

- Maternal death
- Fetal loss or neonatal death
- Event that results in a longer postnatal hospital stay
- Event that results in a persistent or significant disability in the mother or baby
- Congenital or birth defect in the baby that is detected in the post-natal period and was not detected on ultrasound

Serious adverse events will be reported to the ethics committee, the data monitoring and safety committee, the drug manufacturer and the South African Health Products Regulatory Authority within 24 hours of the principal investigator being notified. Monitoring for serious adverse events will occur from the time of signing informed consent until the end of the follow up period.

Serious Unexpected Suspected Adverse Reaction

A serious unexpected suspected adverse reaction (SUSAR) is a suspected adverse reaction that is both serious and unexpected (i.e., an event that meets the definition of a suspected adverse reaction, serious, and unexpected). All SUSARs will be reported to the ethics committee, the data monitoring and safety committee, the drug manufacturer and the South African Health Products Regulatory Authority within 24 hours of the principal investigator being notified.

Expected adverse events

Certain adverse events will be expected as we are treating women with preeclampsia, including sub cohorts with preterm preeclampsia. Women with preterm preeclampsia or fetal growth restriction often need to be delivered early which results in the complications of prematurity. We also expect to have adverse events related to the diagnosis of preeclampsia which may include severe hypertension, eclampsia, intracranial haemorrhage, renal failure, abnormal hepatic function, disseminated intravascular coagulopathy, left ventricular failure, pulmonary oedema and haemorrhage. Complications of prematurity for the newborn that may be expected include sepsis, necrotising enterocolitis, respiratory complications, seizures, hypoglycaemia and intra-ventricular haemorrhage.

All adverse events will be reported to the principal investigator (or other nominated clinician) as soon as they have been identified. Full details including the diagnosis (if possible), the duration, actions taken, treatment given, outcome, causality will be recorded.

If an adverse event is considered potentially related to the trial medication (and not in keeping with preeclampsia or fetal growth restriction) it will be immediately reported to the DSMC, SAHPRA, the ethics committee and the manufacturer. Reporting and handling of adverse events will be in accordance with the Good Clinical Practice (GCP) guidelines.(51) These procedures have been used in previous trials and proven to be efficient and compliant with the GCP principles and data management.

Post-study Adverse Events

A post-study adverse event is defined as any event that occurs outside of the AE reporting period. If the Investigator learns of any SAE, including death, at any time after a Participant has been discharged from the study, and he/she considers the event reasonably related to the study drug, the SAE will be reported to the ethics committee, the data monitoring and safety committee, the drug manufacturer and the South African Health Products Regulatory Authority within 24 hours of the principal investigator being notified.

5.2 Evaluation of Adverse Events

Assessment of Severity

AEs and other symptoms will be graded.

The Investigator will assess severity for each AE. The assessment will be based on the Investigators clinical judgment. The severity should be assigned to one of the categories outlined in the table below.

Table 6: Toxicity Grading

**Systemic (General)**	**Mild (Grade 1)**	**Moderate (Grade 2)**	**Severe (Grade 3)**	**Potentially Life Threatening (Grade 4)**
Nausea/ vomiting	No interference with activity or 1 – 2 episodes/24 hrs	Some interference with activity or > 2 episodes/24 hrs	Prevents daily activity, requires outpatient IV hydration	Hypotensive shock or potentially life threatening
Diarrhoea	2 – 3 loose stools or <400 grams/24 hrs	4 – 5 stools or 400 – 800 grams/24 hours	6 or more watery stools or > 800gms/24 hours or requires outpatient IV hydration	Hypotensive shock or potentially life threatening
Headache	No interference with activity	Repeated use of non-narcotic pain reliever >24 hours or some interference with activity	Significant; any use of narcotic pain reliever or prevents daily activity	Potentially life threatening
Fatigue	No interference with activity	Some interference with activity	Significant; prevents daily activity	Potentially life threatening
Myalgia	No interference with activity	Some interference with activity	Significant; prevents daily activity	Potentially life threatening
Illness or clinical adverse event	No interference with activity	Some interference with activity not requiring medical intervention	Prevents daily activity and requires medical intervention	Potentially life threatening
Facial, oral or airway angioedema	Absent	Absent	Intervention indicated	Life-threatening and urgent intervention is indicated
Flushing	Asymptomatic, clinical or diagnostic observation only	Moderate symptoms	Symptomatic associated with hypotension and/or tachycardia	
Hypotension	Asymptomatic, intervention not indicated	Non-urgent medical intervention indicated	Medical intervention or hospitalization indicated	Life-threatening and urgent intervention indicated
Orthostatic hypotension	Mild unsteadiness or sensation of movement	Moderate unsteadiness or sensation of movement. Near fainting	Fainting, orthostatic collapse	
Local injection site reactions				
Pain	Mild pain	Moderate pain	Severe pain	Emergency Department (ED) visit or hospitalization

Tenderness	Mild discomfort to touch	Discomfort with movement	Significant discomfort at rest	ED visit or hospitalization
Pruritis	Mild or localized	Intense or widespread; intermittent	Intense or widespread; constant	ED visit or hospitalization
Urticaria (hives, welts, wheals)	Urticarial lesions cover <10 Body Surface Area (BSA)	Urticarial lesions covering 10-30% BSA	Urticarial lesions covering >30%BSA	ED visit or hospitalization
Erythema/Redness	Target lesions cover <10% BSA	Target lesions covering 10-30% BSA and associated with skin tenderness	Target lesions covering >30% BSA and associated with oral or genital erosions	Target lesions covering >30% BSA; associated with fluid or electrolyte abnormalities; ICU care or burn unit indicated
Induration/ Swelling	Mild induration, able to move skin parallel to plane (sliding) and perpendicular to skin (pinching up)	Moderate induration, able to slide skin, unable to pinch skin	Severe induration, unable to slide or pinch skin, limiting joint movement or orifice (e.g., mouth, anus)	Generalized; associated with signs of symptoms of impaired breathing or feeding

Local (Injection Site) Adverse Event Intensity

Intensity of the following local injection site AEs should be assessed as described in the table below

Table 7: Grading of Local (Injection Site) Adverse Event Intensity

**Local Reaction to Injectable Product**	**Mild (Grade 1)**	**Moderate (Grade 2)**	**Severe (Grade 3)**	**Potentially Life Threatening (Grade 4)**
Pain	Does not interfere with activity	Repeated use of non-narcotic pain reliever >24 hrs or interferes with activity	Any use of narcotic pain reliever or prevents daily activity	Potentially life threatening
Tenderness	Mild discomfort to touch	Discomfort with movement	Significant discomfort at rest	Potentially life threatening
Erythema/Redness *	2.5 – 5 cm	5.1 – 10 cm	> 10 cm	Necrosis or exfoliative dermatitis
Induration/Swelling **	2.5 – 5 cm and does not interfere with activity	5.1 – 10 cm or interferes with activity	> 10 cm or prevents daily activity	Necrosis

* In addition to grading the measured local reaction at the greatest single diameter

** Induration/Swelling should be evaluated and graded using the functional scale as well as the measurement.

If an AE worsens in severity, it should be updated in the case report records to reflect the new grading. Even if an AE gets milder, the most severe grading should be documented.

An AE that is assessed as severe should not be confused with an SAE. Severity is a category utilized for rating the intensity of an event; and both AEs and SAEs can be assessed as severe.

Adverse events not listed above in the tables will be graded using the latest available version of Common Terminology Criteria for Adverse Events (CTAE) (https://ctep.cancer.gov/protocoldevelopment/electronic_applications/docs/ctcae_v5_quick_reference_5x7.pdf).

Maternal and fetal adverse events will be graded using the Maternal and Fetal Adverse Event Terminology (MFAET) v1.1, 2022 or later versions (https://www.ucl.ac.uk/womens-health/sites/womens_health/files/mfaet_version_1.1_with_instructions_for_use.pdf). (52)

Neonatal adverse events will be graded using the Neonatal Adverse Event Severity Scale (NAESS) (53)

Assessment of Causality

The Investigators will assess the relationship between study drug and the occurrence of each AE. The Investigators will use clinical judgment to determine the relationship. Alternative causes, such as natural history of the underlying diseases, concomitant therapy, other risk factors, and the temporal relationship of the event to the study drug will be considered and investigated.

The Investigators will provide the assessment of causality utilizing one of four possible categories:

- **Unrelated**: Clearly and incontrovertibly due to an underlying or concurrent illness or effect of a concomitant therapy and is not related to the study drug does not meet criteria listed under unlikely, possible, or probable. Events that occur prior to dosing will be classified as unrelated.
- **Unlikely**: Does not follow a reasonable temporal relationship from administration. May have been produced by the participant's medical condition, environmental factors, or other therapies administered.
- **Possible**: Follows a reasonable temporal relationship from administration. May have been produced by the Participant's medical condition, environmental factors, or other therapies administered.
- **Probable**: Clear-cut temporal relationship with improvement on cessation of the study drug. Follows a known pattern of response to the study drug.

5.3 Reporting of Adverse Events

The Investigator will review all AEs that are observed or reported during the study, regardless of their relationship to the study drug. All AEs will be recorded in the electronic case report forms and will be followed to adequate resolution.

All AEs and SAEs will be recorded from study drug administration until study withdrawal or completion of the trial. All AEs will be recorded irrespective of whether they are considered related to study drug. AEs meeting serious criteria will be reported within 1 working day to the DSMC and drug manufacturer.

5.4 Adverse Event Follow-up

After the initial AE report, the investigator will follow up each participant. All AEs will be followed until resolution, until the condition stabilizes, until the event is otherwise explained, or until the participant is lost to follow-up.

5.5 Reasons to stop the trial

If, for any reason, there is a need to stop the trial prematurely the decision will be made by the Data Safety Monitoring Committee.

6. Data management

6.1 Data management

All required clinical data for this trial will be collected in a REDCap web-based electronic database hosted at Stellenbosch University. Participant personal information will be pseudonymized.

A Data Management Plan (DMP) will be developed outlining the procedures used for data review, database cleaning, and issuing and resolving data queries. Procedures for validations and data storage will also be contained within the DMP.

6.2 Case Report Forms

The Investigator is responsible for ensuring the accuracy and completeness of all study documentation. All protocol-required information collected on data capture sheets and transcribed onto a REDCap database. All data will be considered strictly confidential and only the investigators will have access to the database. Forms will be transcribed into REDCap as soon as possible after the data are collected. If data is missing the original data extraction sheets will be reviewed and if needed, the original patient notes will be reviewed. An explanation will be noted on REDCap for any missing data that cannot be retrieved. All data entry will be double checked for inaccuracies.

6.3 Source Documentation

Copies of the source documents including the antenatal card and hospital notes will be added to REDCap. The Investigator will permit study-related monitoring, audits, ethics review and regulatory authority inspections by allowing direct access to all study source data and documents. Data will be collected prospectively by the researchers and data checking and entry of the completed data collection forms will be reviewed.

6.4 Data processing

Data entry and checking will be continuous, and queries will be followed vigorously to ensure clarification without delay. The aim here is to obtain a complete dataset with minimal incorrect entries due to either study-based recording or data entry errors.

7. Statistical analysis and considerations

7.1 Study design

This is an open-label investigation of DM199 in women with preeclampsia and/or fetal growth restriction. The study is intended to assess safety and efficacy. Up to 42 women will be enrolled in the dose finding cohort (Part 1). Ninety women (30 women in each sub-cohort) will be enrolled in Part 2.

7.2 Sample Size Determination

Part 1: The dose finding study is based on the standard model designed for phase 1 dose finding studies.(54)

Part 2: The sample size of thirty participants per study sub cohort is standard and commonly done for similar open label 2 studies. It is expected to provide sufficient data to adequately assess safety. If the drug is safe and there are efficacy signals, the study will provide the preliminary data required to justify a large phase III randomised trial.

7.3 Statistical Methods

We will present the data by study drug groups and overall, as applicable. Statistical methods will be primarily descriptive in nature. Each group will be summarized separately.

Variables will be presented using descriptive statistics. Continuous variables will be presented using number of values (N), mean, standard deviation (SD), median, minimum, and maximum. Categorical variables will be presented using frequency counts and percentages per category. Missing data will not be imputed but will be analysed as missing. Confidence intervals will be two-sided and will use 95% confidence levels unless specified otherwise. Baseline values will be defined as the last observation for each participant prior to the administration of the study drug.

7.4 Outcomes

Safety

All adverse event data will be listed for all participants and summarized. Listings will also include the start and end time and date of the event, relationship to study drug, severity, and action taken for the event

Vital Signs

Changes from baseline in vital signs at each timepoint will be summarized by study cohort and study drug using descriptive statistics. The change from baseline is defined as the post-baseline value minus the baseline value. There will not be any imputation for missing values.

Clinical Laboratory

All laboratory data will be summarized by cohort and study drug dose at each scheduled timepoint using descriptive statistics (n, mean, SD, median, minimum, and maximum). The change-from-baseline variables will be calculated as the post-baseline value minus the value at baseline. Change from baseline on continuous data will be summarized using descriptive statistics at each scheduled time point by study drug

group. For categorical data, change from baseline will be summarized using frequency and proportion at each scheduled timepoint by study drug group.

Individual data listings of laboratory results will be presented for each Participant. Values outside of the laboratory's reference range will be flagged.

Pharmacokinetic and Pharmacodynamic parameters

Blood samples will be collected as detailed in Table 5 for pharmacokinetic profile characterization and to provide data for non-compartmental analysis. A validated ELISA assay will be used to measure DM199 concentration in plasma samples and the results will be analyzed by validated software tools.

For each participant the following PK parameters will be calculated from the concentration time-course data: AUC_{0-t} (area under the curve from time 0 (time of dosing) to the last time point measurable), AUC_{0-inf} (AUC from time point 0 to extrapolated to infinity), C_{max} (maximum observed concentration), AUC_{0-24} (AUC from time point 0 to 24 hour post-dose), T_{max} (the first time when C_{max} was observed), $T_{1/2,app}$ (apparent elimination half-life), CL/F, mean residence time (MRT), and Vz/f (the apparent oral volume of distribution). Both arithmetic and geometric mean values will be used to describe the combined results from all participants from each dose group.

Pharmacodynamic endpoints will be analyzed and used to evaluate any exposure-response relationship. Endpoints will be summarized using descriptive statistics for all time points assessed, including change from baseline for all post-dose assessments, as applicable.

Additional exploratory analysis may be performed to further assess primary and secondary study endpoints and may include additional sub-groups as needed.

8. Study Monitoring and Auditing

Monitoring activities will be conducted according to the protocol, Good Clinical Practice Guidelines, South African Health Products Regulatory Authority (SAHPRA) requirements, and the conditions of approval from the Stellenbosch University Health Research Ethics Committee (HREC). Procedures for conducting monitoring will be documented in the Study Monitoring Plan.

8.1 Monitoring and Source Document Verification

The investigators will facilitate study-related monitoring by providing direct access to all study records. Periodic monitoring will be completed on site. Monitors will check the REDCap electronic case report forms for completeness and accuracy and verify them with source documents. Visits are also intended to review site compliance, administrative records, and to confirm that all adverse events have been reported as required by the protocol. In addition to the monitoring visits, frequent communications (letter, telephone, and e-mail) by the study monitors will ensure that the investigation is conducted according to the protocol, regulatory requirements, and good clinical practice.

Study close-out will be performed by the study monitor upon closure of the study. The close-out visit will consist of reconciliation of all remaining data inconsistencies, obtaining current status determination for all unresolved adverse events, and review of administrative records.

8.2 On-Site Audits/Inspections

The Investigator and study team will assist with any trial related audits and inspections by HREC and SAHPRA. Direct access to source documents, case report forms and other study documents for on-site audits or inspections will be made available. Medical records and other study documents may be copied during audit or inspection provided that all participant identifiers are removed on the copies to ensure confidentiality.

9. Study Administration

9.1 Regulatory and Ethical Considerations

Protocol and Amendment Approvals

All protocol amendments will be submitted to HREC and SAHPRA for approval prior to implementation.

Protocol Deviation Management

A protocol deviation is defined as any intentional or unintentional change to, or noncompliance with, the approved protocol procedures. Deviations may result from the action or inaction of the participant, investigator, or site staff. All deviations will be reported to HREC and SAHPRA in accordance with their reporting policies.

Examples of deviations include, but are not limited to:

- Failure to obtain informed consent.
- Failure to adhere to study inclusion and exclusion criteria.
- Failure to comply with dosing requirements, including administering study drug outside of the time frame specified in the protocol.
- Use of medications that are specifically prohibited in the protocol.
- Incorrect storage of study drug.

The Investigator will document and explain in the participant's source documentation any deviation from the approved protocol.

9.2 Final Report

When the study is completed, the investigator will submit the final clinical study report to HREC, SAHPRA and the drug manufacturer.

The Investigator will supply a full summary of the study results. Results will also be shared with the study participants. The study results will also be posted on publicly available clinical trial registers.

9.3 Timelines

It is anticipated that the study can be completed in approximately 3 years (2024 – 2027). This has been conservatively approximated using data from the PROVE biobank on the incidence of preeclampsia and its complications at Tygerberg Hospital.(55)

Recruitment will begin once we have all the needed approvals including HREC, SAHPRA and the Western Cape Health Department approval. This study will be registered the South African National Health Research Ethics Council (NHREC) and the Pan African Clinical Trials Registry (PACTR) once we have ethics approval. We anticipate that all approvals and registrations will take six to nine months.

9.4 Post trial access to medication

There is no need for post-trial access to medication as we will only be treating participants before delivery.

9.5 Publication of results

The study protocol will be published once it has been approved by ethics. We will aim to publish the protocol in a journal like BMJ Open. Once the trial is complete, we will publish the results of this trial in publicly accessible, peer reviewed journals.

9.6 Role of study funders

The funders of the trial have had no role in the recruitment, data collection, management, analysis and interpretation of the data. They will also have no role in writing the report and the decision to submit the report for publication.

10. Ethical aspects

10.1 Confidentiality

Participant confidentiality will be maintained throughout the study and data and will be kept in accordance with institutional and HREC policies. In order to protect participant confidentiality, each participant will be assigned a unique participant study number. Only on-site study staff directly involved with the participant will have access to identifying participant data. Data collection sheets and any samples collected will only be marked with the participants study number. All data exports will only contain the participant study number.

All personal data on paper format will be stored in a secure location and will be treated as strictly confidential. No data that could identify a participant will be released. All data collected will be stored for a minimum of 15 years or longer as defined by the requirements of the Ethics Committee once the trial is completed.

Participants will be informed that representatives from HREC, Stellenbosch University and or the regulatory authorities may review their medical records to verify the information collected. Personal information may be made available for inspection and if so, it will be handled in strictest confidence and in accordance with local data protection laws.

10.2 Record Retention

The documents to be retained include but are not limited to:

- Original signed informed consent documents for all participants
- Screening log, and enrolment log
- Record of all essential communications relating to the conduct of this study between the Investigator and the DSMC, HREC, trial monitors and SAHPRA
- List of sub-investigators and other appropriately qualified persons to whom the Investigator has delegated trial-related duties, together with their roles, signatures and dates of participation
- Study drug accountability records
- Case report forms

10.3 Compensation for participation

Participants will be compensated in accordance with SAHPRA regulations and guidance. https://www.sahpra.org.za/document/clinical-trial-participant-time-inconvenience-and-expense-tie-compensation-model/

Only admitted patients are included in the trial so they will not have any travel or other costs related to being included in the trial.

10.4 Risks and Benefits

Potential risks

Possible release of prostaglandin E2

In early phase trials on safety of DM 199 (chronic kidney disease cohort), there was a trend towards a rise in blood levels of prostaglandin E2. At 24 hours, median levels of prostaglandin E2 in the blood rose 8% from baseline. This rise was not statistically significant, but it was a small study, and there may be a trend. If prostaglandin E2 were secreted into the blood in biologically relevant amounts, it is possible it could travel to the uterus and cause cervical ripening. Sometimes, this can lead to uterine contractions and labour. Given the modest rise of blood prostaglandin E2 seen in the DM 199 trial so far, we consider this risk as very low. However, we have considered this possibility in the design of our study. The initial 30 to 60 participants (part A and sub cohort 2.1 of part B) we will recruit women where delivery is planned within the next 72 hours. If DM 199 did cause contractions in this cohort it would have minimal clinical impact, and we would not proceed with the fetal growth restriction or preeclampsia studies where the aim was to increase gestation,

A worsening of inflammation

We previously noted the increased production of nitric oxide may reduce a pro-inflammatory response in endothelial cells. However, it is unclear whether giving DM 199 will actually reduce inflammation - the opposite might occur. Bradykinin and bradykinin receptor 1 and 2 activation have been implicated in the progression of COVID 19. In severe COVID 19 there is pathological upregulation of these receptors and an exaggerated response, leading to an increased immune response and hypotension. However, much of the pathological immune response may be mediated through bradykinin receptor 1 which is only upregulated in severe inflammatory disease states or severe infection. Reassuringly, in the early phase trials of DM 199 of healthy volunteers and those with chronic kidney disease, circulating levels of C Reactive Protein (a general biomarker of inflammation) did not increase. In our early phase trials, we will watch out for whether an inflammatory response will happen by measuring C Reactive Protein as well as circulating pro-inflammatory cytokines.

Hypotension causing fetal distress

When a woman is diagnosed with preeclampsia, we give antihypertensive drugs to reduce the blood pressure. We take care not to drop the blood pressure too much, or too quickly. This is because a pronounced fall in blood pressure may reduce the perfusion of the placenta, reducing oxygen delivery to the fetus. Aware of this risk, we will monitor blood pressures closely, start with very low doses and use intravenous dosing as it is short acting. We will also closely monitor the fetal condition with continuous cardiotocograph monitoring. If blood pressures falls excessively and fetal distress is detected, there are intravenous fluids and drugs that we can give with rapid onset to restore blood pressures to normal levels.

Potential Benefits

There are currently no drugs that can treat preeclampsia and fetal growth restriction. A drug that could slow disease progression of either could save the lives of many.

DM 199 is a very promising candidate. It reduces blood pressure by causing vasodilation and may ameliorate the endothelial dysfunction central to the pathophysiology of both preeclampsia and fetal growth restriction. Animal studies and first in human studies have shown it to be safe. As it is a very large protein molecule, it is very unlikely to cross the placenta (proteins cannot cross cellular lipid bilayer walls of cells unless there is an active transport mechanism. No such mechanism has been reported for KLK1, the natural homologue of DM199). It is therefore also unlikely to have any direct effects on the fetus. DM 199 may be the first treatment to ameliorate preeclampsia and fetal growth restriction.

Risk Minimization

DM199 is being evaluated for its use in preeclampsia and fetal growth restriction. This protocol is specifically designed to manage and minimize risk through careful participant selection, dose escalation, starting with very low doses, adherence to the pre-determined assessment schedule to assess participant clinical status, and a two-stage design. We will only move to the second stage of the study if an effective and safe dose is determined.

11. Resources and strengths of the study

11.1 Strengths

1) DM 199 is an ideal drug candidate to potential treat preeclampsia and fetal growth restriction.

2) Our team is the only team in the world that has successfully run serial trials assessing novel therapeutics to treat preeclampsia. We are arguably the leaders in this field.

3) We have previously embedded both pharmacokinetic and biomarker studies in our previous trials. We have the expertise and experience to undertake the current trial.

4) We have a team of experienced clinical trials research nurses.

5) We have an established biobank and experience in collecting clinical data and biological specimens/

6) The women who we recruit into our trials are excited to be involved in research and want to be involved in finding new treatments for preeclampsia and fetal growth restriction.

7) Tygerberg hospital is the largest referral hospital in the Western Cape Provence of South Africa. We treat many women with preeclampsia and fetal growth restriction. This trial is therefore feasible.

8) Tygerberg Hospital has many state of the art facilitates on par with tertiary referral hospitals in high-income countries. It has a neonatal special care unit, a maternal high care unit and an academic centre that actively contributes to the global scientific literature. Thus, running this study here has advantage of being a centre that practises modern obstetrics similar to that in high-income countries.

9) It capitalises on a unique collaboration between Australia and South Africa: Prof Cluver is an academic clinician based at Tygerberg Hospital, Stellenbosch University, South Africa. In 2013, she was the visiting maternal-fetal medicine Fellow at Mercy Hospital, Melbourne University, Australia (where Profs Walker and Tong are based). A close clinical and academic collaboration developed among the investigators who have worked together for the past 10 years.

11.2 Limitations of this study

This study is designed to determine if DM 199 is effective at reducing blood pressure in preeclampsia and to determine if it is safe to administer to pregnancies complicated by preeclampsia and fetal growth restriction. If a safe and effective at reducing blood pressure, a large phase III trial will still be needed to determine if it is a potential treatment for fetal growth restriction and preeclampsia.

References

1. Magee LA, Brown MA, Hall DR, Gupte S, Hennessy A, Karumanchi SA, et al. The 2021 International Society for the Study of Hypertension in Pregnancy classification, diagnosis & management recommendations for international practice. Pregnancy Hypertension. 2022 Mar 1;27:148–69.

2. Chappell LC, Cluver CA, Kingdom J, Tong S. Pre-eclampsia. The Lancet. 2021 Jul 24;398(10297):341–54.

3. Zhang J, Meikle S, Trumble A. Severe maternal morbidity associated with hypertensive disorders in pregnancy in the United States. Hypertension in pregnancy. 2003;22(2):203–12.

4. Abalos E, Cuesta C, Grosso AL, Chou D, Say L. Global and regional estimates of preeclampsia and eclampsia: A systematic review. Vol. 170, European Journal of Obstetrics Gynecology and Reproductive Biology. Elsevier; 2013. p. 1–7.

5. Say L, Chou D, Gemmill A, Tunçalp Ö, Moller AB, Daniels J, et al. Global causes of maternal death: A WHO systematic analysis. The Lancet Global Health. 2014;2(6).

6. Moodley J. Maternal deaths due to hypertensive disorders of pregnancy: data from the 2014-2016 Saving Mothers' Report . Obstetrics and Gynaecology Forum. 2018;28(3):28–32.

7. Wu P, Haththotuwa R, Kwok CS, Babu A, Kotronias RA, Rushton C, et al. Preeclampsia and future cardiovascular health. Vol. 10, Circulation: Cardiovascular Quality and Outcomes. Lippincott Williams and Wilkins; 2017.

8. Levine RJ, Lam C, Qian C, Yu KF, Maynard SE, Sachs BP, et al. Soluble endoglin and other circulating antiangiogenic factors in preeclampsia. New England Journal of Medicine. 2006 Sep 7;355(10):992–1005.

9. Roberts JM, Redman CWG. Pre-eclampsia: more than pregnancy-induced hypertension. The Lancet. 1993 Jun 5;341(8858):1447–51.

10. Levine RJ, Maynard SE, Qian C, Lim KH, England LJ, Yu KF, et al. Circulating angiogenic factors and the risk of preeclampsia. New England Journal of Medicine. 2004;350(7):672–83.

11. Young BC, Levine RJ, Karumanchi SA. Pathogenesis of preeclampsia. Annual Review of Pathological Mechanical Disease. 2010;5:173–92.

12. Hubel CA. Oxidative stress in the pathogenesis of preeclampsia. Proceedings of the Society for Experimental Biology and MedicineSociety for Experimental Biology and Medicine (New York, NY). 1999 Dec;222(3):222–35.

13. Sibai B, Dekker G, Kupferminc M. Pre-eclampsia. Lancet (London, England). 2005 Feb;365(9461):785–99.

14. Tong S, Kaitu'u-Lino TJ, Hastie R, Brownfoot F, Cluver C, Hannan N. Pravastatin, proton-pump inhibitors, metformin, micronutrients, and biologics: new horizons for the prevention or treatment of preeclampsia. American Journal of Obstetrics and Gynecology. 2022 Feb 1;226(2):S1157–70.

15. Brownfoot FC, Hastie R, Hannan NJ, Cannon P, Tuohey L, Parry LJ, et al. Metformin as a prevention and treatment for preeclampsia: effects on soluble fms-like tyrosine kinase 1 and soluble endoglin secretion and endothelial dysfunction. American Journal of Obstetrics and Gynecology. 2016;214(3):356. e1-356. e15.

16. Powe CE, Levine RJ, Karumanchi SA. Preeclampsia, a Disease of the Maternal Endothelium The Role of Antiangiogenic Factors and Implications for Later Cardiovascular Disease. Circulation. 2011;123(24):2856–69.

17. Lau SY, Guild SJ, Barrett CJ, Chen Q, Mccowan L, Jordan V, et al. Tumor Necrosis Factor-Alpha, Interleukin-6, and Interleukin-10 Levels are Altered in Preeclampsia: A Systematic Review and Meta-Analysis. American Journal of Reproductive Immunology. 2013 Nov 1;70(5):412–27.

18. Bouças AP, De Souza BM, Bauer AC, Crispim D. Role of Innate Immunity in Preeclampsia: A Systematic Review. Reproductive Sciences. 2017 Oct 1;24(10):1362–70.

19. Tannetta D, Masliukaite I, Vatish M, Redman C, Sargent I. Update of syncytiotrophoblast derived extracellular vesicles in normal pregnancy and preeclampsia. Journal of reproductive immunology. 2017 Feb 1;119:98–106.

20. Granger JP, Spradley FT, Bakrania BA. The Endothelin System: A Critical Player in the Pathophysiology of Preeclampsia. Current hypertension reports. 2018 Apr 4;20(4):32.

21. von Dadelszen P, Syngelaki A, Akolekar R, Magee LA, Nicolaides KH. Preterm and term pre-eclampsia: Relative burdens of maternal and perinatal complications. BJOG: An International Journal of Obstetrics and Gynaecology. 2023 Apr 1;130(5):524–30.

22. Gestational Hypertension and Preeclampsia: ACOG Practice Bulletin, Number 222. Obstetrics and gynecology. 2020 Jun 1;135(6):e237–60.

23. Hawkes N. Trial of Viagra for fetal growth restriction is halted after baby deaths. BMJ (Clinical research ed). 2018 Jul 25;362:k3247.

24. Groom KM, Ganzevoort W, Alfirevic Z, Lim K, Papageorghiou AT, M. Groom K, et al. Clinicians should stop prescribing sildenafil for fetal growth restriction (FGR): comment from the STRIDER

Consortium. Ultrasound in obstetrics & gynecology : the official journal of the International Society of Ultrasound in Obstetrics and Gynecology. 2018 Sep 1;52(3):295–6.

25. Cluver CA, Hiscock R, Decloedt EH, Hall DR, Schell S, Mol BW, et al. Use of metformin to prolong gestation in preterm pre-eclampsia: randomised, double blind, placebo controlled trial. BMJ. 2021 Sep 23;374:n2103.

26. Paidas MJ, Tita ATN, Macones GA, Saade GA, Ehrenkranz RA, Triche EW, et al. Prospective Randomized Double-Blind, Placebo Controlled Evaluation of the Pharmacokinetics, Safety and Efficacy of Recombinant Antithrombin Versus Placebo in Preterm Preeclampsia (PRESERVE-1). American Journal of Obstetrics and Gynecology. 2020 Aug 8;

27. Saito S, Takagi K, Moriya J, Kobayashi T, Kanayama N, Sameshima H, et al. A randomized phase 3 trial evaluating antithrombin gamma treatment in Japanese patients with early-onset severe preeclampsia (KOUNO-TORI study): Study protocol. Contemporary clinical trials. 2021 Aug 1;107.

28. Ahmed A, Williams D, Cheed V, Middleton L, Ahmad S, Wang K, et al. Pravastatin for early-onset pre-eclampsia: a randomised, blinded, placebo-controlled trial. BJOG: An International Journal of Obstetrics & Gynaecology. 2020 Mar 14;127(4):478–88.

29. Brownfoot FC, Tong S, Hannan NJ, Binder NK, Walker SP, Cannon P, et al. Effects of Pravastatin on Human Placenta, Endothelium, and Women with Severe Preeclampsia. Hypertension. 2015 Sep 14;66(3):687–97.

30. Kumasawa K, Ikawa M, Kidoya H, Hasuwa H, Saito-Fujita T, Morioka Y, et al. Pravastatin induces placental growth factor (PGF) and ameliorates preeclampsia in a mouse model. Proceedings of the National Academy of Sciences of the United States of America. 2011 Jan 25;108(4):1451–5.

31. Cluver C, Hannan N, van Papendorp E, Hiscock R, Mol B, Theron G, et al. The Pre-eclampsia Intervention with Esomeprazole trial (PIE trial). American Journal of Obstetrics and Gynecology. 2018 Jan 1;218(1):S26–7.

32. Onda K, Tong S, Beard S, Binder N, Muto M, Senadheera SN, et al. Proton pump inhibitors decrease soluble fms-like tyrosine kinase-1 and soluble endoglin secretion, decrease hypertension, and rescue endothelial dysfunction. Hypertension. 2017;69(3):457–68.

33. Turanov AA, Lo A, Hassler MR, Makris A, Ashar-Patel A, Alterman JF, et al. RNAi Modulation of Placental sFLT1 for the Treatment of Preeclampsia. Nature biotechnology. 2018 Dec 1;36(12):1164–73.

34. da Silva GM, da Silva MC, Nascimento DVG, Lima Silva EM, Gouvêa FFF, de França Lopes LG, et al. Nitric Oxide as a Central Molecule in Hypertension: Focus on the Vasorelaxant Activity of New Nitric Oxide Donors. Biology. 2021 Oct 1;10(10).

35. Bergaya S, Meneton P, Bloch-Faure M, Mathieu E, Alhenc-Gelas F, Lévy BI, et al. Decreased flow-dependent dilation in carotid arteries of tissue kallikrein-knockout mice. Circulation research. 2001 Mar 30;88(6):593–9.

36. Thuringer D, Maulon L, Frelin C. Rapid Transactivation of the Vascular Endothelial Growth Factor Receptor KDR/Flk-1 by the Bradykinin B2 Receptor Contributes to Endothelial Nitric-oxide Synthase Activation in Cardiac Capillary Endothelial Cells. Journal of Biological Chemistry. 2002 Jan 18;277(3):2028–32.

37. Yao YY, Yin H, Shen B, Smith RS, Liu Y, Gao L, et al. Tissue kallikrein promotes neovascularization and improves cardiac function by the Akt-glycogen synthase kinase-3beta pathway. Cardiovascular research. 2008 Dec;80(3):354–64.

38. Bader M. Kallikrein-kinin system in neovascularization. Arteriosclerosis, thrombosis, and vascular biology. 2009 May;29(5):617–9.

39. Huang S, Chen M, Yu H, Lin K, Guo Y, Zhu P. Co-expression of tissue kallikrein 1 and tissue inhibitor of matrix metalloproteinase 1 improves myocardial ischemia-reperfusion injury by promoting angiogenesis and inhibiting oxidative stress. Molecular Medicine Reports. 2021 Feb 1;23(2).

40. Fu C, Li B, Sun Y, Ma G, Yao Y. Bradykinin inhibits oxidative stress-induced senescence of endothelial progenitor cells through the B2R/AKT/RB and B2R/EGFR/RB signal pathways. Oncotarget. 2015;6(28):24675–89.

41. Kayashima Y, Smithies O, Kakoki M. The kallikrein-kinin system and oxidative stress. Current opinion in nephrology and hypertension. 2012 Jan;21(1):92–5.

42. Barros CC, Haro A, Russo FJ, Schadock I, Almeida SS, Reis FC, et al. Bradykinin inhibits hepatic gluconeogenesis in obese mice. Laboratory investigation; a journal of technical methods and pathology. 2012 Oct;92(10):1419–27.

43. Stewart LA, Simmonds M, Duley L, Llewellyn A, Sharif S, Walker RA, et al. Evaluating Progestogens for Preventing Preterm birth International Collaborative (EPPPIC): meta-analysis of individual participant data from randomised controlled trials. The Lancet. 2021 Mar 27;397(10280):1183–94.

44. Bakker R, Pierce S, Myers D. The role of prostaglandins E1 and E2, dinoprostone, and misoprostol in cervical ripening and the induction of labor: a mechanistic approach. Archives of gynecology and obstetrics. 2017 Aug 1;296(2):167–79.

45. Yuan C, Yao Y, Fu C, Rong T, Li B, Carvalho A, et al. Plasma concentrations of tissue kallikrein in normal and preeclamptic pregnancies. Hypertension in pregnancy. 2020 Jan 2;39(1):64–9.

46. Khedun SM, Naicker T, Moodley J. Tissue kallikrein activity in pregnancy. Australian and New Zealand Journal of Obstetrics and Gynaecology. 2000;40(4):451–4.

47. de Zafra CLZ, Sasseville VG, Matsumoto S, Freichel C, Milton M, MacLachlan TK, et al. Inflammation and immunogenicity limit the utility of the rabbit as a nonclinical species for ocular biologic therapeutics. Regulatory toxicology and pharmacology : RTP. 2017 Jun 1;86:221–30.

48. Stills HF. Polyclonal antibody production. In: Sucko MA, Stevens KA, Wilson RP, editors. The Laboratory Rabbit, Guinea Pig, Hamster, and Other Rodents. Academic Press; 2012. p. 259–74.

49. Duffy JMN, Cairns AE, Richards-Doran D, van 't Hooft J, Gale C, Brown M, et al. A core outcome set for pre-eclampsia research: an international consensus development study. BJOG: An International Journal of Obstetrics & Gynaecology. 2020 Nov 1;127(12):1516–26.

50. Hall DR. Understanding expectant management of pre-eclampsia. Obstetrics and Gynaecology Forum. 2016;26:22–7.

51. Idänpään-heikkilä JE. WHO Guidelines for Good Clinical Practice (GCP) for Trials on Pharmaceutical Products: Responsibilities of the Investigator. Ann Med [Internet]. 1994 Jan 8 [cited 2018 Feb 22];26(2):89–94. Available from: http://www.tandfonline.com/doi/full/10.3109/07853899409147334

52. Spencer RN, Hecher K, Norman G, Marsal K, Deprest J, Flake A, et al. Development of standard definitions and grading for Maternal and Fetal Adverse Event Terminology. Prenat Diagn. 2022;42(1).

53. Salaets T, Turner MA, Short M, Ward RM, Hokuto I, Ariagno RL, et al. Development of a neonatal adverse event severity scale through a Delphi consensus approach. Arch Dis Child. 2019;104(12).

54. Wheeler GM, Mander AP, Bedding A, Brock K, Cornelius V, Grieve AP, et al. How to design a dose-finding study using the continual reassessment method. BMC Medical Research Methodology. 2019 Jan 18;19(1):1–15.

55. Bergman L, Bergman K, Langenegger E, Moodley A, Griffith-richards S, Wikström J, et al. PROVE—Pre-Eclampsia Obstetric Adverse Events: Establishment of a Biobank and Database for Pre-Eclampsia. Cells. 2021 Apr 20;10(4):959.
