# Supplementary material for: Safety and potential efficacy of DM199, a tissue kallikrein-1 analogue, for treating pre-eclampsia and fetal growth restriction: study protocol for a South African, hospital-based phase I/II open-label trial
Source: BMJ Open. 2025 Dec 17;15(12):e104035. doi: 10.1136/bmjopen-2025-104035 (PMC12716557; doi:10.1136/bmjopen-2025-104035)
Supplement: online supplemental file 2 [file bmjopen-15-12-s002.pdf]

PARTICIPANT INFORMATION LEAFLET AND CONSENT FORM
Version 1.1 1 March 2025

DM199 for Pregnancy Complications	
**Principle Investigator**	Professor Cathy Cluver
**Contact details:**	Email: cathycluver@sun.ac.za<br>Phone: 0823210298<br>Address: Department of Obstetrics and Gynaecology,<br>Faculty of Medicine and Health Sciences,<br>Stellenbosch University, Tygerberg Campus,<br>Francie van Zyl Drive, Tygerberg , 7505
**Ethics reference number:**<br>**Project ID:**	Project Id: 30213<br>Ethics Reference No: M24/04/009

Invitation to be involved in a research study

The Preeclampsia Research Unit at Stellenbosch University would like to invite you to take part in a research project. Please take some time to read the information presented here, which will explain the details of this project. Please ask the study staff any questions about any part of this project that you do not fully understand. It is very important that you are completely satisfied that you clearly understand what this research entails and how you could be involved. Also, your participation is **entirely voluntary,** and you are free to decline to participate. In other words, you may choose to take part, or you may choose not to take part. Nothing bad will come of it if you say no: it will not affect you negatively in any way whatsoever. Refusal to participate will involve no penalty or loss of benefits or reduction in the level of care to which you are otherwise entitled. You are also free to withdraw from the study at any point, even if you do agree to take part initially.

The Health Research Ethics Committee at Stellenbosch University has approved this study**.** The study will be conducted according to the ethical guidelines and principles of the international Declaration of Helsinki, the South African Guidelines for Good Clinical Practice (2006), the Medical Research Council (MRC) Ethical Guidelines for Research (2002), and the Department of Health Ethics in Health Research: Principles, Processes and Studies (2015).

What is this research study all about?

Preeclampsia and fetal growth restriction are serious conditions that only occur only in pregnancy.

Preeclampsia is associated with high blood pressure and other organ injury. There may be high levels of protein in your urine that indicate kidney damage (proteinuria), or other organ damage. Left untreated, preeclampsia can lead to serious complications for both the mother and child. Fetal growth restriction is a condition where the unborn baby is much smaller than it should be for its age. The unborn baby is not growing as well as it should. This can be very dangerous for the unborn baby. There are no treatments for preeclampsia or fetal growth restriction except for birth of your baby.

In this study we will be testing a new drug to see if it can treat preeclampsia and/or fetal growth restriction.

The name of the drug is DM199. It is a copy of natural enzyme called kallikrein, that is already found in the human body. When kallikrein is released into the body it causes blood vessels to relax and results in decreased blood pressure. As DM199 decreases blood pressure and improves the health of blood vessels it may be a treatment for preeclampsia and fetal growth restriction. Studies of DM199 in animals (including reproduction studies) and humans have shown that DM199 is safe and well tolerated. DM199 has been studied in healthy volunteers, people with kidney disease and people who have had a stroke.

The aim of the study is to see if DM199 can treat preeclampsia and/or fetal growth restriction. This study is only being run at Tygerberg Hospital. We will include up to 132 pregnant mothers with preeclampsia and or fetal growth restriction. If you decide to take part in the trial, you will be treated with DM199. We may need to put up a drip to give you the medication. We will draw blood samples (up to 10mls which is 2 teaspoons) to check the level of the medication in your blood and monitor your health. We may urine samples (up to 10mls which is 2 teaspoons). We will do ultrasounds to check your health and the health of your baby. We will also monitor you and your baby's health very closely. More information is provided below.

Why am I invited to participate?

You are being invited to participate in this trial because you have been admitted to Tygerberg Hospital with preeclampsia and/or for fetal growth restriction.

What will my responsibilities be?

If you decide to be in the trial, you will need to sign this informed consent document. This form will say that you want to be involved in the study and that you have read and understood the information about the trial. A study nurse and a doctor will then come and see you. The study nurse will stay with you while you are treated with the medication and will monitor your and your baby's condition closely.

You may need to have a drip put up in your arm. This is to enable us to give you the treatment and to draw blood samples. The nurse will monitor your baby with a machine that keeps track of your baby's heart rate and monitors your womb for contractions. You may have an ultrasound scan done of your baby before and after giving you the medication. We may measure the blood flow in your arm to check how well your blood vessels are functioning before and after treatment. We may do an ultrasound to check the blood flow in your brain by holding an ultrasound probe on the side of your head. We will measure your blood pressure and heart rate very carefully. We will collect information on your medical history. We may collect samples of your urine to check to see how your kidneys are functioning. After your baby is born, and the afterbirth is removed, we will collect a sample of blood from the cord attached to the afterbirth (the afterbirth and cord is usually thrown away after delivery). We will also ask if we can collect a small sample of your breastmilk (less than 2mls/ half a teaspoon). We will also follow you up after the delivery of the baby and will phone you to find out how you and the baby are doing. Blood samples will be sent overseas to countries like America, Sweden and Australia. They have the special equipment needed to measure the medication in the blood and measure markers of disease.

Will I benefit from taking part in this research?

If the medication does treat preeclampsia you may benefit as it could lower your blood pressure and increase the blood supply to your baby. If the medication does not work, there will be no benefit to you personally for being in the study. By being involved you may help us find a treatment for preeclampsia and/or fetal growth restriction which could help many pregnant mothers in your situation in the future.

Are there any risks involved in your taking part in this research?

Side effects may include headache, nausea, dizziness and low blood pressure. There is also a small chance of redness around the site of the injection. The study team will monitor you very closely for any side effects and will provide treatment if any is needed.

If I do not agree to take part, what alternatives do I have?

It is voluntary to be in the study and it is your choice to be involved or to not be involved. Your overall treatment will not be any different if you are not involved in the study. You can decide at any stage in the pregnancy to withdraw from the study and you will not have to give a reason for why you want to withdraw.

Who will have access to my medical records?

All information collected in the study will be kept strictly confidential. Identifying information (like your name, address and telephone number) will only be available to the research team (research nurses and doctors) at Tygerberg Hospital. Your will be given a study number. Your name will not be used for identifying any of your date or samples. The data collected will be stored in a secure electronic database hosted by Stellenbosch University. Any paperwork will be stored in a secure locked location. Only people directly involved in the study will have access to this information. Study monitors, the Data Safety and Monitoring Committee and the Sponsor will have access to the information on a confidential basis.

Your name or any personal details will not appear on any presentations or publications relating to this study. None of the laboratory staff will have access to your name or contact details.

What will happen with the biological samples that are collected?

Your blood, urine and breastmilk samples will be labelled with only your study number and not your name. These samples will be stored in a secure location at Stellenbosch University until we are ready to analyse them. The analyses may be performed in America, Sweden and Australia and we may need to ship your samples there. These countries have the expertise and experience to analyse these samples.

The samples will be used to measure the concentration of DM199 medication and to assess for disease markers. If you give permission below, we may store these samples for other analyses assessing biological markers for preeclampsia and fetal growth restriction. Once these studies are completed, your samples will be destroyed.

Even though it is unlikely, what will happen if you get injured somehow because you took part in this research study?

Stellenbosch University will provide comprehensive no-fault insurance and will pay for any medical costs that came about because you took part in the research (either because you used the medicine in this study or took part in another way). You will not need to prove that the sponsor was at fault.

If in the unlikely event you do get injured, the sponsor will reimburse you for all medical expenses without you having to prove that the sponsor was at fault.

You may also claim for emotional pain and suffering if you choose to. In this event, you will have to prove that the sponsor and/ or researcher was negligent and did not take all reasonable and foreseeable steps to prevent the injury or emotional trauma. This will be a separate legal matter.

Are there any costs involved if I decide to participate/take part?

The researchers are responsible for all direct study-related costs. You will not have to pay for anything related to the research if you do take part.
You will be compensated for your time and inconvenience. We will reimburse you for 8 hours of your time per day at R50 per hour. Therefore, for each day you are involved in the research we will reimburse you R400. If we perform blood sampling and/or do extra studies like ultrasound examinations, we will reimburse you a further R400 a day for each day. We will not reimburse you for travel as you will already be admitted to hospital for the birth of your baby.

Is there anything else that I should know or do?

You can phone Prof Cathy Cluver at 0823210298 if you have any further queries or encounter any problems. You can phone the Health Research Ethics Committee at 021 938 9677/9819 if there still is something that the researcher has not explained to you, or if you have a complaint. You will receive a copy of this information and consent form for you to keep safe.

If you have questions about this trial, you should first discuss them with your doctor or the Ethics Committee (contact details as provided on this form). After you have consulted your doctor or the Ethics Committee and if they have not provided you with answers to your satisfaction, you should write to the South African Health Products Regulatory Authority (SAHPRA) at:
The Chief Executive Officer
South African Health Products Regulatory Authority
Loftus Park
Building A
402 Kirkness Street
Arcadia, Pretoria
0083
E-mail: Boitumelo.Semete@sahpra.org.za
Tel: 012 501 0413

Declaration by participant

By signing below, I …………………………………………….. agree to take part in a research study entitled DM199 for Pregnancy complications.

I declare that:

- I have read this information and consent form, or it was read to me, and it is written in a language in which I am fluent and with which I am comfortable.
- I have had a chance to ask questions and I am satisfied that all my questions have been answered.
- I understand that taking part in this study is **voluntary,** and I have not been pressurised to take part.
- I understand that research is separate to my medical care or treatment and that refusing to take part in research does not mean I will no longer receive medical care.
- I may choose to leave the study at any time and nothing bad will come of it – I will not be penalised or prejudiced in any way.
- I may be asked to leave the study before it has finished, if the study doctor or researcher feels it is in my best interests, or if I do not follow the study plan that we have agreed on.

Signed at (*place*) .... on (*date*) ……………………. 20…..

....	....
**Signature of participant**	**Signature of witness**

Declaration by investigator

I *(name)* ………………………………………………….. declare that:

- I explained the information in this document in a simple and clear manner to ………………………………..
- I encouraged him/her to ask questions and took enough time to answer them.
- I am satisfied that she completely understands all aspects of the research, as discussed above.
- I did/did not use an interpreter. (*If an interpreter is used then the interpreter must sign the declaration below.)*

Signed at (*place*) .... on (*date*) ……………………. 20…..

....	....
**Signature of investigator**	**Signature of witness**

Permission to have all anonymous data shared with journals

Please carefully read the statements below (or have them read to you) and think about your choice. No matter what you decide, it will not affect whether you can be in the research study, or your routine health care.

When this study is finished, we would like to publish results of the study in journals. Most journals require us to share your anonymous data with them before they publish the results. Therefore, we would like to obtain your permission to have your anonymous data shared with journals. In accordance with the POPI Act, the researchers will take care to ensure that you are not identifiable (all personal information is not linked to the data shared.

Permission for sharing samples and/or information with other investigators

Please carefully read the statements below (or have them read to you) and think about your choice. No matter what you decide, it will not affect whether you can be in the research study, or your routine health care.

In order to do the *research* we have discussed, we must collect and store blood (up to 10mls, which is 2 teaspoons, each time a sample is taken) and urine (up to 10mls, which is 2 teaspoons) . We will do some of the tests right away. Other tests may be done in the future. Once we have done the research that we are planning for this research project, we would like to store your sample and/or information. Other investigators from our team who are based all over the world (including in America, Sweden and Australia) can ask to use these samples in future research. This may be on samples that are stored overseas. To protect your privacy, we will replace your name with a unique study number. We will only use this code for your sample and information about you. We will do our best to keep the code private. It is however always possible that someone could find out about your name, but this is very unlikely to happen. Therefore, we would like to ask for your permission to share your samples and information with other investigators.

Permission to store samples and/or information for future studies

Please carefully read the statements below (or have them read to you) and think about your choice. No matter what you decide, it will not affect whether you can be in the research study, or your routine health care.

As you are aware technology is constantly changing and so tests that may not be available at the time of this research may be possible in the future. As researchers learn more about illnesses or diseases, new research can be done using existing samples instead of returning to participants to ask for additional samples. In order to do further *research* in the future, we would like to ask your permission to store the blood samples and urine described above and health information from people like you with preeclampsia and/or fetal growth restriction. Any future studies or reuse of samples or data will need to be approved by the Stellenbosch University Health Research Ethics Committee.

Tick the Option you choose for anonymous data sharing with journals

I agree to have my anonymous data shared with journals during publication of results of this study

☐ Signature____________

OR

I do not agree to have my anonymous data shared with journals during publication of results of this study

☐ Signature____________

Tick the Option you choose for sharing samples and/or information with other investigators

I do not want my sample and/or information to be shared with other investigators

☐ Signature_______________

OR

My sample and/or information may be shared with other investigators who are able to conduct further analysis in … [*describe the field of your study, e.g., diabetes research*]

☐ Signature_______________

Tick the Option you choose for storage and reuse of samples/data for studies in the future

I do not want my sample(s) and/or information (data) to be stored for reuse for future studies

☐ Signature_______________

OR

I hereby agree that my sample(s) and/or information (data) may be stored for future research in a field related to … [*describe the field of your study, e.g., diabetes research*]

☐ Signature_______________
