# Supplementary material for: Safety and potential efficacy of DM199, a tissue kallikrein-1 analogue, for treating pre-eclampsia and fetal growth restriction: study protocol for a South African, hospital-based phase I/II open-label trial
Source: BMJ Open. 2025 Dec 17;15(12):e104035. doi: 10.1136/bmjopen-2025-104035 (PMC12716557; doi:10.1136/bmjopen-2025-104035)
Supplement: online supplemental file 3 [file bmjopen-15-12-s003.docx]

**Supplementary Table 1: Trial schedule**

|  |  |  | | **Collection Time-Point (Collection time is calculated form the start of dosing)** | | | | | | | | | | | | | | |  |  |  |  |  |
| --- | --- | --- | --- | --- | --- | --- | --- | --- | --- | --- | --- | --- | --- | --- | --- | --- | --- | --- | --- | --- | --- | --- | --- |
|  | **Screening** | **Pre-Dose** | **Day 1 Dosing** | **Day 1 Period After Infusion Start Time** | | | | | | | | | | | | | | **Day 2** | **Day 3** | **Day 4***  **(if still gravid)** | **Delivery** | **Discharge** | **6 weeks postpartum** |
|  | **Days 1** | **Day 1 Pre-Dose** | **Day 1 Dosing** | **5 (+/- 2) min** | **10 (+/- 2) min** | **15 (+/- 2) min** | **20 (+/- 2 min)** | **30 (+/- 2 min)** | **40 (+/- 2 min)** | **50 (+/- 2 min)** | **60 (+/- 5 min)** | **90 (+/- 5 min)** | **2 hr (+/- 10 min)** | **3 hr (+/- 10 min)** | **4 hr (+/- 10 min)** | **8 hr (+/- 10 min)** | **12 hr (+/- 10 min)** | **24 hr  (+/- 10 min)** | **48 hr  (+/- 60 min)** | **72 hrs**  **(+/- 60 min)** |  |  |  |
| Informed Consent | X |  |  |  |  |  |  |  |  |  |  |  |  |  |  |  |  |  |  |  |  |  |  |
| Medical History | X |  |  |  |  |  |  |  |  |  |  |  |  |  |  |  |  |  |  |  |  |  |  |
| Previous and Concomitant Medication | X |  |  |  |  |  |  |  |  |  |  |  |  |  |  |  | X | X | X | X | X | X | X |
| Physical Exam | X |  |  |  |  |  |  |  |  |  |  |  |  |  |  |  |  | X | X | X |  |  |  |
| Body Weight & Height (including BMI) | X |  |  |  |  |  |  |  |  |  |  |  |  |  |  |  |  |  |  |  |  |  |  |
| Urine protein creatinine ratio | X |  |  |  |  |  |  |  |  |  |  |  |  |  |  |  |  | X | X | X |  |  |  |
| Blood Pressure, heart rate and monitoring uterine activity | X | X |  | X | X | X | X | X | X | X | X | X | X | X | X | X | X | X | X | X |  |  |  |
| Fetal ultrasound |  | X |  |  |  |  |  |  |  |  |  |  | X |  |  |  |  | X | X | X |  |  |  |
| Endothelial function testing** |  | X |  |  |  |  |  |  |  |  |  |  | X |  |  |  |  | X | X | X |  | X |  |
| Cerebral autoregulation** |  | X |  |  |  |  |  |  |  |  |  |  | X |  |  |  |  | X | X | X |  | X |  |
| Ophthalmic artery Doppler |  | X |  |  |  |  |  |  |  |  |  |  | X |  |  |  |  | X | X | X |  |  |  |
| Study drug administration-DM199 IV |  |  | X |  |  |  |  |  |  |  |  |  | X |  |  |  |  |  |  |  |  |  |  |
| Study drug administration-DM199 SC |  |  |  |  |  |  |  |  |  |  |  |  | X |  |  |  |  |  |  | X |  |  |  |
| Blood sampling for PK of DM199 (Part 1) |  | X |  | X | X | X | X | X | X | X | X | X | X | X | X | X | X | X | X | X |  |  |  |
| Blood sampling for (Part 2) | X | X |  | X |  | X |  | X |  | X |  | X |  |  | X |  | X | X | X |  |  |  |  |
| Colostrum/ Breastmilk collection*** |  |  |  |  |  |  |  |  |  |  |  |  |  |  |  |  |  | X |  |  |  |  |  |
| Adverse Event Monitoring | X | X | X | X | X | X | X | X | X | X | X | X | X | X | X | X | X | X | X | X | X | X | X |

*For part 2.2 and 2.3 of the study, the participants may continue their pregnancies well beyond four days.

**This will be performed once a therapeutic dose is found

***A colostrum/breastmilk sample will be collected within 24 hours of delivery once a therapeutic dose is found

**Supplementary Table 2: Grading of Adverse Events**

| **Systemic (General)** | **Mild (Grade 1)** | **Moderate (Grade 2)** | **Severe (Grade 3)** | **Potentially Life Threatening**  **(Grade 4)** |
| --- | --- | --- | --- | --- |
| Nausea/ vomiting | No interference with activity or 1 – 2 episodes/24 hrs | Some interference with activity or > 2 episodes/24 hrs | Prevents daily activity, requires outpatient IV hydration | Hypotensive shock or potentially life threatening |
| Diarrhoea | 2 – 3 loose stools or  <400 grams/24 hrs | 4 – 5 stools or  400 – 800 grams/24 hours | 6 or more watery stools or > 800gms/24 hours or requires outpatient IV hydration | Hypotensive shock or potentially life threatening |
| Headache | No interference with activity | Repeated use of non-narcotic pain reliever >24 hours or some interference with activity | Significant; any use of narcotic pain reliever or prevents daily activity | Potentially life threatening |
| Fatigue | No interference with activity | Some interference  with activity | Significant; prevents daily activity | Potentially life threatening |
| Myalgia | No interference with activity | Some interference with activity | Significant; prevents daily activity | Potentially life threatening |
| Illness or clinical adverse event | No interference with activity | Some interference with activity not requiring medical intervention | Prevents daily activity and requires medical intervention | Potentially life threatening |
| Facial, oral or airway angioedema | Absent | Absent | Intervention indicated | Life-threatening and urgent intervention is indicated |
| Flushing | Asymptomatic, clinical or diagnostic observation only | Moderate symptoms | Symptomatic associated with hypotension and/or tachycardia |  |
| Hypotension | Asymptomatic, intervention not indicated | Non-urgent medical intervention indicated | Medical intervention or hospitalization indicated | Life-threatening and urgent intervention indicated |
| Orthostatic hypotension | Mild unsteadiness or sensation of movement | Moderate unsteadiness or sensation of movement. Near fainting | Fainting, orthostatic collapse |  |
| **Local injection site reactions** | | | | |
| Pain | Mild pain | Moderate pain | Severe pain | ED visit or hospitalization |
| Tenderness | Mild discomfort to touch | Discomfort with movement | Significant discomfort at rest | ED visit or hospitalization |
| Pruritis | Mild or localized | Intense or widespread; intermittent | Intense or widespread; constant | ED visit or hospitalization |
| Urticaria (hives, welts, wheals) | Urticarial lesions cover <10 BSA | Urticarial lesions covering 10-30% BSA | Urticarial lesions covering >30%BSA | ED visit or hospitalization |
| Erythema/Redness | Target lesions cover <10% BSA | Target lesions covering 10-30% BSA and associated with skin tenderness | Target lesions covering >30% BSA and associated with oral or genital erosions | Target lesions covering >30% BSA; associated with fluid or electrolyte abnormalities; ICU care or burn unit indicated |
| Induration/  Swelling | Mild induration, able to move skin parallel to plane (sliding) and perpendicular to skin (pinching up) | Moderate induration, able to slide skin, unable to pinch skin | Severe induration, unable to slide or pinch skin, limiting joint movement or orifice (e.g., mouth, anus) | Generalized; associated with signs of symptoms of impaired breathing or feeding |

**Abbreviations:** Emergency Department-ED; Body surface area-BSA

**Supplementary Table 3: Other ongoing or completed trials of DM199**

| **COMPLETED STUDIES** | | | | | | |
| --- | --- | --- | --- | --- | --- | --- |
| **Study Number and Location** | **Design** | **Aims** | | **Dosing regime** | | **Participants Enrolled** |
| DMA-Clin-199-2013-001  Netherlands  NCT01845064 (Part, A,B and C) | Phase 1/2a, double-blinded, placebo controlled single site study | Evaluate safety and tolerability  Pharmacokinetics of subcutaneous DM199 | | Part A: healthy volunteers  DM199: 1.5, 5, 15, 30, 50 μg/kg or placebo SC  Each subject received both a dose of DM199 and placebo  Single dose of either 3 or 15 μg/kg SC or placebo | | DM199: 32  Placebo: 32 |
|  |  |  |  | Part B: Diabetic subjects  DM199 0.3, 1.5, 15 μg/kg or placebo SC as a single-ascending dose | | DM199 11  Placebo 11 |
|  |  |  |  | Part C: healthy volunteers  DM199 3 μg/kg or placebo once every 72 hours for a total of 6 doses over 16 days SC  DM199 15 μg/kg on Days 1, 4, and 7 and DM199 25 μg/kg on Days 10, 13, and 16 for a total of 6 doses of DM199 SC | | DM199 12  Placebo 6 |
|  |  |  |  | Part D: diabetic subjects  DM199 3 or 15 μg/kg or placebo SC once every 72 hours for a total of 10 doses over 28 days | | DM199 25  Placebo 12 |
| DM199-2016-001  Australia  Single study site  NCT02868996  DOI:  <https://doi.org/10.18203/2349-3259.ijct20174861> | Open label, Phase 1b, ascending IV dose administration and comparative pharmacokinetic study of IV and SC DM199 administration  Study duration: 32 days | Evaluate safety and tolerability of single IV dose administration and comparative bioavailability of SC and IV administration | | Part A: DM199 0.25, 0.5, 0.75, and 1.0 µg/kg IV single 30-minute infusion | | DM199: 12 |
|  |  |  |  | Part B: B single 0.75 µg/kg IV dose or a single 3.0 µg/kg dose SC | | DM199 24 |
| DM199-2017-001 (ReMEDy 1)  Australia  12 Sites  NCT03290560 | Phase 2 randomized,  double-blind, placebo-controlled, trial  Study duration: 90 days | Assess the safety and tolerability of single IV dose followed by SC dose administration of DM199 in subjects with acute ischaemic stroke. | | DM199 1 μg/kg IV 40-minute infusion (polyolefin bag) followed by 3 μg/kg SC dose 2-12 hours after IV infusion and then 3 ug/kg SC dose every 72 hours over 22 days for a total of eight SC doses  OR  Identical placebo | | DM199: 46  Placebo: 45 |
| DM199-2018-001  U.S.  3 Sites | Phase 1b, multi-center,  open-label, dose ranging study in subjects with chronic kidney disease and diabetes  Study duration: 11 days | Evaluate safety and tolerability  Pharmacokinetics of three subcutaneous dose levels in Type 1 or Type 2 diabetes and chronic kidney disease | | Type 1 or 2 diabetes and CKD Stage 3: 3, 5, or 8 μg/kg SC  Type 1 or 2 diabetes and CKD Stage 4: 3 μg/kg SC | | DM199: 33 |
| DM199-2019-001  U.S.  15 sites  NCT04123613 | Phase 2, multi-center, open label, basket study  Study duration: 16 weeks | Evaluate safety and efficacy of two SC doses of DM199 in three different stage 2 chronic kidney disease subpopulations | | SC dose of DM199: 2.0 or 5.0 µg/kg SC twice weekly for 95 days | | 1) 24 African American participants with hypertension  2) 25 participants with IgA nephropathy  3) 35 participants with Type 2 diabetes |
| DM199-2023-001  Australia  Single site  PMID: 40237565 | Phase 1c, Open Label, Single Ascending Dose Study | Evaluate safety, tolerability, and pharmacokinetics of DM199 Administered IV with PVC Bag in Adult | | Part A:  3 healthy participants receiving 0.1 μg/kg DM199 in an infusion starting at 35 mL/hr for 15 minutes and if tolerated, then increased to maximum of 75 mL/hr to complete a 50 mL infusion in approximately 50 minutes.  Sequential planned cohorts of 3 participants receiving escalating dose of DM199 up to 0.5 μg/kg IV given over an approximately 50-minute period (35 mL/hr first 15 minutes and if tolerated, increased to maximum of 75 mL/hr to complete 50 mL infusion), not to exceed a total of 50 μg of DM199  Part B:  Cohort of 3 participants recently started on ACE inhibitor medications with a last dose >24 hours prior to IV start)  35 mL/hr first 15 minutes and if tolerated increased to maximum of 75 mL/hr to complete 50 mL infusion | | Part A: Healthy participants (9)  Part B  Adults recently taking ACE Inhibitors (3) |
| **ONGOING STUDIES** | | | | | | |
| DM199-2021-001 (ReMEDy 2)  U.S.  70 Sites  NCT05065216 | Phase 2/3 adaptive design, randomized, double-blind, placebo-controlled  trial  Study Duration: 90 days | | Evaluate safety, tolerability, and efficacy for treatment of acute ischaemic stroke | IV dose of 0.5 µg/kg at 35 mL/hr for the first 15 minutes of the infusion followed by an increase to 75 mL/hr to complete the IV infusion, only if no hypotension occurs during the first 15 minutes and then SC dose of 3 ug/kg twice per week over 21 days for a total of 7 SC doses.  OR  Matching placebo | 364 subjects with acute ischaemic stroke unable to receive thrombolytics or mechanical thrombectomy | |

**Supplementary table 4: Specific Inclusion and exclusion criteria, aims, sample size and outcomes for the sub-studies**

|  | **Inclusion Criteria** | **Aim** | **Sample size** | **Outcomes** |  |
| --- | --- | --- | --- | --- | --- |
| **Part 1:**  **Open label phase 1B** Ascending dose finding study | Women with Pre-eclampsia  Systolic BP ≥150mmHg and/or a diastolic BP ≥100mmHg  Delivery planned within 72hrs  GA between 27d + 0 w and 42d + 0w | Determine a safe and effective dose of DM199 | Traditional dose escalation 3 + 3 design  Once an upper dose is identified, we will maintain the same dose and recruit a further 6 with preeclampsia <34 weeks, and 6 with preeclampsia at 34 weeks gestation or above.  Maximum 42 participants | Primary  Secondary  Exploratory | Safety: Incidence of treatment emergent adverse events  DM199 umbilical cord blood levels at birth  Efficacy: Change in maternal blood pressure†  (baseline to immediately after the completion of  the infusion, 30 minutes post-infusion and 24 hours  after the IV dose)  Change in maternal BP from baseline to delivery  Pharmacokinetic profile of DM199  Uterine contractions  Maternal feedback on tolerability  Severe hypertension and/or hypotension  Use of antihypertensive agents  Changes in uterine, ophthalmic and fetal Doppler parameters  Changes clinical biomarkers of disease severity^‡^  Adverse maternal and perinatal outcomes^§^  Changes in flow medicated blood vessel dilatation and changes in cerebral autoregulation |
| **Part 2.1**  **Open label phase II**  Women with Preeclampsia requiring delivery within 72 hours | Women with Pre-eclampsia  Systolic BP ≥150mmHg and/or a diastolic BP ≥100mmHg  Delivery planned within 72hrs  GA between 27d + 0 w and 42d + 0w | Evaluate safety, tolerability and pharmacokinetics of DM 199 in women with pre-eclampsia requiring delivery within 72 hrs | 30 | Primary  Secondary  Exploratory | Efficacy: Change in maternal BP from baseline assessed immediately after the completion of the infusion, at 30 minutes post-infusion and 24 hours after the initial dose  Safety: Incidence of treatment emergent events  Umbilical cord blood levels of DM199 after birth  Uterine contractions  Episodes of severe hypertension or hypotension after administration of DM199  Use of other antihypertensive agents  Changes in uterine and ophthalmic artery Doppler parameters  Maternal feedback on tolerability  Changes in fetal Doppler parameters  Changes of clinical biomarkers of disease severity including haemoglobin, platelet, urea, creatinine and proteinuria levels  Adverse maternal and perinatal outcomes as defined by the Delphi consensus on preeclampsia adverse outcomes51  Sparse maternal pharmacokinetic profiling of DM199  Changes associated with clinical biomarkers of disease severity in blood and urine – e.g. haemoglobin, renal, liver function tests and proteinuria via (between maternal blood and urine samples collected just prior to administration and samples post administration).  Adverse maternal and perinatal/neonatal outcomes as defined by the Delphi consensus on preeclampsia adverse outcomes51  Changes in flow mediated blood vessel dilatation  Changes in cerebral autoregulation |
| **Part 2.2**  **Open label phase II** | Women with Preterm pre-eclampsia and deemed suitable by the clinical team for expectant management of preeclampsia: i.e. hold off delivery to gain gestation and reduce prematurity | Evaluate safety, tolerability and pharmacokinetics of DM 199 in women with preterm pre-eclampsia undergoing expectant management | 30 | Primary  Secondary  Exploratory | Efficacy: Prolongation of pregnancy  Change in 24-hr protein creatinine ratio one week after enrolment, compared to baseline values  Need to increase or decrease other antihypertensive agents  Safety: Incidence of treatment emergent adverse events  Umbilical cord blood levels of DM199 after birth  Change in maternal blood pressure from baseline  Number of women reaching 34+0 weeks gestation  Episodes of severe hypertension or hypotension  Uterine contractions  Changes in uterine artery or ophthalmic artery Doppler flow  Changes in fetal Doppler parameters  Neonatal length of stay at Tygerberg Hospital and overall in any hospital  Maternal feedback on tolerability  Sparse maternal pharmacokinetic profiling of DM199  Changes associated with clinical biomarkers of disease severity in blood and urine – e.g. haemoglobin, renal, liver function tests and proteinuria.  Adverse maternal and perinatal outcomes as defined by the Delphi consensus51  Changes in flow mediated blood vessel dilatation  Changes in cerebral autoregulation |
| **Part 2.3**  **Open label phase II** | Women with Fetal growth restriction, defined as less than the 3rd centile on fetal growth charts.  No evidence of fetal compromise that warrants immediate delivery.  GA between 27 + 0 weeks and 32 + 6 weeks.  There may be a co-existing diagnosis of preeclampsia, but they do not have to have preeclampsia. | Evaluate safety, tolerability and pharmacokinetics of DM 199 in women with preterm fetal growth restriction | 30 | Primary  Secondary  Exploratory | Efficacy: Changes in uterine artery and ophthalmic artery Doppler flow  Changes in fetal Doppler parameters  Birthweight centile  Safety: Incidence of treatment emergent adverse events  Umbilical cord blood levels of DM199 after birth  Prolongation of gestation (measured from time of first dose to delivery)  Fetal growth trajectory if 2 ultrasounds measuring fetal growth are done during the pregnancy. (We will plan to do a second fetal growth ultrasound to track fetal growth. However, this second ultrasound can only be done if the participant remains pregnancy for two weeks – the minimal interval period that growth ultrasounds can be performed)  Changes in maternal blood pressure  Use of antihypertensive medication (if unmedicated at enrolment or the need to increase or decrease other antihypertensive agents  Maternal feedback on tolerability  Sparse maternal pharmacokinetic profiling of DM199  Changes in flow mediated blood vessel dilatation  Changes in plasma biomarkers associated with fetal growth restriction  Adverse maternal and perinatal/neonatal outcomes |

**Abbreviations:** GA gestational age**;** BP blood pressure; w weeks; d days; hr hours; FGR feta growth restriction

***Maternal cardiac** disease including a significant arrhythmia, a conduction abnormality or severe valvular disease or congenital or acquired heart disease

**†**The average of 3 consecutive blood pressure measurements will be used at each timepoint

‡Changes clinical biomarkers of disease  severity  including haemoglobin, platelet, urea, creatinine and proteinuria levels **And** Changes in plasma biomarkers associated with endothelial dysfunction (between maternal plasma samples collected just prior to administration and samples post administration).

^§^ Adverse maternal and perinatal outcomes as defined by the Delphi consensus on preeclampsia adverse outcomes[^57^](#_ENREF_57)
