# Supplementary material for: Safety and potential efficacy of DM199, a tissue kallikrein-1 analogue, for treating pre-eclampsia and fetal growth restriction: study protocol for a South African, hospital-based phase I/II open-label trial
Source: BMJ Open. 2025 Dec 17;15(12):e104035. doi: 10.1136/bmjopen-2025-104035 (PMC12716557; doi:10.1136/bmjopen-2025-104035)
Supplement: Supplementary file 4 [file bmjopen-2025-104035.reviewer_comments.pdf]

PEER REVIEW HISTORY

BMJ Open publishes all reviews undertaken for accepted manuscripts. Reviewers are asked to complete a checklist review form and are provided with free text boxes to elaborate on their assessment. These free text comments are reproduced below.

ARTICLE DETAILS

Title (Provisional)

Safety and potential efficacy of DM199, a tissue kallikrein-1 analogue, for treating pre-eclampsia and fetal growth restriction: Study protocol for a South African, hospital-based phase I/II open-label trial

Authors

Cluver, Catherine Anne; VISSER, JACQUELINE; Hassim, Tasleem; van Greunen, Anriette; Budhram, Samantha; Knipe, Karusha; Decloedt, Eric; Brink, Lucy; Langenegger, Eduard; Bergman, Lina; Imberg, Henrik; Bekker, Adrie; Walker, Susan P; Tong, Stephen

---

VERSION 1 - REVIEW

Reviewer **1**

Name **Sande, Ragnar**

Affiliation **Stavanger University Hospital, Department of Obstetrics and Gynaecology**

Date **30-Jun-2025**

COI **None**

The protocol is well written and presents the proposed study clearly. The proposed study is important, as preeclampsia is a major cause for maternal and neonatal morbidity and mortality world wide, and treatment options are few. There are obvious ethical aspects of the study, but the study has obtained relevant ethical approval, and it is beyond the mandate of the journal to reevaluate this. There are conflicts of interest, with the financial lnks to DiaMedica. These are clearly stated and transparent, and hopefully will not affect the interpretation of the results.

All in all an important study, well described.

Reviewer **2**

Name **Beardmore-Gray , Alice**

Affiliation **King's College London, Women and Children's Health**

**Date**	**01-Jul-2025**
**COI**	**None**

Thank you for the opportunity to review this fantastic and very interesting manuscript.

Overall

This study is incredibly exciting and offers real hope for a disease-modifying therapeutic agent for pre-eclampsia, albeit in the early stages of development.

Only 2% of all clinical trials conducted, take place in Sub-Saharan Africa, and of these even fewer evaluate the major causes of maternal death.

Funders, pharmaceutical companies and researchers, are often reluctant to include pregnant women in clinical trials, particularly drug trials, and as a consequence women's health has suffered. Nowhere more so than in low and middle income countries where 95% of global maternal and perinatal deaths occur.

This protocol therefore serves as an excellent, and critically important example of how to design a clinical drug trial during pregnancy and I hope that the publication of this manuscript will help inspire and encourage others.

In terms of the protocol itself, I believe it is well-written, clear, and methodologically sound. I have added a few further comments below.

Introduction

The introduction concisely and expertly explains the substantial burden that pre-eclampsia presents, and the current lack of any curative treatment, therefore highlighting the critical importance of this study, which evaluates a potential disease-modifying agent. It also clearly explains the mechanism of action for DM199, and its potential relevance to the pre-eclampsia and fetal growth restriction pathway.

Methods

I note that this is a study protocol for an ongoing trial therefore requesting any changes to the methodology would be beyond the scope of this review.

I think the methods section is clear, and easy to understand.

I am interested to understand why the investigators have chosen 140/90 as the target blood pressure, not 135/85, and why the threshold for acute severe hypertension was chosen to be 150, not 160.

I note the inclusion of patient and public involvement in the design of the protocol. Whilst acknowledging that this trial is primarily to assess safety and potential efficacy, I wonder if the investigators have considered collecting additional process and implementation outcomes, as these are vitally important to consider right from the start

of the intervention pipeline and I would be very interested to see outcomes regarding the acceptability of the IV infusion/s/c injection to women, whether they perceived any discomfort or disadvantages, as well as implementation and cost measures such as storage of the drug, cost per dose, other resource use, time taken to administer etc.

The investigators mention clinical biomarkers as an outcome and I wonder whether this will include PLGF or SFLT-1?

I noticed a few small typos:

Line 138 small typo – related *to*

Line 224 small typo potential participant – plural? Or a potential participant?

Line 288 Serious adverse events appears twice

**Reviewer**	**3**
**Name**	**Hoffman, Matthew**
**Affiliation**	**Christiana Care Health System, obgyn**
**Date**	**28-Jul-2025**
**COI**	**None**

This thoughtful protocol reviews a novel therapuetic for the treatment of preeclampsia and fetal growth restriction. As a study protocol, the primary role of the reviewer is to ensure readability and anticipate potential questions that may arise in the outcome papers that will be reported later. As a general comment, the protocol is complete and thoughtfully written.

The following comments are designed to improve the description of the protocol:

1. It is unclear how a determination is made that the patient will be delivered in 72 hours clinically. Some sense of the guidance provided would be helpful as these will be used by clinicians in the future. Though there is some guidance provided in Table 1, this is not explicitly detailed.

2. It is unclear how fetal monitoring will be done beyond dopplers. Will NSTs be done? Other?

3. Preterm fetal growth restriction is defined well in table 2 but from a reader standpoint it would be helpful to have this written in the text what is preterm and the fact that you are using the 3rd% tile.

VERSION 1 - AUTHOR RESPONSE

Reviewer: 1 Dr. Ragnar Sande, Stavanger University Hospital

The protocol is well written and presents the proposed study clearly. The proposed study is important, as preeclampsia is a major cause for maternal and neonatal morbidity and mortality worldwide, and treatment options are few. There are obvious ethical aspects of the study, but the study has obtained relevant ethical approval, and it is beyond the mandate of the journal to reevaluate this. There are conflicts of interest, with the financial links to DiaMedica. These are clearly stated and transparent, and hopefully will not affect the interpretation of the results.

All in all an important study, well described.

A: Thank you

Reviewer: 2 Dr. Alice Beardmore-Gray , King's College London

Thank you for the opportunity to review this manuscript - this study is incredibly exciting and offers real hope for a disease-modifying therapeutic agent for pre-eclampsia. This protocol serves as an excellent, and critically important example of how to design a clinical drug trial during pregnancy and I hope that the publication of this manuscript will help inspire and encourage others. I look forward to seeing the results.

A: Thank you

Reviewer: 3 Dr. Matthew Hoffman, Christiana Care Health System

This thoughtful protocol reviews a novel therapeutic for the treatment of preeclampsia and fetal growth restriction. As a study protocol, the primary role of the reviewer is to ensure readability and anticipate potential questions that may arise in the outcome papers that will be reported later. As a general comment, the protocol is complete and thoughtfully written.

A: Thank you

The following comments are designed to improve the description of the protocol.

1. It is unclear how a determination is made that the patient will be delivered in 72 hours clinically. Some sense of the guidance provided would be helpful as these will be used by clinicians in the future. Though there is some guidance provided in Table 1, this is not explicitly detailed.

A: The dose escalation cohort (part 1) and the 2.1 sub-cohort are those that will be delivered within 72 hours. These are cases of preeclampsia where there has already been a decision to deliver before enrolment. The decision will have been made by the treating clinical team, not the investigators.

The window of time exists administer the drug before birth is because for such cases, acute hypertension needs to first be stabilised followed by planned induction of labour.

2. It is unclear how fetal monitoring will be done beyond Dopplers. Will NSTs be done? Other?

A: We have added the following text.

Page 11, line 173
" Continuous fetal heart rate monitoring will be performed until delivery."

3. Preterm fetal growth restriction is defined well in table 2 but from a reader standpoint it would be helpful to have this written in the text what is preterm and the fact that you are using the 3rd centile.

A: Thank you for this suggestion. We have added the following text.

Page 14, line 234 to 236
"Fetal growth restriction will be classified according to a recent Delphi consensus,[32] but will also require the estimated fetal weight to be less than the 3rd centile on fetal growth charts."

VERSION 2 - REVIEW

**Reviewer**	**3**
**Name**	**Hoffman, Matthew**
**Affiliation**	**Christiana Care Health System, obgyn**
**Date**	**07-Oct-2025**
**COI**	

No additional changes are required.
